# Advantages of score-based delirium detection compared to a clinical delirium assessment—a retrospective, monocentric cohort study

**DOI:** 10.1371/journal.pone.0259841

**Published:** 2021-11-29

**Authors:** Markus Jäckel, Nico Aicher, Xavier Bemtgen, Jonathan Rilinger, Viviane Zotzmann, Paul Marc Biever, Alexander Supady, Peter Stachon, Daniel Duerschmied, Tobias Wengenmayer, Christoph Bode, Dawid Leander Staudacher

**Affiliations:** 1 Faculty of Medicine, Department of Cardiology and Angiology I, Heart Center Freiburg University, University of Freiburg, Freiburg, Germany; 2 Faculty of Medicine, Department of Medicine III (Interdisciplinary Medical Intensive Care), Medical Center, University of Freiburg, University of Freiburg, Freiburg, Germany; Heidelberg University Hospital, GERMANY

## Abstract

**Purpose:**

Delirium is an underdiagnosed complication on intensive care units (ICU). We hypothesized that a score-based delirium detection using the Nudesc score identifies more patients compared to a traditional diagnosis of delirium by ICU physicians.

**Methods:**

In this retrospective study, all patients treated on a general medical ICU with 30 beds in a university hospital in 2019 were analyzed. Primary outcome was a documented physician diagnosis of delirium, or a delirium score ≥2 using the Nudesc.

**Results:**

In 205/943 included patients (21.7%), delirium was diagnosed by ICU physicians compared to 438/943 (46.4%; ratio 2.1) by Nudesc≥2. Both assessments were independent predictors of ICU stay (p<0.01). The physician diagnosis however was no independent predictor of mortality (OR 0.98 (0.57–1.72); p = 0.989), in contrast to the score-based diagnosis (OR 2.31 (1.30–4.10); p = 0.004). Subgroup analysis showed that physicians underdiagnosed delirium in case of hypoactive delirium and delirium in patients with female gender and in patients with an age below 60 years.

**Conclusion:**

Delirium in patients with hypoactive delirium, female patients and those below 60 years was underdiagnosed by physicians. The score-based delirium diagnosis detected delirium more frequently and correlated with ICU mortality and stay.

## Introduction

Delirium, also known as “acute encephalopathy,” is defined by the Diagnostic and Statistical Manual of Mental Disorders, Fifth Edition (DSM-5) criteria as disturbance in attention and awareness, which develops over a short period of time and is different from baseline attention and awareness [1]. Being a common complication on the intensive care unit (ICU), with an incidence varying between 22–83%, depending on the population and the assessment method used [2–6], delirium is associated with increased mortality as well as prolonged hospital and ICU stay, also resulting in increased hospital costs [7,8]. Yet delirium is still not addressed accordingly and is notoriously underdiagnosed [9–13]. Referring to the high rate of overseen delirium, guidelines recommend a screening for delirium on the ICU with a valid and reliable delirium-score [14].

Nurses are in touch with the patients 24 hours a day, have much more patient contact as the physicians and therefore play a critical role in delirium detection [13]. In our medical ICU on a tertiary university hospital, the Nursing Delirium screening scale (Nudesc) is assessed at least three times daily by especially trained nurses in all patients on our ICU. The Nudesc is evaluated in one to two minutes, can be performed easily and shows a high sensitivity and specificity in most studies (86% and 87% on internal medicine units; 98% and 87% on neurological and cardiologic wards; 83% and 81% on a surgical ICU; 89% and 89% on a general ICU; 42% and 98% on neurology and neurosurgery or surgical units) [15–20].

However, due to the lack of data on the validity of the Nudesc on the ICU, it has not yet been integrated into our standard operating procedure for the clinical diagnosis of delirium, which means that the diagnosis of delirium is determined by our ICU physicians only by clinical assessment in unawareness of the score. As the assessment of a detailed psychopathological status of the patient is not manageable in terms of time for an intensive care physician, we wanted to test whether the utilization of the Nudesc is superior to a diagnosis of delirium by ICU physicians to finally integrate the Nudesc in our standard operating procedure. While diagnosis of delirium according to the criteria given in DSM-V (Diagnostic and Statistical Manual of Mental Disorder, 5^th^ edition) is considered to be the gold standard, guidelines advocate a score-based diagnosis of delirium [21].

Aim of the present study therefore was to investigate the Nudesc as score-based delirium assessment tool on a medical ICU. We therefore analyzed and compared incidence, risk factors and prespecified subgroups of patients with delirium on our medical ICU using two definitions of delirium: First, delirium diagnosed by the treating medical physicians (“clinical diagnosis”) and second, delirium diagnosed by a positive Nudesc. We additionally aimed to identify prespecified subgroups in which a score-based diagnosis may be even more helpful.

## Methods

We conducted an investigator-initiated single-center retrospective registry study analyzing patients from the Freiburg delirium registry treated in 2019.

Analysis was blinded to patient identity and was covered by an ethics approval (Ethics Committee of the Albert-Ludwigs-University of Freiburg, file number 387/19). Since only retrospective data of an already performed intervention was collected, the informed consent was waived by the approval of the relevant ethic committee.

### Patient selection and data collection

All patients treated at the Interdisciplinary Medical Intensive Care Unit (MIT) at the Medical Center, University of Freiburg, Germany for more than 24 hours were included in the present analysis. The University of Freiburg Medical Center is a tertiary care hospital with a 30-bed medical intensive care unit. If delirium evaluation was not possible, patients were excluded: patients with transfer to other hospitals or death before extubation, patients with severe neurologic comorbidities or hypoxic brain dysfunction.

All outcome variables were evaluated by manual case-by-case review of medical and patient records. Since only data from the index hospital stay was evaluated, no patients were lost to a follow up. Registry was checked for data integrity and plausibility according to the RECORD recommendations for data clearing [22]. Some data (like laboratory tests) were not available for all patients for all time points. Therefore, we included the number of values available for every data point given in the tables.

ICU free days within 15 days after admission to the ICU were analyzed. ICU free days were counted as zero if the patient died within the first 15 days after admission. Acute kidney injury was defined as increase of serum creatinine of ≥ 0.3mg/dl or to ≥ 1.5 times baseline, according to the KDIGO (Kidney Disease Improving Global Outcome) guideline [23]. Severe shock was predefined as a norepinephrine dose of ≥1mg over at least 4 hours according to the definition used by Russel et al [24]. In order to consider also patients with cardiogenic shock, patients requiring two different catecholamines for more than 4 hour were also considered having severe shock. Primary cause of illness was adjudicated on a case-by-case basis in “cardiac” (acute myocardial infarction, transcatheter aortic valve implantation, resuscitation, cardiogenic shock, heart rhythm disturbances, pulmonary embolism), “respiratory” (pneumonia, acute respiratory distress syndrome, exacerbated chronic obstructive bronchitis, pneumothorax), “infectious” (pneumonia, urosepsis, sepsis with other focus) or “other” (hyponatremia, gastrointestinal bleeding, vasculitis, ketoacidosis, liver failure).

### Definition of delirium

Delirium is a common complication in daily practice on our ICU. According to local standard operating procedures, efforts are taken in any patient in order to prevent and treat delirium in an interdisciplinary team approach including nurses, physiotherapists and physicians.

The primary endpoint referred to as “clinical diagnosis of delirium” was defined as written diagnosis of delirium by an ICU physician. Delirium on our ICU is diagnosed according to Fong et al., which is based on the definition discussed in *Diagnostic and Statistical Manual of Mental Disorders*, *5th edition*. In order to detect the primary endpoint [25]. All medical records, including letters, physical examinations and shift reports, which were assessed at least three times daily by the treating physicians, were screened for delirium diagnosis. In addition, a newly introduced medication with haloperidol in patients without indication for a neuroleptic drug other than delirium was considered to indicate a physician diagnosed delirium. As suggested by Tse et al., the authors did not make any new retrospective diagnoses of delirium [26].

The primary endpoint, referred to as “delirium diagnosed by score,” was defined by a Nudesc ≥2 in at least one assessment. In our hospital, the Nudesc is routinely assessed by especially trained nurses in all patients on our ICU at least three times a day.

The motoric subtype of delirium was defined using the Richmond agitation and sedation scale (RASS), which is assessed at least three times daily in daily routine on our ICU [27]. According to literature, hyperactive delirium presumed when diagnosed in conjunction with RASS ≥1 and no RASS <0 in follow-up scores during delirium [28]. RASS scores <0 after necessary sedation due to agitation were excluded. Hypoactive delirium was presumed diagnosed in context of a RASS ≤0, whereas mixed delirium was defined as variable positive and negative RASS.

### Bias

Bias was reduced by predefining the primary endpoint “delirium”using either a well-established score or a detailed analysis of medical data. Group allocation was performed after data collection thereby reducing bias. Interpretation of variables was minimized and clear cutoff values were predefined. An adjustment for confounders was done by multivariable logistic regression analysis.

### Statistical methods

All relevant data is given in standardized tables. For data analysis, SPSS (version 26, IBM Statistics) and Prism (version 8, GraphPad) were employed. For statistical analysis, Mann-Whitney U-test was used for analysis of continuous variables. For categorical variables, Fisher’s exact test was used when number of expected values was smaller than five, otherwise Pearson’s Chi-squared test was performed. A p-value of <0.05 was considered statistically significant.

Three endpoints were further investigated by multivariable regression analysis. Firstly, risk factors for mortality were evaluated for interactions and in order to estimate the impact of the different delirium assessments using a binary regression analysis. Secondly, the duration of the ICU stay in all surviving patients was correlated with well-established risk factors for duration of ICU stay. Thirdly, risk factors of delirium were evaluated for interactions and in order to estimate the impact on delirium development using a binary regression analysis. We incorporated 8 prespecified well-established risk factors for delirium (age, psychiatric disease, dementia, alcohol abuse, mechanical ventilation, severe shock, acute kidney injury and SAPS2 score ≥ 50) which were tested to be significantly different in our dataset between patients with and without delirium (significance threshold p≤0.01 in either group) using a conditional forward selection process with a p-value threshold for entry of 0.2. Data are given as n (%), median and interquartile range (25th-75th) or odds ratio (OR) with 95% confidence interval (CI) if not stated otherwise.

## Results

### Baseline characteristics and delirium diagnosis

**Fig 1:**
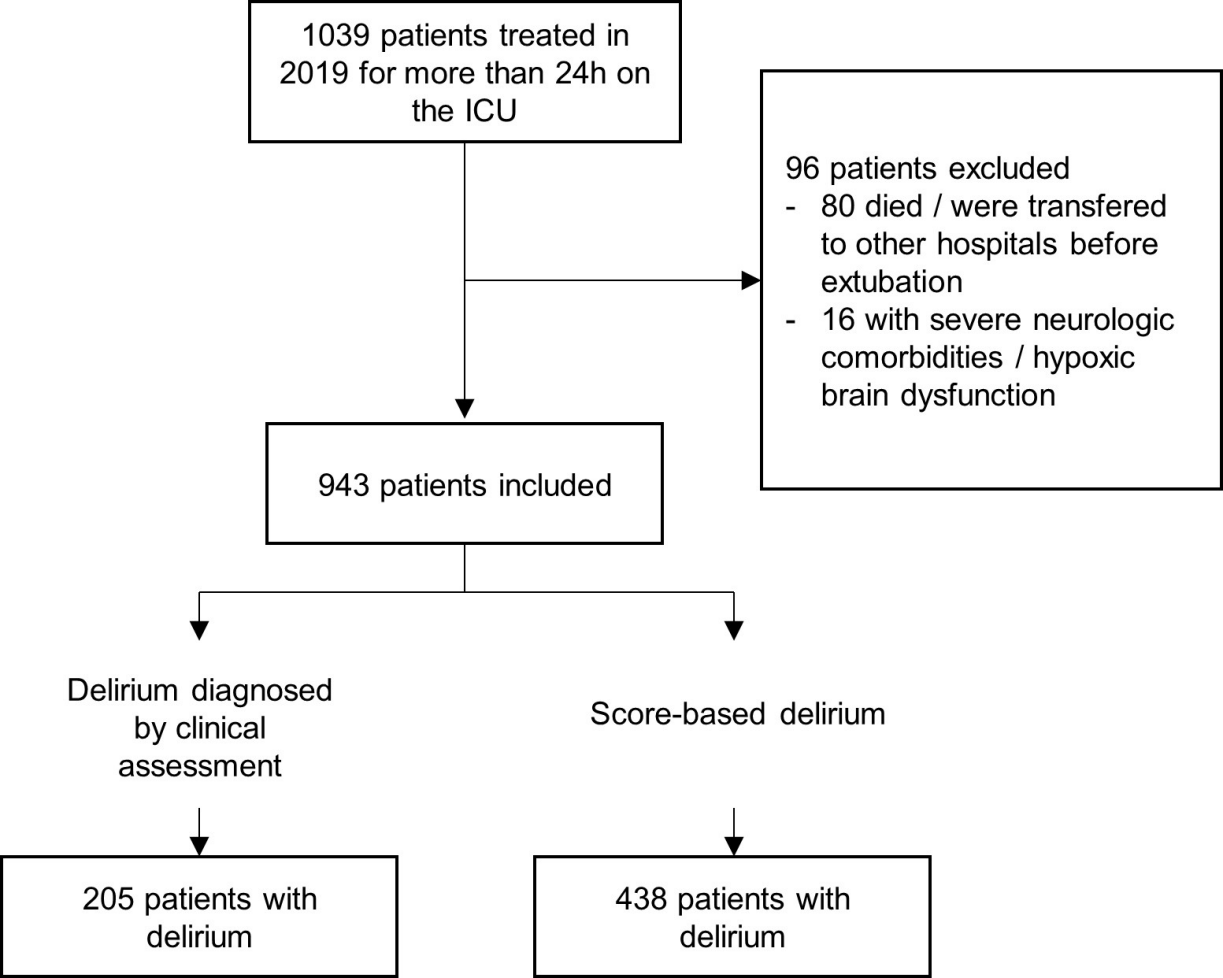
Flowchart indicating number of included and excluded patients. Data are given as number of patients.

In 2019, 1039 patients were treated on our medical intensive care unit for more than 24 hours. Of these, 96/1039 (9.0%) patients were excluded. 80 patients died or were transferred to other hospitals before extubation and 16 had severe neurologic comorbidities or hypoxic brain dysfunction, leading to 943 patients who could be assessed for delirium (Fig 1). Mean age was 70.2 (58.6–80.0) and 348 (36.9%) were female. Mechanical ventilation was necessary in 230/943 (24.4%). Most patients were admitted to the ICU for cardiac reasons (59.5%), followed by respiratory (17.4%), other (15.1%) and septic (13.5%) causes.

The primary endpoint of clinically diagnosed delirium occurred in 205/943 (21.7%) patients compared to 438/943 (46.4%; ratio 2.1) patients with delirium diagnosed by score. A total of 189/943 (20.0%) were diagnosed by both assessment methods. Of these 189 patients, 92.2% with clinical diagnosis were also diagnosed by Nudesc and 43.2% with diagnosis by Nudesc were detected clinically.

Patients with delirium in both groups (physician and score diagnosed) were older and had more comorbidities compared to those without delirium (Table 1). Therapy was more extensive in patients with delirium independent of the assessment method used (Table 2). For laboratory characteristics, see S1 Table.

**Table 1:** Baseline characteristics of all patients.

	Clinical diagnosis			Nudesc diagnosis		
	No delirium (N = 738)	Delirium (N = 205)	p-value	No delirium (N = 505)	Delirium (N = 438)	p-value
Age	69.7 (57.2–80.0)	74.0 (64.0–81.0)	**0.001**	69.7 (58.1–79.7)	71.1 (59.8–81.0)	0.052
Female	287 (38.9%)	61 (29.8%)	**0.017**	184 (36.4%)	164 (37.4)	0.749
Comorbidities						
Coronary heart disease	201 (27.2%)	66 (32.2%)	0.163	143 (28.3%)	124 (28.3%)	0.998
Heart rhythm disturbances	169 (22.9%)	74 (36.1%)	**<0.001**	109 (21.6%)	134 (30.6%)	**0.002**
Obesity	97 (13.1%)	27 (13.2%)	0.992	65 (12.9%)	59 (13.5%)	0.786
Pulmonary disease	137 (18.6%)	38 (18.5%)	0.993	92 (18.2%)	83 (18.9%)	0.773
Liver disease	53 (7.2%)	18 (8.8%)	0.443	28 (5.5%)	43 (9.8%)	**0.013**
Chronic kidney failure	129 (17.5%)	48 (23.4%)	0.054	87 (17.2%)	90 (20.5%)	0.193
Peripherial / cerebral arterial occlusive disease	67 (9.1%)	29 (14.1%)	**0.034**	47 (9.3%)	49 (11.2%)	0.341
Neurological disease	143 (19.4%)	50 (24.4%)	0.115	82 (16.2%)	111 (25.3%)	**0.001**
Malignancy	110 (14.9%)	26 (12.7%)	0.423	72 (14.3%)	64 (14.6%)	0.877
Psychiatric disease	72 (9.8%)	24 (11.7%)	0.414	28 (5.5%)	68 (15.5%)	**<0.001**
Dementia	19 (2.6%)	19 (9.3%)	**<0.001**	4 (0.8%)	34 (7.8%)	**<0.001**
Alcohol abuse	45 (6.1%)	19 (9.3%)	0.110	13 (2.6%)	51 (11.6%)	**<0.001**
Drug abuse	18 (2.4%)	8 (3.9%)	0.258	5 (1.0%)	21 (4.8%)	**<0.001**

p value reported in bold if difference is significant (p < 0.05). Data are given as median and interquartile range (25th-75th) or number of patients (percent of all patients in group).

**Table 2:** Clinical characteristics of all patients.

	Clinical diagnosis			Nudesc diagnosis		
	No delirium (N = 738)	Delirium (N = 205)	p-value	No delirium (N = 505)	Delirium (N = 438)	p-value
ICU stay (days)	1.9 (1.2–3.6)	5.7 (2.8–10.0)	**<0.001**	1.6 (1.2–2.6)	3.9 (2.0–8.1)	**<0.001**
Mortality	58 (7.9%)	27 (13.2%)	**0.019**	20 (4.0%)	65 (14.8%)	**<0.001**
ICU free days 15	13.1 (11.0–13.8)	8.9 (2.2–12.1)	**<0.001**	13.4 (12.2–13.8)	10.2 (3.3–12.9)	**<0.001**
TISS 10	5 (5–10); N = 725	10 (5–15); N = 202	**<0.001**	5 (3.8–10.0); N = 490	10 (5–15); N = 437	**<0.001**
SAPS 2	37 (28–48); N = 725	43 (34–53); N = 202	**<0.001**	35 (26–44); N = 490	44 (34–52); N = 437	**<0.001**
SAPS 2 ≥ 50	161 (22.2%)	65 (32.2%)	**0.004**	81 (16.5%)	134 (33.2%)	**<0.001**
Cause of illness						
cardiac	446 (60.4%)	115 (56.1%)	0.263	350 (69.3%)	211 (48.2%)	**<0.001**
respiratory	123 (16.7%)	41 (20.0%)	0.265	65 (12.9%)	99 (22.6%)	**<0.001**
infectious	96 (13.0%)	31 (15.1%)	0.433	51 (10.1%)	76 (17.4%)	**0.001**
other	110 (14.9%)	32 (15.6%)	0.803	53 (10.5%)	89 (20.3%)	**<0.001**
Resuscitation	33 (4.5%)	34 (16.6%)	**<0.001**	13 (2.6%)	54 (12.4%)	**<0.001**
Non-invasive ventilation	160 (21.7%)	87 (42.4%)	**<0.001**	81 (16.0%)	166 (37.9%)	**<0.001**
Mechanical ventilation	143 (19.4%)	87 (42.4%)	**<0.001**	73 (14.5%)	157 (35.7%)	**<0.001**
Days of invasive ventilation	0 (0–0.3)	0.4 (0–3.8)	**<0.001**	0 (0–0.1)	0.3 (0–2.7)	**<0.001**
Catecholamine therapy (shock)	233 (31.6%)	127 (62.0%)	**<0.001**	114 (22.6%)	246 (56.2%)	**<0.001**
Severe shock	95 (12.9%)	78 (38.0%)	**<0.001**	40 (7.9%)	133 (30.4%)	**<0.001**
ECMO	15 (2.0%)	16 (7.8%)	**<0.001**	4 (0.8%)	27 (6.2%)	**<0.001**
Coronary angiography	232 (31.4%)	70 (34.1%)	0.462	186 (36.8%)	116 (26.5%)	**0.001**
Acute kidney injury	272 (36.9%)	111(54.1%)	**<0.001**	153 (30.3%)	230 (52.5%)	**<0.001**
Renal replacement therapy	50 (6.8%)	26 (12.7%)	**0.006**	29 (5.7%)	47 (10.7%)	**0.005**
Arterial catheter	455 (61.7%)	174 (84.9%)	**<0.001**	275 (54.5%)	354 (80.8%)	**<0.001**
Central venous catheter	350 (47.4%)	126 (61.5%)	**<0.001**	207 (41.0%)	269 (61.4%)	**<0.001**
Urinal catheter	487 (66.0%)	185 (90.2%)	**<0.001**	281 (55.6%)	391 (89.3%)	**<0.001**
Necessity of blood transfusion	165 (22.4%)	71 (34.6%)	**<0.001**	90 (17.8%)	146 (33.3%)	**<0.001**

p value reported in bold if difference is significant (p < 0.05). Data are given as median and interquartile range (25th-75th) or number of patients (percent of all patients in group).

### Duration of ICU stay

**Fig 2:**
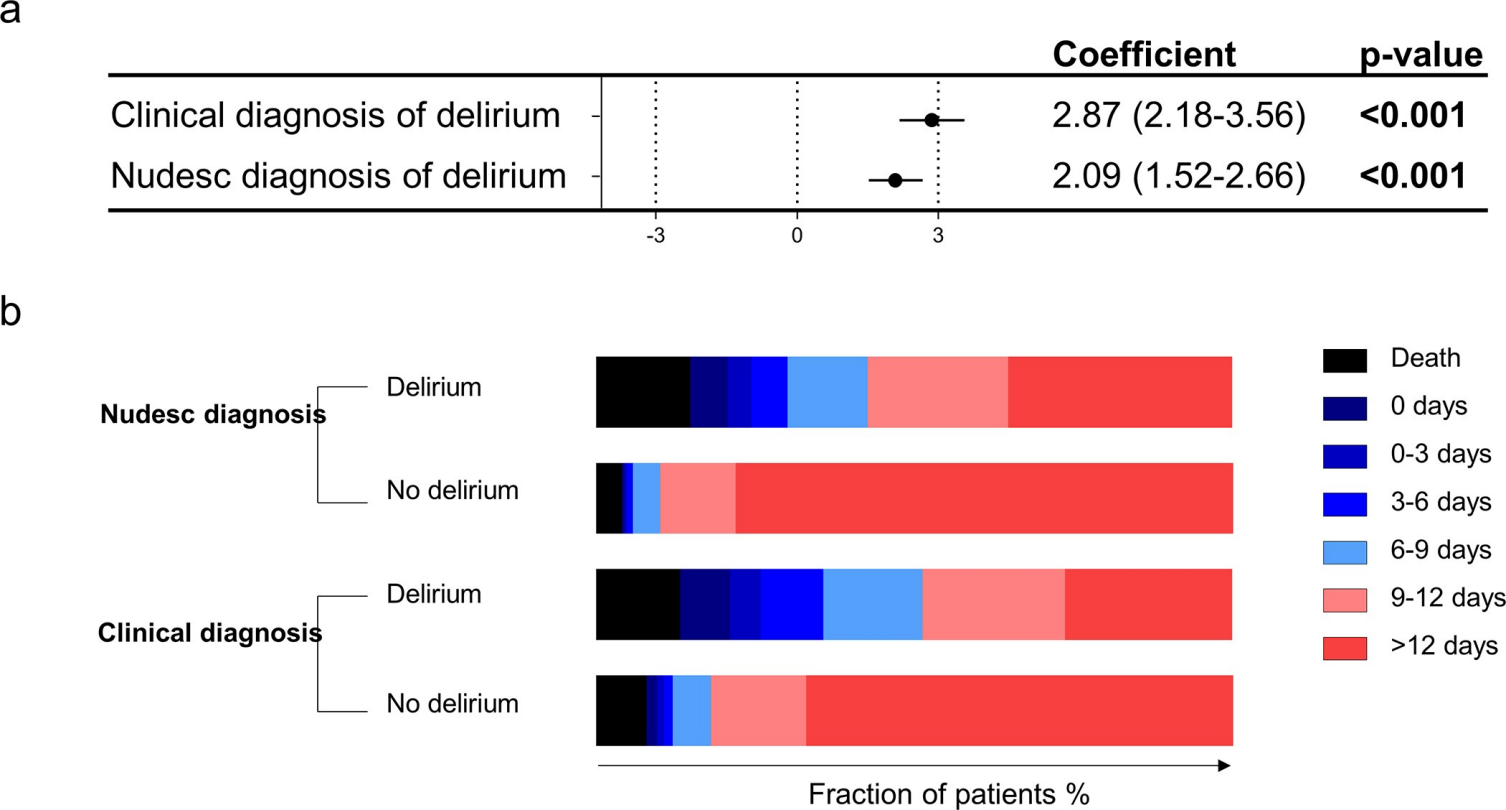
Duration of stay on the intensive care unit (ICU). Multivariable linear regression analysis showing regression coefficients for ICU stay (days) in all surviving patients adjusted for age, mechanical ventilation, severe shock, acute kidney and SAPS 2 ≥ 50 (a). ICU free days 15 days after ICU admission (b).

When considering the duration of ICU stay, both delirium endpoints were associated with a longer ICU stay. In a multivariable linear regression analysis including age, invasive ventilation, severe shock, acute kidney injury, and SAPS2 ≥ 50, both assessments were also independently associated with a longer duration of stay on the ICU in all surviving patients (Fig 2A). Patients with delirium, independent of the assessment method, had also significantly less ICU free days 15 days after ICU admission (Fig 2B).

### Association with mortality

**Fig 3:**
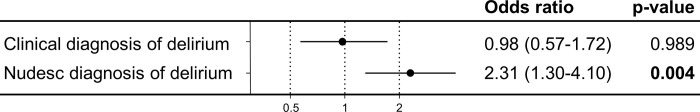
Multivariable binary logistic regression analyses for ICU mortality. Odds ratios adjusted for age, mechanical ventilation, severe shock, acute kidney injury and SAPS 2 ≥ 50.

In a multivariable binary analysis (using the same confounders as in the analysis above), the clinical diagnosis of delirium was not associated with an increased mortality (OR 0.98 (0.57–1.72); p = 0.989). In contrast, the score-based diagnosis of delirium was a strong and independent risk factors for ICU mortality in a multivariable binary analysis with the same confounders (OR 2.31 (1.30–4.10); p = 0.004) (Fig 3).

### Risk factors for delirium

**Fig 4:**
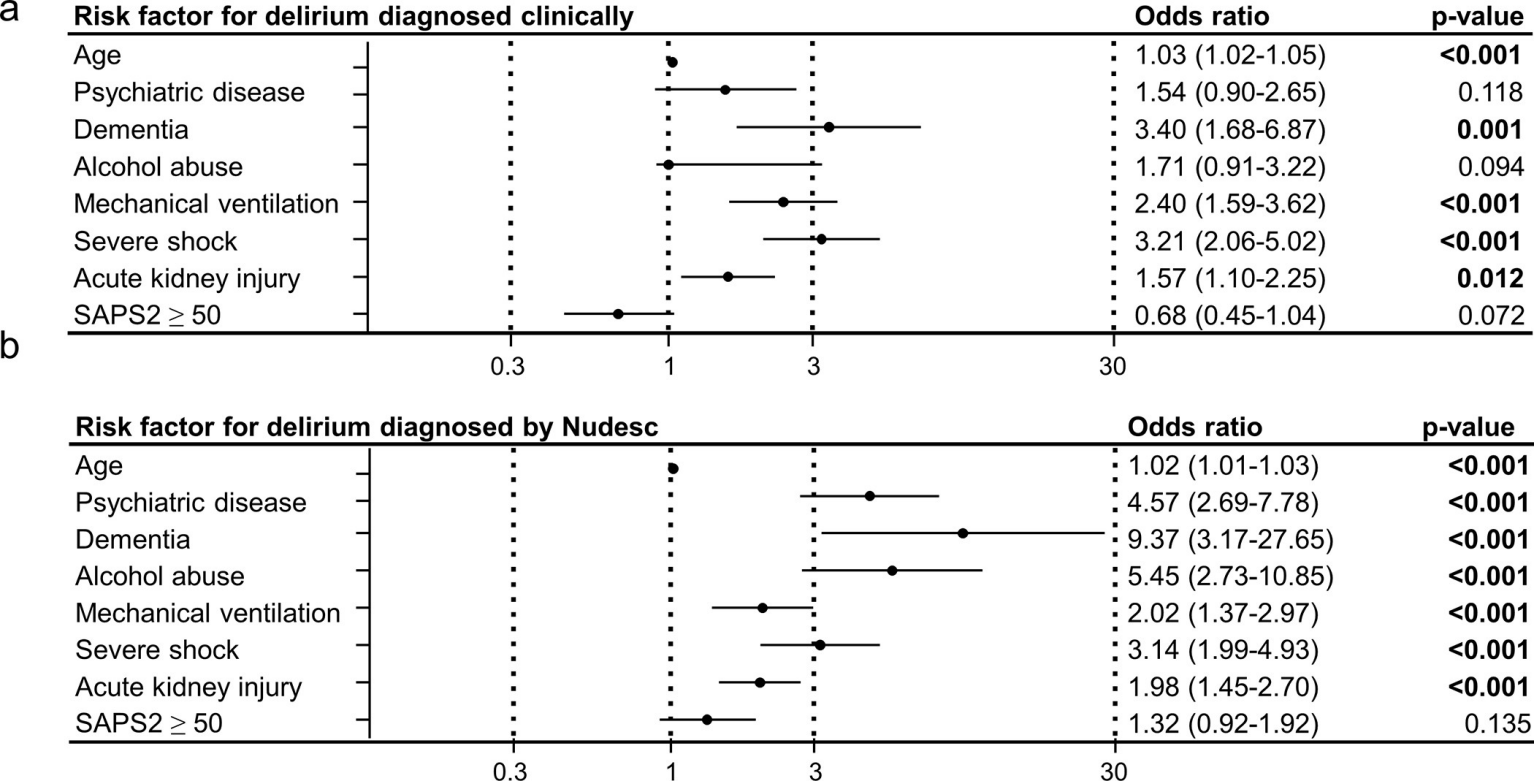
Risk factors for delirium. Figure shows multivariable logistic regression analysis with odds ratio (95% confidence interval) of different risk factors for delirium diagnosed by clinical diagnosis (a) and Nudesc (b). Odds ratios >1 mark positive predictors, odds ratio <1 negative predictors.

Multivariable binary regression analysis showed that age, dementia, invasive ventilation, severe shock and acute kidney injury were independent risk factors for a clinically diagnosed delirium whereas psychiatric diseases, alcohol abuse and a high SAPS2 at admission (SAPS2 ≥ 50) were not associated with a clinically diagnosed delirium (Fig 4A). Multivariable binary regression analysis showed that age, dementia, invasive ventilation, severe shock, acute kidney injury, psychiatric diseases, and alcohol abuse are independent risk factors for a score-based delirium diagnosis (Fig 4B).

### Delirium subtypes

**Fig 5:**
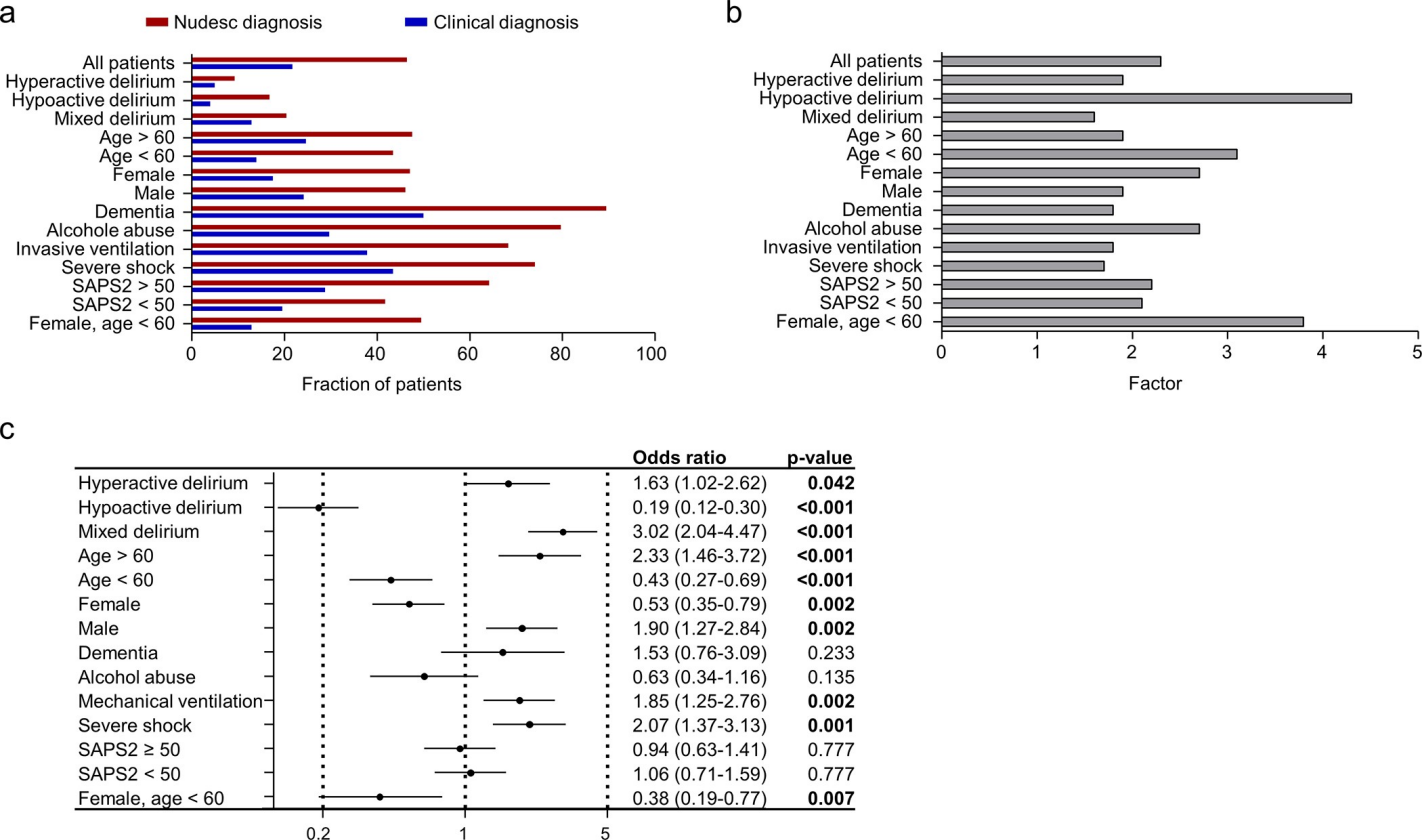
Fraction of delirium positive patients in different subgroups. (a). Factor of fraction of patients with delirium diagnosed by Nudesc divided by patients with clinical diagnosis of delirium (b). Univariate analysis in all Nudesc positive patients predicting a positive clinical diagnosis of delirium (c). Positive Odds ratios represent higher possibilities of a positive clinical diagnosis.

We further analyzed whether the clinical diagnosis and the score-based diagnosis vary in different subgroups. When diagnosed by physicians, hyperactive delirium occurred in 46/943 (4.9%), compared to 87/943 (9.2%) (ratio 1.9) in patients with a Nudesc diagnosis. Mixed delirium was the most often diagnosed form in both assessments (122/943 (12.9%) versus 193/943 (20.5%); ratio 1.6). Hypoactive delirium was the least clinically diagnosed form (37/943 (3.9%) versus 158/932 (16.8%); ratio 4.3). In all other subgroups analyzed, ratios of clinically diagnosed delirium and delirium diagnosed by score were close to the ratios for all patients, except for patients younger than 60 years, female patients and patients with alcohol abuse, who had higher ratios (3.1; 2.7; 2.7). Ratio of delirium in the subgroup of female patients younger than 60 was even higher (ratio 3.8) (Fig 5A and 5B). In a univariate analysis of all patients with delirium diagnosed by Nudesc, patients with hypoactive delirium, younger patients and female patients had significant negative odds ratios predicting a clinically diagnosed delirium (Fig 5C). Incidence of hypoactive delirium diagnosed by score was higher than in male patients, while no differences were seen when patients were divided by age (S2 Table).

## Discussion

In this single-center retrospective study, we found the score-based diagnosis of delirium (using the Nudesc score) detected delirium more often compared to a clinical diagnosis of delirium by ICU physicians. We found nearly a 2.1 fold increase in delirium detection in the Nudesc group compared to the clinical diagnosis group.

Diagnosis of delirium is complex and requires caution. While guidelines advocate a score based diagnosis of delirium, the National Institute for Health and Care Excellence rates the clinical diagnosis of delirium equally high [21,29]. Since no randomized, controlled studies comparing clinical assessment and screening tools for delirium are available level of evidence for delirium monitoring by score are low [29–33]. Data on surgical/trauma ICU patients suggesting no effect of daily screening for delirium with a screening tool on delirium-related outcomes including duration of delirium, mechanical ventilation, or ICU stay [30]. The authors hypothesize that an educational program of delirium may be just as effective as score based screening [30]. Similar finding are reported form a general ICU, where no changes in patient outcomes or diagnosis of delirium 1 year after implementation of a screening tool were detected [31]. Or data however suggests that clinical diagnosis of delirium underperforms in certain subgroups and a score based approach might be more appropriate on a medical ICU.

In literature, incidence of delirium is heterogeneous, depending on patient population and the assessment method used. On ICU, delirium is reported in 19–82% of patients [34]. Therefore, delirium rate reported here blends nicely into literature. The clinical significance of a delirium diagnosed by Nudesc is demonstrated by a strong association of delirium diagnosis ICU mortality (while physician diagnosis did not). This might underline the validity of the Nudesc diagnosis, since an association of delirium and mortality has been suggested in literature [8,34–36].

Furthermore, Nudesc ≥2 was an independent risk factor for a prolonged ICU stay, an association which has also been shown before for delirium [8,35,37]. Also, known independent risk factors of delirium, such as age, psychiatric diseases, dementia, alcohol abuse, severe shock and acute kidney injury, correlated with a Nudesc diagnosis of delirium which further emphasizes the validity [33–37].

Our subgroup analysis showed several interesting points. First, the rate of hypoactive delirium was particularly underdiagnosed in the clinical diagnosis group compared to the score-based group. This might be explained by the fact that quiet patients might be less noticed during the stressful daily routine of the ICU. Importantly, patients with hypoactive delirium might have the worst prognosis and consequently might be important to be identified [38].

Additionally, the ratios of delirium diagnosed by Nudesc were higher in the group of female patients and in patients younger than 60 years of age. With regard to female patients, it has been reported that delirium is diagnosed more frequently in men compared to women concerning all delirium subtypes [39]. Differences may partly be explained by a higher rate of hypoactive delirium in female patients but also because female patients may behave more inconspicuous than their male counterparts.

Concerning patient age, delirium might not be considered a plausible diagnosis in the young patient group. Although delirium incidence is increasing with age, delirium has been consistently reported in young patients and screening should also be performed in this subgroup.

### Limitations

When generalizing the results presented in the present study to other ICUs, several limitations have to be considered. Firstly, we report delirium diagnosis by ICU physicians. No independent verification of a definite delirium diagnosis was available for this research. A precise comparison of the clinical diagnosis and the diagnosis by Nudesc therefore lacks a definite diagnosis. Nevertheless, we show that a positive assessment with the Nudesc is independently predicted by variables already described for delirium and is also associated with mortality and ICU stay, which have also been described before for delirium. Since the Nudesc was recorded at the bedside during each shift and is available to ICU physicians, we cannot rule out that the result of the Nudesc influenced the clinical delirium diagnosis (especially in patients with Nudesc <2). Other screening tests, as the Confusion Assessment Method for the Intensive Care Unit (CAM-ICU), may have a higher sensitivity and specificity as the Nudesc on the ICU with better psychometric properties [16,18,40]. We present data of a general ICU with a heterogeneous patient collective. As incidence of delirium differs in different patient populations, our results cannot be generalized for special patient collectives. Clinical data was based on medical reports. Because we did not use structured clinical interviews, some variables like alcohol abuse may be underestimated. Due to the retrospective nature, minor complications on the ICU may have been underreported. Lastly, we report retrospective single-center data and results have to be considered hypothesis generating.

## Conclusion

Delirium is underdiagnosed by the treating physicians on our ICU. The score-based delirium diagnosis identifies patients with higher ICU mortality and ICU stay on a medical ICU. This particularly applies for hypoactive delirium, female patients and patients younger than 60 years. Special attention should be given to this patient collective when relying on a clinical definition of delirium. Our findings therefore advocate a score based approach rather than a clinical delirium diagnosis.

## Supporting information

S1 Table Laboratory characteristics of all patients. (DOCX)

S2 Table Minimal dataset. A minimal dataset of anonymous patient data used for this registry is available as S1 Table.
(PDF)

## Abbreviations

ICU: Intensive care unit
NuDesc: Nursing Delirium screening scale
RASS: Richmond agitation and sedation scale

## References

[1] American Psychiatric Association: Diagnostic and Statistical Manual of Mental Disorders. 5th edition. Arlington, VA: 2013.

[2] Ely EW, Inouye SK, Bernard GR, Gordon S, Francis J, May L, et al. Delirium in mechanically ventilated patients: validity and reliability of the confusion assessment method for the intensive care unit (CAM-ICU). JAMA. 2001; 286:2703–10. doi: 10.1001/jama.286.21.2703 . 11730446

[3] Lin S-M, Huang C-D, Liu C-Y, Lin H-C, Wang C-H, Huang P-Y, et al. Risk factors for the development of early-onset delirium and the subsequent clinical outcome in mechanically ventilated patients. J Crit Care. 2008; 23:372–9. doi: 10.1016/j.jcrc.2006.09.001 . 18725043

[4] Jäckel M, Bemtgen X, Wengenmayer T, Bode C, Biever PM, Staudacher DL. Is delirium a specific complication of viral acute respiratory distress syndrome. Crit Care. 2020; 24:401. Epub 2020/07/09. doi: 10.1186/s13054-020-03136-6 . 32646464 PMC7344036

[5] Jäckel M, Zotzmann V, Wengenmayer T, Duerschmied D, Biever PM, Spieler D, et al. Incidence and predictors of delirium on the intensive care unit after acute myocardial infarction, insight from a retrospective registry. Catheter Cardiovasc Interv. 2020. Epub 2020/09/14. doi: 10.1002/ccd.29275 . 32926556

[6] Jäckel M, Aicher N, Rilinger J, Bemtgen X, Widmeier E, Wengenmayer T, et al. Incidence and predictors of delirium on the intensive care unit in patients with acute kidney injury, insight from a retrospective registry. Sci Rep. 2021; 11:17260. Epub 2021/08/26. doi: 10.1038/s41598-021-96839-x . 34446816 PMC8390667

[7] Ali M, Cascella M. StatPearls. ICU Delirium. Treasure Island (FL); 2021. 32644706

[8] Ely EW, Siegel MD, Inouye SK. Delirium in the intensive care unit: an under-recognized syndrome of organ dysfunction. Semin Respir Crit Care Med. 2001; 22:115–26. doi: 10.1055/s-2001-13826 . 16088667

[9] Inouye SK, Bogardus ST, Charpentier PA, Leo-Summers L, Acampora D, Holford TR, et al. A multicomponent intervention to prevent delirium in hospitalized older patients. N Engl J Med. 1999; 340:669–76. doi: 10.1056/NEJM199903043400901 . 10053175

[10] van Eijk MMJ, van Marum RJ, Klijn IAM, Wit N de, Kesecioglu J, Slooter AJC. Comparison of delirium assessment tools in a mixed intensive care unit. Crit Care Med. 2009; 37:1881–5. doi: 10.1097/CCM.0b013e3181a00118 . 19384206

[11] Collins N, Blanchard MR, Tookman A, Sampson EL. Detection of delirium in the acute hospital. Age Ageing. 2010; 39:131–5. Epub 2009/11/16. doi: 10.1093/ageing/afp201 . 19917632

[12] Kishi Y, Kato M, Okuyama T, Hosaka T, Mikami K, Meller W, et al. Delirium: patient characteristics that predict a missed diagnosis at psychiatric consultation. Gen Hosp Psychiatry. 2007; 29:442–5. doi: 10.1016/j.genhosppsych.2007.05.006 . 17888812

[13] Inouye SK, Foreman MD, Mion LC, Katz KH, Cooney LM. Nurses’ recognition of delirium and its symptoms: comparison of nurse and researcher ratings. Arch Intern Med. 2001; 161:2467–73. doi: 10.1001/archinte.161.20.2467 . 11700159

[14] German Society of Anaesthesiology and Intensive Care Medicine (DGAI), German Interdisciplinary Association for Intensive Care and Emergency Medicine (DIVI). German S3 Guidelines (2015) "Analgesia, Sedation and management of delirium on the intensive care unit" [cited 17 Aug 2020]. Available from: https://www.awmf.org/leitlinien/detail/ll/001-012.html.

[15] Hargrave A, Bastiaens J, Bourgeois JA, Neuhaus J, Josephson SA, Chinn J, et al. Validation of a Nurse-Based Delirium-Screening Tool for Hospitalized Patients. Psychosomatics. 2017; 58:594–603. Epub 2017/07/24. doi: 10.1016/j.psym.2017.05.005 . 28750835 PMC5798858

[16] Gaudreau J-D, Gagnon P, Harel F, Tremblay A, Roy M-A. Fast, systematic, and continuous delirium assessment in hospitalized patients: the nursing delirium screening scale. J Pain Symptom Manage. 2005; 29:368–75. doi: 10.1016/j.jpainsymman.2004.07.009 . 15857740

[17] Bergjan M, Zilezinski M, Schwalbach T, Franke C, Erdur H, Audebert HJ, et al. Validation of two nurse-based screening tools for delirium in elderly patients in general medical wards. BMC Nurs. 2020; 19:72. Epub 2020/07/31. doi: 10.1186/s12912-020-00464-4 . 32760215 PMC7393733

[18] Luetz A, Heymann A, Radtke FM, Chenitir C, Neuhaus U, Nachtigall I, et al. Different assessment tools for intensive care unit delirium: which score to use. Crit Care Med. 2010; 38:409–18. doi: 10.1097/CCM.0b013e3181cabb42 . 20029345

[19] Neufeld KJ, Leoutsakos JS, Sieber FE, Joshi D, Wanamaker BL, Rios-Robles J, et al. Evaluation of two delirium screening tools for detecting post-operative delirium in the elderly. Br J Anaesth. 2013; 111:612–8. Epub 2013/05/08. doi: 10.1093/bja/aet167 . 23657522 PMC3770063

[20] Nacul FE, Paul N, Spies CD, Sechting H, Hecht T, Dullinger JS, et al. Influence of Sedation Level and Ventilation Status on the Diagnostic Validity of Delirium Screening Tools in the ICU-An International, Prospective, Bi-Center Observational Study (IDeAS). Medicina (Kaunas). 2020; 56. Epub 2020/08/13. doi: 10.3390/medicina56080411 . 32823781 PMC7466203

[21] National Institute for Health and Care Excellence. Delirium: prevention, diagnosis and management. 2019 [cited 07/2021]. Available from: https://www.nice.org.uk/guidance/cg103.

[22] Benchimol EI, Smeeth L, Guttmann A, Harron K, Hemkens LG, Moher D, et al. Das RECORD-Statement zum Berichten von Beobachtungsstudien, die routinemäßig gesammelte Gesundheitsdaten verwenden. Z Evid Fortbild Qual Gesundhwes. 2016; 115–116:33–48. Epub 2016/09/28. doi: 10.1016/j.zefq.2016.07.010 . 27837958 PMC5330542

[23] Section 2: AKI Definition. Kidney Int Suppl (2011). 2012; 2:19–36. doi: 10.1038/kisup.2011.32 . 25018918 PMC4089595

[24] Russell JA, Walley KR, Singer J, Gordon AC, Hébert PC, Cooper DJ, et al. Vasopressin versus norepinephrine infusion in patients with septic shock. N Engl J Med. 2008; 358:877–87. doi: 10.1056/NEJMoa067373 . 18305265

[25] Fong TG, Tulebaev SR, Inouye SK. Delirium in elderly adults: diagnosis, prevention and treatment. Nat Rev Neurol. 2009; 5:210–20. doi: 10.1038/nrneurol.2009.24 . 19347026 PMC3065676

[26] Tse L, Bowering JB, Schwarz SKW, Moore RL, Burns KD, Barr AM. Postoperative delirium following transcatheter aortic valve implantation: a historical cohort study. Can J Anaesth. 2015; 62:22–30. Epub 2014/10/22. doi: 10.1007/s12630-014-0254-2 . 25337965

[27] Sessler CN, Gosnell MS, Grap MJ, Brophy GM, O’Neal PV, Keane KA, et al. The Richmond Agitation-Sedation Scale: validity and reliability in adult intensive care unit patients. Am J Respir Crit Care Med. 2002; 166:1338–44. doi: 10.1164/rccm.2107138 . 12421743

[28] Peterson JF, Pun BT, Dittus RS, Thomason JWW, Jackson JC, Shintani AK, et al. Delirium and its motoric subtypes: a study of 614 critically ill patients. J Am Geriatr Soc. 2006; 54:479–84. doi: 10.1111/j.1532-5415.2005.00621.x . 16551316

[29] Devlin JW, Skrobik Y, Gélinas C, Needham DM, Slooter AJC, Pandharipande PP, et al. Clinical Practice Guidelines for the Prevention and Management of Pain, Agitation/Sedation, Delirium, Immobility, and Sleep Disruption in Adult Patients in the ICU. Crit Care Med. 2018; 46:e825–e873. doi: 10.1097/CCM.0000000000003299 . 30113379

[30] Bigatello LM, Amirfarzan H, Haghighi AK, Newhouse B, Del Rio JM, Allen K, et al. Effects of routine monitoring of delirium in a surgical/trauma intensive care unit. J Trauma Acute Care Surg. 2013; 74:876–83. doi: 10.1097/TA.0b013e31827e1b69 . 23425751

[31] Andrews L, Silva SG, Kaplan S, Zimbro K. Delirium monitoring and patient outcomes in a general intensive care unit. Am J Crit Care. 2015; 24:48–56. doi: 10.4037/ajcc2015740 . 25554554

[32] Reade MC, Eastwood GM, Peck L, Bellomo R, Baldwin I. Routine use of the Confusion Assessment Method for the Intensive Care Unit (CAM-ICU) by bedside nurses may underdiagnose delirium. Crit Care Resusc. 2011; 13:217–24. 22129282

[33] van den Boogaard M, Pickkers P, van der Hoeven H, Roodbol G, van Achterberg T, Schoonhoven L. Implementation of a delirium assessment tool in the ICU can influence haloperidol use. Crit Care. 2009; 13:R131. Epub 2009/08/10. doi: 10.1186/cc7991 . 19664260 PMC2750188

[34] Inouye SK, Westendorp RGJ, Saczynski JS. Delirium in elderly people. The Lancet. 2014; 383:911–22. doi: 10.1016/S0140-6736(13)60688-1 PMC4120864 23992774

[35] van den Boogaard M, Schoonhoven L, van der Hoeven JG, van Achterberg T, Pickkers P. Incidence and short-term consequences of delirium in critically ill patients: A prospective observational cohort study. Int J Nurs Stud. 2012; 49:775–83. Epub 2011/12/22. doi: 10.1016/j.ijnurstu.2011.11.016 . 22197051

[36] Veiga D, Luis C, Parente D, Fernandes V, Botelho M, Santos P, et al. Postoperative Delirium in Intensive Care Patients: Risk Factors and Outcome. Brazilian Journal of Anesthesiology. 2012; 62:469–83. doi: 10.1016/S0034-7094(12)70146-0 22793963

[37] Thomason JWW, Shintani A, Peterson JF, Pun BT, Jackson JC, Ely EW. Intensive care unit delirium is an independent predictor of longer hospital stay: a prospective analysis of 261 non-ventilated patients. Crit Care. 2005; 9:R375–81. Epub 2005/06/01. doi: 10.1186/cc3729 . 16137350 PMC1269454

[38] Robinson TN, Raeburn CD, Tran ZV, Brenner LA, Moss M. Motor subtypes of postoperative delirium in older adults. Arch Surg. 2011; 146:295–300. doi: 10.1001/archsurg.2011.14 . 21422360 PMC3346288

[39] Serpytis P, Navickas P, Navickas A, Serpytis R, Navickas G, Glaveckaite S. Age- and gender-related peculiarities of patients with delirium in the cardiac intensive care unit. Kardiol Pol. 2017; 75:1041–50. Epub 2017/07/17. doi: 10.5603/KP.a2017.0122 . 28715077

[40] Gélinas C, Bérubé M, Chevrier A, Pun BT, Ely EW, Skrobik Y, et al. Delirium Assessment Tools for Use in Critically Ill Adults: A Psychometric Analysis and Systematic Review. Crit Care Nurse. 2018; 38:38–49. doi: 10.4037/ccn2018633 . 29437077

