# Supplementary material for: Advantages of score-based delirium detection compared to a clinical delirium assessment—a retrospective, monocentric cohort study
Source: PLoS One. 2021 Nov 29;16(11):e0259841. doi: 10.1371/journal.pone.0259841 (PMC8629257; doi:10.1371/journal.pone.0259841)
Supplement: S1 Table — (DOCX) [file pone.0259841.s001.docx]

|  | **Clinical diagnosis** | |  |  | **Nudesc diagnosis** | |  |
| --- | --- | --- | --- | --- | --- | --- | --- |
|  |  |  |  |  |  |  |  |
|  | **No delirium (N=738)** | **Delirium (N=205)** | **p-value** |  | **No delirium (N=505)** | **Delirium (N=438)** | **p-value** |
| Bilirubin mg/dl | 1.1 (0.8-1.7); N=733 | 1.4 (0.9-2.1); N=204 | **<0.001** |  | 1 (0.7-1.5); N=501 | 1.4 (0.9-2.1); N=436 | **<0.001** |
| Hb minimum g/dl | 10.1 (7.7-12.2); N=733 | 8.8 (6.8-10.5); N=204 | **<0.001** |  | 10.6 (8.1-12.9); N=502 | 8.9 (7.0-10.7); N=435 | **<0.001** |
| Lactate mmol/l | 2.2 (1.5-3.4) N=733 | 2.9 (1.9-5.1); N=204 | **<0.001** |  | 2.0 (1.4-2.9); N=501 | 2.9 (1.9-4.7); N=436 | **<0.001** |
| pH minimum | 7.34 (7.25-7.39); N=733 | 7.25 (7.17-7.33); N=204 | **<0.001** |  | 7.35 (7.29-7.40); N=501 | 7.26 (7.18-7.34); N=436 | **<0.001** |
| CK U/l | 165 (72-628); N=594 | 354 (126-1081); N=184 | **<0.001** |  | 169 (72-530); N=404 | 251 (84-843); N=374 | **0.007** |
| CRP mg/dl | 63 (15-162); N=703 | 135 (72-236) | **<0.001** |  | 43 (10-118); N=475 | 129 (54-240); N=433 | **<0.001** |
| Creatinine at admission mg/dl | 1.13 (0.82-1.63) | 1.29 (0.88-2.14) | **0.007** |  | 1.08 (0.82-1.49) | 1.26 (0.86-2.07) | **<0.001** |
| Creatinine mg/dl | 1.21 (0.89-1.92) | 1.54 (1.02-2.92) | **<0.001** |  | 1.16 (0.88-1.66) | 1.46 (0.97-2.84) | **<0.001** |
| LDH U/l | 311 (230-507); N=564 | 389 (266-620); N=182 | **0.001** |  | 298 (227-460); N=366 | 382 (256-632); N=380 | **<0.001** |
| Leucocytes *10³/µl | 11.9 (8.4-16.6); N=733 | 15.6 (11.9-20.9) | **<0.001** |  | 11.1 (8.0-15.0); N=501 | 15.1 (11.1-20.4); N=437 | **<0.001** |
| Leucocytosis (>10^4/µl) | 461 (62.9%) | 179 (87.3%) | **<0.001** |  | 286 (57.1%) | 354 (81.0%) | **<0.001** |
| Procalcitonin ng/ml | 0.79 (0.22-4.93); N=316 | 1.14 (0.38-7.12); N=138 | 0.148 |  | 0.53 (0.16-3.87); N=179 | 1.21 (0.39-9.34); N=275 | **<0.001** |

Supplemental table 1 Laboratory characteristics of all patients

p value reported in bold if difference is significant (p < 0.05). Data are given as median and interquartile range (25th-75th) or number of patients (percent of all patients in group). Maximum values were analyzed if not stated otherwise.
