# Supplementary material for: Advantages of score-based delirium detection compared to a clinical delirium assessment—a retrospective, monocentric cohort study
Source: PLoS One. 2021 Nov 29;16(11):e0259841. doi: 10.1371/journal.pone.0259841 (PMC8629257; doi:10.1371/journal.pone.0259841)
Supplement: S2 Table — A minimal dataset of anonymous patient data used for this registry is available as S1 Table. (PDF) [file pone.0259841.s002.pdf]

sex	ICU stay (d)	ICU Free days 15 nomin	age	exclude	death	Heart rhythm disturbances	Coronary heart disease	Obesity	Pulmonary disease	Liver disease	Chronic kidney failure	Peripherial / cerebral arteri	Neurological disease	Malignancy	Psychiatric disease	Dementia	Alcohol abuse	Drug abuse	Non-invasive Ventilation	Invasive Ventilation	Catecholamine therapy	Noradrenalin	Noradrenalin 1-2mg/h	Noradrenalin >2mg/h	Dobutamin	Vasopressin/ Argipressin	Adrenalin	new RRT	chronic RRT	Transfusionen	Coronary angiography	TISS 10	SAPS 2	SAPS 2>50	Clinical diagnosis of deliriu	Nudesc diagnosis of deliriu	Hypoactive	Hyperactive	Mixed	AKI
m	3,8	12	31	0	0	0	0	0	0	0	0	0	0	1	0	0	0	0	0	0	1	1	0	0	0	0	0	0	0	1	0	9	51	1	0	0	1	0	0	1
w	4,0	12	61	0	0	0	0	0	1	0	0	0	0	0	1	0	0	0	1	0	0	0	0	0	0	0	0	0	0	0	0	10	29	0	0	1	0	0	1	0
w	2,1	15	88	0	0	1	0	0	0	0	0	0	0	1	0	0	0	0	0	0	1	1	0	0	0	0	0	0	0	0	0	5	51	1	0	1	1	0	0	0
m	4,2	12	60	0	0	0	0	0	0	0	0	0	0	0	0	0	0	0	1	0	0	0	0	0	0	0	0	0	0	0	0	10	50	1	0	0	0	0	0	0
m	3,0	12	69	0	0	1	0	0	0	1	0	0	0	0	0	0	0	0	0	0	0	0	0	0	0	0	0	0	0	0	0	5	60	1	0	0	0	0	0	0
m	2,2	15	83	0	0	0	0	0	0	0	0	0	1	0	0	0	0	0	1	0	0	0	0	0	0	0	0	0	0	0	1	5	51	1	0	1	0	1	0	0
m	14,1	3	54	0	0	1	0	0	0	0	1	0	0	0	0	0	0	0	0	0	1	1	0	0	1	0	1	0	0	1	1	9	26	0	0	0	0	0	0	1
m	1,1	15	68	0	0	1	1	0	1	0	0	0	1	1	0	0	0	0	0	0	0	0	0	0	0	0	0	0	0	0	1	5	20	0	0	0	0	0	0	0
m	3,1	12	70	0	0	0	0	0	1	0	0	0	0	0	0	0	1	0	1	0	1	1	0	0	0	0	0	0	0	0	0	14	38	0	0	0	0	0	0	1
m	3,1	12	61	0	0	1	0	0	0	0	1	0	0	0	0	0	0	0	1	0	0	0	0	0	0	0	0	0	0	0	1	10	40	0	1	1	0	0	1	0
m	14,7	3	64	0	0	0	0	0	0	0	0	0	1	0	0	0	0	0	1	1	1	1	1	0	0	0	0	0	0	0	0	20	45	0	0	1	1	0	0	1
m	1,9	15	75	0	0	0	0	0	1	0	0	0	0	0	0	0	0	0	0	1	1	0	0	0	0	0	0	0	0	1	0	10	48	0	0	0	0	0	0	0
m	16,3	0	81	0	0	0	0	0	0	1	0	0	0	0	0	0	0	0	0	1	1	1	0	1	0	0	0	0	0	1	0	10	54	1	1	1	1	0	0	1
m	19,6	0	43	0	0	0	0	0	0	0	0	0	0	1	0	0	0	1	1	1	1	1	0	1	0	0	0	1	0	1	0	15	45	0	0	1	1	0	0	1
m	17,3	0	68	0	0	1	1	0	1	1	0	0	0	0	1	0	0	0	0	1	1	1	0	1	0	1	1	1	0	1	1	23	64	1	1	1	0	0	1	1
m	51,4	0	64	0	0	1	0	0	0	0	0	0	0	0	0	0	0	0	1	1	1	1	0	1	1	0	1	0	0	1	1	24	65	1	1	1	0	0	1	1
m	6,2	9	57	0	0	0	0	0	0	0	0	0	0	1	0	0	0	0	1	0	1	1	0	0	0	0	0	0	0	1	0	15	38	0	0	0	0	0	0	0
m	1,7	15	71	0	0	0	0	0	0	0	0	0	0	0	0	0	0	0	0	0	0	0	0	0	0	0	0	0	0	0	0	14	29	0	0	0	0	0	0	0
w	5,2	12	63	0	0	0	0	0	0	0	1	0	0	0	0	0	0	0	1	0	1	1	0	0	0	0	0	0	0	0	0	10	46	0	0	1	0	1	0	0
m	1,7	15	64	0	0	1	0	0	0	1	0	0	0	0	0	0	0	0	0	1	1	1	1	0	0	0	0	0	0	1	0	19	63	1	0	0	0	0	0	0
w	10,9	6	78	0	0	1	1	0	0	0	1	0	0	0	0	0	0	0	0	0	1	1	0	0	0	0	0	0	0	0	0	5	31	0	1	1	1	0	0	1
m	1,2	15	80	0	0	1	1	1	0	0	0	0	0	0	0	0	0	0	0	0	0	0	0	0	0	0	0	0	0	0	0	5	26	0	0	0	0	0	0	0
w	1,1	15	83	0	0	0	0	0	1	0	1	0	0	0	0	0	0	0	0	0	0	0	0	0	0	0	0	0	0	0	1	5	37	0	0	0	0	0	0	0
m	1,9	15	50	0	0	0	0	0	0	1	0	0	0	0	0	0	0	0	0	0	0	0	0	0	0	0	0	0	0	1	0	5	48	0	0	1	1	0	0	1
m	1,4	15	53	0	0	1	0	1	0	1	0	0	0	1	0	0	0	0	0	0	0	0	0	0	0	0	0	0	0	0	1	5	22	0	0	0	1	0	0	0
m	1,2	15	85	0	0	0	0	0	1	0	0	0	1	0	0	0	0	0	0	0	0	0	0	0	0	0	0	0	0	0	1	5	40	0	0	0	1	0	0	0
w	5,9	12	84	0	0	1	0	0	1	0	0	0	0	0	0	1	0	0	1	0	0	0	0	0	0	0	0	0	0	0	0	10	43	0	0	1	0	0	1	0
m	3,1	12	60	0	0	0	0	0	1	0	0	0	0	1	0	0	0	0	0	0	0	0	0	0	0	0	0	0	0	0	0	5	39	0	0	0	0	0	0	0
w	3,8	12	67	0	0	0	0	1	1	0	1	0	0	0	0	0	0	0	1	0	0	0	0	0	0	0	0	0	0	0	0	10	53	1	0	0	0	0	0	1
m	2,1	15	84	0	0	0	0	0	0	0	0	0	0	0	0	1	0	0	0	0	0	0	0	0	0	0	0	0	0	0	0	5	43	0	1	0	0	0	1	0
w	12,1	3	52	0	0	0	0	0	1	0	0	0	0	0	0	0	0	0	1	1	1	1	1	0	0	0	1	0	0	1	1	24	53	1	0	0	1	0	0	1
m	1,2	15	52	0	0	0	0	0	0	0	0	0	0	1	0	0	0	0	0	0	0	0	0	0	0	0	0	0	0	1	0	10	38	0	0	1	1	0	0	1
m	7,0	9	67	0	0	0	1	0	0	0	0	0	0	0	0	0	1	0	0	1	1	1	0	0	0	0	0	0	0	1	1	20	38	0	0	1	0	0	1	0
w	2,0	15	84	0	0	1	1	0	1	0	1	0	0	0	0	0	0	0	1	0	0	0	0	0	0	0	0	1	1	0	0	3	50	1	0	1	1	0	0	0
m	8,1	9	74	0	0	0	0	0	0	0	0	0	0	0	0	0	0	0	1	1	1	1	0	0	0	0	0	0	0	0	0	10	36	0	1	1	0	0	1	1
m	2,1	15	73	0	0	0	0	0	0	0	0	0	1	0	0	0	0	0	0	0	1	1	0	0	0	0	0	0	0	1	0	5	65	1	0	0	0	0	0	1
m	1,0	15	80	0	0	1	1	0	0	0	1	1	0	0	0	0	0	0	0	0	0	0	0	0	0	0	0	0	0	0	0	5	39	0	0	0	0	0	0	0
m	1,6	15	52	0	0	0	0	0	0	0	0	0	0	1	0	0	0	0	0	0	0	0	0	0	0	0	0	0	0	1	0	0	46	0	0	0	0	0	0	0
w	4,0	12	57	0	0	0	0	0	0	0	0	0	0	1	0	0	0	0	0	0	0	0	0	0	0	0	0	0	0	0	0	5	28	0	0	0	0	0	0	0
w	4,4	12	76	0	0	0	0	0	0	0	0	0	0	1	0	0	0	0	1	0	0	0	0	0	0	0	0	0	0	1	0	5	43	0	1	1	0	0	1	0
w	1,5	15	72	0	0	0	0	0	0	0	0	0	0	1	0	0	0	0	0	0	1	1	0	0	0	0	0	0	0	0	0	10	63	1	0	0	0	0	0	0
w	1,9	15	67	0	0	0	0	0	0	0	1	0	0	1	0	0	0	0	0	0	0	0	0	0	0	0	0	0	0	1	0	10	35	0	0	1	0	1	0	0
w	1,3	15	66	0	0	0	0	0	0	0	1	0	0	1	0	0	0	0	0	0	0	0	0	0	0	0	0	0	0	1	0	10	36	0	0	0	1	0	0	1
m	11,1	6	56	0	0	1	0	0	0	0	0	0	0	0	0	0	0	0	1	0	1	1	1	0	0	0	0	0	0	1	0	27	33	0	1	1	0	1	0	0
m	6,0	9	82	0	0	1	1	0	1	0	0	1	0	0	0	0	0	0	1	0	0	0	0	0	0	0	0	0	0	0	1	5	50	1	0	0	0	1	0	0
m	29,9	0	67	0	0	0	1	1	0	0	0	0	1	1	0	1	0	0	1	1	1	1	1	0	0	0	0	1	0	1	0	10	56	1	1	1	0	0	1	1
m	2,9	15	44	0	0	0	0	0	0	0	0	0	0	1	0	0	0	0	0	1	0	0	0	0	0	0	0	0	0	1	0	15	49	0	0	0	0	0	0	1
m	1,1	15	78	0	0	0	0	0	0	1	0	0	0	1	0	0	0	0	0	0	1	1	0	0	0	0	0	0	0	0	0	10	69	1	0	0	1	0	0	0
w	2,8	15	76	0	0	0	0	0	0	0	0	0	0	0	0	0	0	0	0	0	0	0	0	0	0	0	0	0	0	0	1	0	45	0	0	0	0	0	0	1
m	1,5	15	85	0	0	0	0	0	0	0	0	0	0	1	0	0	0	0	0	0	1	1	0	0	0	0	0	0	0	0	0	10	54	1	1	1	0	0	1	0
w	4,1	12	82	0	0	0	0	0	0	0	1	0	0	0	0	0	0	0	0	0	1	1	0	1	0	1	0	0	0	1	0	18	59	1	0	1	1	0	0	1
m	2,3	15	61	0	0	0	1	0	0	0	0	0	0	0	0	0	0	0	0	1	1	0	0	0	0	0	0	0	0	0	0	10	38	0	0	0	0	0	0	0
w	1,2	15	53	0	0	1	0	0	0	0	0	0	1	1	1	0	0	0	0	0	0	0	0	0	0	0	0	0	0	1	0	10	42	0	0	0	0	0	0	0
w	2,4	15	80	0	0	0	1	0	0	0	0	0	0	0	0	0	0	0	0	0	0	0	0	0	0	0	0	0	0	0	1	5	52	1	0	0	0	0	0	1
m	6,1	9	88	0	0	1	1	0	0	0	1	0	0	0	0	0	0	0	0	1	0	0	0	0	0	0	0	0	0	0	0	0	35	0	1	1	0	0	1	0
w	2,1	15	81	0	0	1	0	1	0	0	1	0	1	0	0	1	0	0	0	0	1	1	0	0	0	0	0	0	0	0	0	5	39	0	0	1	1	0	0	1
m	7,1	9	64	0	0	0	0	0	0	0	1	0	0	0	0	0	0	0	1	0	0	0	0	0	0	0	0	1	0	1	0	10	72	1	0	0	0	0	0	1
m	1,4	15	83	0	0	1	0	0	0	0	0	0	0	1	0	0	0	0	0	0	0	0	0	0	0	0	0	0	0	0	0	0	55	1	0	0	1	0	0	1
m	5,4	12	81	0	0	1	0	0	0	0	0	0	0	0	0	0	0	0	1	1	1	1	0	0	0	1	1	0	0	0	1	10	62	1	1	1	0	1	0	1
w	1,1	15	77	0	0	1	0	1	0	0	1	0	0	1	0	0	0	0	0	0	0	0	0	0	0	0	0	0	0	0	0	5	53	1	0	0	0	0	0	1
m	2,1	15	59	0	0	0	1	0	0	1	0	0	1	1	0	0	0	1	1	0	1	1	0	1	0	1	0	0	0	1	0	18	63	1	0	1	1	0	0	0
w	3,2	12	81	0	0	0	0	0	0	0	0	1	0	0	0	0	0	0	0	0	1	1	0	0	0	0	0	0	0	1	0	14	51	1	0	1	1	0	0	0
m	1,1	15	78	0	0	0	1	0	0	0	0	0	0	0	0	0	0	0	0	0	0	0	0	0	0	0	0	0	0	0	1	5	29	0	0	0	0	0	0	0
m	3,4	12	83	0	0	1	0	0	0	1	1	0	0	0	0	0	0	0	0	0	1	1	0	0	0	0	0	0	0	1	0	10	44	0	0	1	1	0	0	1

m	1,1	15	84	0	0	0	1	0	0	0	0	0	1	0	0	0	0	0	0	1	1	1	0	0	0	0	0	0	0	0	1	19	60	1	0	0	0	0	0	0
w	3,9	12	85	0	0	1	1	1	0	0	1	1	1	0	0	0	0	0	0	1	1	1	0	0	0	0	0	0	0	1	0	10	56	1	1	1	1	0	0	0
m	3,6	12	83	0	0	0	0	0	1	0	0	0	0	0	0	0	0	0	1	0	1	1	0	0	0	0	0	0	0	0	0	10	45	0	0	1	1	0	0	1
m	13,7	3	69	0	0	0	1	0	0	0	0	0	0	0	0	1	0	0	0	1	1	1	1	0	0	0	0	0	0	1	1	10	43	0	1	1	0	0	1	0
m	2,9	15	82	0	0	1	0	0	0	1	1	1	1	0	0	0	1	0	0	0	1	1	0	0	0	0	0	0	0	0	1	5	53	1	0	1	1	0	0	1
m	1,1	15	37	0	0	0	0	0	0	0	0	0	1	1	0	0	0	0	0	0	0	0	0	0	0	0	0	0	0	0	0	5	24	0	0	0	0	0	0	0
m	7,4	9	64	0	0	0	0	0	0	0	0	0	1	1	0	0	0	0	0	0	0	0	0	0	0	0	0	0	0	1	0	5	30	0	0	0	1	0	0	0
w	2,4	15	89	0	0	1	1	0	1	0	0	1	0	0	0	0	0	0	0	0	0	0	0	0	0	0	0	0	0	0	0	5	35	0	0	0	0	0	1	0
w	2,4	15	80	0	0	1	0	0	0	0	1	0	1	1	0	0	0	0	0	0	0	0	0	0	0	0	0	1	1	0	0	8	46	0	0	0	0	0	0	0
m	2,7	15	74	0	0	0	1	0	1	0	1	1	0	1	0	0	0	0	1	0	1	0	0	0	1	0	0	0	0	1	0	15	36	0	0	0	0	0	0	1
m	1,9	15	42	0	0	0	0	0	0	1	1	0	0	0	0	0	0	0	0	0	0	0	0	0	0	0	0	1	0	1	0	8	21	0	0	1	1	0	0	1
m	6,9	9	67	0	0	0	0	0	0	0	0	0	0	1	0	0	0	0	0	0	0	0	0	0	0	0	0	1	0	1	0	9	50	1	0	0	0	0	0	1
m	10,4	6	72	0	0	0	0	0	0	0	0	0	0	1	0	0	0	0	1	1	1	1	1	0	0	0	0	0	0	1	0	14	48	0	0	1	0	0	1	1
m	5,8	12	73	0	0	0	1	0	1	0	0	1	0	0	0	0	1	0	0	1	0	1	1	0	0	0	0	0	0	0	1	19	59	1	0	1	1	0	0	1
w	3,2	12	69	0	0	0	0	0	1	1	1	0	0	0	0	0	0	0	0	0	0	0	0	0	0	0	0	0	0	0	0	5	37	0	0	0	1	0	0	1
m	1,0	15	69	0	0	0	1	0	0	0	0	0	0	0	0	0	0	0	0	0	0	0	0	0	0	0	0	0	0	0	1	0	31	0	0	0	0	0	0	1
w	4,8	12	57	0	0	1	0	0	1	0	0	0	0	0	0	0	0	0	1	0	1	1	0	0	0	0	0	0	0	0	0	24	49	0	0	0	0	0	0	0
m	3,8	12	91	0	0	0	0	0	1	0	0	0	0	0	0	1	0	0	1	0	1	1	1	0	0	0	0	0	0	0	0	10	63	1	0	1	1	0	0	1
m	1,0	15	77	0	0	0	1	0	0	0	0	0	0	0	0	0	0	0	0	0	0	0	0	0	0	0	0	0	0	0	0	0	29	0	0	0	0	0	0	0
w	1,0	15	66	0	0	0	0	0	0	0	0	0	0	1	0	0	0	0	0	1	0	0	0	0	0	0	0	0	0	0	0	10	49	0	1	1	1	0	0	0
m	1,7	15	70	0	0	0	0	0	0	0	0	0	0	0	0	0	0	0	0	0	0	0	0	0	0	0	0	0	0	0	0	0	30	0	0	1	1	0	0	0
m	1,1	15	72	0	0	0	0	0	0	0	0	0	0	0	0	0	0	0	0	0	0	0	0	0	0	0	0	0	0	0	1	0	39	0	0	0	0	0	0	1
m	7,4	9	82	0	0	1	0	0	0	0	0	0	0	0	0	0	0	0	1	1	1	1	0	1	0	0	0	1	0	1	0	23	66	1	1	1	0	0	1	1
w	10,0	6	58	0	0	0	0	0	1	0	1	0	1	0	1	0	0	0	0	0	0	0	0	0	0	0	0	1	1	1	0	3	23	0	0	1	1	0	0	0
m	1,6	15	82	0	0	1	0	1	0	0	0	0	0	1	0	0	0	0	0	0	0	0	0	0	0	0	0	1	0	1	0	3	42	0	0	0	0	0	0	1
m	3,8	12	58	0	0	0	0	0	1	0	0	0	0	1	0	0	0	0	0	0	0	0	0	0	0	0	0	0	0	0	0	5	41	0	0	0	0	0	0	1
m	2,2	15	62	0	0	0	0	0	0	0	1	0	0	0	0	0	0	0	0	0	0	0	0	0	0	0	0	0	0	0	0	9	32	0	0	1	0	1	0	1
m	5,6	12	86	0	0	1	1	0	0	0	1	0	0	1	0	0	0	0	0	0	1	1	0	0	0	0	0	0	0	1	0	5	43	0	0	1	0	0	1	1
m	1,1	15	78	0	0	0	1	1	0	0	0	0	0	0	0	0	0	0	0	0	0	0	0	0	0	0	0	0	0	0	1	5	47	0	0	0	0	0	0	1
m	1,9	15	76	0	0	1	1	0	0	0	0	0	0	0	0	0	0	0	0	0	1	1	0	0	0	0	0	0	0	0	0	4	43	0	0	0	0	0	0	1
m	5,0	12	67	0	0	0	1	0	0	0	1	0	0	1	0	0	0	0	0	0	1	1	1	0	0	0	0	0	0	1	0	10	40	0	1	1	0	1	0	1
w	7,9	100	49	0	1	0	0	0	0	0	0	0	0	0	0	0	0	0	1	1	1	1	0	1	1	1	1	1	0	1	1	31	59	1	1	1	0	1	0	1
m	11,7	100	68	0	1	1	0	0	0	0	0	0	0	1	0	0	0	0	1	1	1	1	0	1	0	1	0	0	0	1	0	5	50	1	0	1	0	0	1	1
w	6,3	100	62	0	1	0	0	0	0	0	0	0	0	1	0	0	0	0	1	1	1	1	0	0	0	0	0	0	0	0	0				0	0	0	0	0	0
m	15,7	100	62	0	1	0	0	0	0	0	0	0	0	0	0	0	0	0	0	1	1	1	0	1	0	0	1	1	0	1	0	32	62	1	0	1	1	0	0	1
w	1,2	100	37	0	1	0	0	0	0	0	0	0	0	1	0	0	0	0	1	0	1	1	0	0	0	0	0	0	0	0	0	10	50	1	0	1	0	0	1	0
w	3,5	100	79	0	1	1	0	0	0	0	0	0	0	0	0	0	0	0	1	0	1	1	0	0	1	0	0	0	0	0	0	14	42	0	0	1	0	0	1	1
m	19,4	100	80	0	1	1	0	0	0	0	0	0	1	0	0	0	0	0	1	0	1	1	1	0	1	0	0	0	0	1	1	10	51	1	1	1	0	0	1	0
w	4,1	100	80	0	1	0	0	0	0	0	0	0	0	1	0	0	0	0	0	0	0	0	0	0	0	0	0	0	0	0	0	10	65	1	0	0	0	0	0	1
m	2,2	100	63	0	1	0	0	0	0	0	0	0	1	1	0	0	0	0	0	0	0	0	0	0	0	0	0	0	0	0	0	5	53	1	0	1	0	0	1	1
w	4,2	100	66	0	1	0	0	0	0	1	0	0	0	0	0	0	1	0	0	0	1	1	0	0	0	0	0	0	0	1	0	10	47	0	0	1	0	0	1	1
m	7,5	100	62	0	1	0	1	0	1	0	1	1	0	0	0	0	1	0	1	1	1	1	0	0	0	0	0	0	0	0	0	15	46	0	1	1	0	1	0	1
w	7,0	100	77	0	1	1	0	0	0	0	0	0	1	0	0	0	0	0	1	0	1	1	0	0	0	0	0	0	0	0	0	10	45	0	0	1	0	0	1	1
w	2,5	100	73	0	1	0	0	0	1	0	0	0	1	0	0	0	0	0	1	0	1	1	1	0	0	0	0	0	0	0	1	15	43	0	1	0	0	0	1	1
w	1,9	100	61	0	1	0	0	1	0	0	0	1	0	0	0	0	0	0	0	0	1	1	0	0	1	0	0	0	0	0	1	14	47	0	0	1	1	0	0	1
w	7,9	100	80	0	1	1	1	1	0	0	0	0	0	0	0	0	0	0	0	1	1	1	0	0	1	0	0	0	0	0	1	31	55	1	0	1	0	0	1	1
m	4,7	100	61	0	1	0	0	0	0	0	0	0	0	1	0	0	0	0	1	0	0	0	0	0	0	0	0	0	0	0	0	5	36	0	0	0	0	0	0	0
w	10,7	100	21	0	1	0	0	0	0	0	0	0	0	1	0	0	0	0	1	1	1	1	0	1	0	0	0	1	0	1	0	15	57	1	0	1	0	0	1	1
m	15,0	100	55	0	1	0	0	0	0	0	0	0	0	1	0	0	0	0	1	0	0	0	0	0	0	0	0	0	0	0	0	10	52	1	0	1	0	0	1	0
m	7,2	100	41	0	1	0	0	0	0	1	0	0	0	0	0	0	0	0	1	1	1	1	0	1	0	1	0	1	0	1	0	0	47	0	1	1	0	0	1	1
m	2,4	100	67	0	1	0	1	0	1	0	0	0	1	0	0	0	0	0	1	1	1	1	1	0	0	0	0	0	0	0	0	10	55	1	0	1	0	0	1	0
w	2,0	100	92	0	1	0	0	0	0	0	0	0	1	0	0	0	0	0	0	0	0	0	0	0	0	0	0	0	0	0	1	5	49	0	0	1	0	1	0	1
w	11,0	100	38	0	1	0	0	0	0	0	0	0	0	0	0	0	0	0	1	1	1	1	0	0	0	0	0	0	0	1	0	15	24	0	1	1	0	0	1	0
m	3,9	100	58	0	1	0	0	0	0	1	0	0	0	0	0	0	0	0	0	0	1	1	0	0	0	0	0	0	0	1	0	14	32	0	0	1	0	1	0	1
m	18,9	100	86	0	1	1	1	0	1	0	1	0	0	0	0	0	0	0	1	1	1	0	0	1	1	1	1	0	0	1	1	18	72	1	1	1	0	0	1	1
w	5,8	100	68	0	1	0	0	0	0	0	0	0	0	1	0	0	0	0	0	0	1	1	1	0	0	0	0	0	0	1	0	4	72	1	0	1	1	0	0	1
m	2,1	100	76	0	1	1	1	0	0	0	1	1	1	0	0	0	0	0	0	0	1	1	1	0	1	0	0	0	0	0	1	9	68	1	1	1	0	0	1	1
w	1,6	100	76	0	1	1	0	0	0	0	1	1	1	0	0	0	0	0	0	0	1	0	0	0	0	0	0	0	0	1	1	5	54	1	0	0	0	0	0	0
w	2,3	100	80	0	1	1	0	0	0	0	0	0	0	0	0	0	0	0	0	0	1	1	1	0	1	0	0	0	0	0	1	26	71	1	1	1	0	0	1	1
m	2,9	100	54	0	1	0	0	0	0	1	0	0	0	0	0	0	0	0	1	1	1	1	0	1	0	0	1	1	0	1	0	18	64	1	0	0	0	0	0	1
m	3,3	100	90	0	1	0	1	0	0	0	0	0	1	0	0	0	0	0	1	0	1	1	0	0	0	0	0	0	0	0	0	5	40	0	0	1	0	0	1	0
w	5,5	100	55	0	1	0	0	0	0	0	0	0	0	1	0	0	0	0	0	1	1	1	1	0	0	0	0	0	0	1	0	5	37	0	0	0	0	0	0	1
m	7,2	100	70	0	1	0	0	0	0	1	1	0	1	0	0	0	0	0	1	0	1	1	0	0	0	0	0	1	1	1	0	15	73	1	0	1	0	0	1	0
w	1,1	100	84	0	1	0	0	0	0	0	1	0	1	0	0	0	0	0	0	1	1	1	1	0	1	0	0	0	0	0	1	24	73	1	0	0	0	0	0	1
m	1,1	100	92	0	1	1	1	0	0	0	0	1	1	0	0	0	0	0	0	0	0	0	0	0	0	0	0	0	0	0	1	5	27	0	0	0	0	1	0	0
w	4,8	100	71	0	1	0	0	0	1	1	0	0	0	1	0	0	0	0	1	0	1	1	1	0	0	0	0	0	0	1	0	10	45	0	0	1	0	0	1	0
m	6,6	100	71	0	1	0	0	0	0	0	1	0	0	1	0	0	0	0	0	0	1	1	0	0	0	0	0	1	1	0	0	5	57	1	1	0	0	0	1	0
w	8,3	100	63	0	1	0	0	1	0	0	0	0	0	1	0	0	0	0	0	0	1	1	0	0	0	0	0	0	0	0	0	5	47	0	0	0	1	0	0	1
w	6,9	100	69	0	1	0	0	0	1	0	1	0	1	0	1	0	0	0	0	0	1	1	1	0	0	1	0	0	0	1	0	10	60	1	0	1	0	0	1	1

w	4,4	100	79	0	1	1	0	0	0	0	0	0	1	0	0	0	0	0	0	0	1	1	0	1	0	0	0	0	0	1	0	9	58	1	0	1	1	0	0	1
m	19,4	100	77	0	1	0	1	0	0	0	0	0	0	0	0	0	1	0	0	1	1	1	0	1	0	1	0	0	0	1	0	27	57	1	1	1	0	0	1	1
m	11,7	100	76	0	1	0	1	0	0	0	1	1	1	0	0	0	0	0	1	1	1	1	0	0	0	0	0	1	0	0	1	15	50	1	1	1	0	0	1	1
m	2,0	100	70	0	1	0	0	0	0	0	0	0	1	0	0	1	0	0	1	0	1	1	0	0	0	0	0	0	0	1	0	0	41	0	0	1	1	0	0	1
w	3,8	100	82	0	1	0	0	0	1	0	0	1	1	0	0	0	0	0	1	0	0	0	0	0	0	0	0	0	0	0	0	10	51	1	1	1	0	1	0	1
w	8,0	100	85	0	1	0	0	0	0	0	0	1	1	0	0	0	0	0	1	1	1	1	0	0	0	0	0	0	0	0	0	14	31	0	0	1	0	0	1	0
w	10,5	100	81	0	1	0	0	0	1	0	0	0	0	0	0	0	0	0	1	1	1	1	0	0	0	0	0	0	0	0	0	10	48	0	1	1	0	0	1	0
w	30,1	100	64	0	1	1	0	0	0	0	0	0	0	0	0	0	0	0	1	1	0	0	0	0	0	0	0	0	0	1	0	10	36	0	1	0	0	0	1	1
w	8,4	100	79	0	1	1	0	0	0	0	0	0	1	0	0	0	0	0	0	0	0	0	0	0	0	0	0	0	0	0	0	0	44	0	0	1	1	0	0	1
w	1,2	100	78	0	1	0	0	0	1	0	0	0	0	0	1	0	0	0	1	0	1	1	0	0	0	0	0	0	0	0	0	15	46	0	0	1	0	0	1	0
w	5,6	100	56	0	1	0	0	0	0	0	0	0	0	1	0	0	0	0	1	1	0	0	0	0	0	0	0	0	0	1	0	10	43	0	0	0	0	0	0	1
m	16,2	100	79	0	1	1	0	0	0	0	0	0	1	0	0	0	0	0	0	1	1	1	1	0	0	0	0	1	0	1	0	25	77	1	0	1	0	0	1	1
w	47,7	100	40	0	1	0	0	1	0	0	1	0	0	0	1	0	0	0	1	1	1	1	1	0	0	0	0	0	0	1	0	19	39	0	0	1	0	0	1	1
m	9,7	100	85	0	1	1	0	0	0	0	1	1	1	0	0	0	0	0	1	1	1	1	1	0	0	0	0	1	0	1	1	15	55	1	1	1	0	0	1	1
m	25,0	100	54	0	1	0	0	0	0	1	0	0	0	0	0	0	0	1	1	1	1	1	0	1	0	0	0	1	0	1	0	19	48	0	0	1	0	0	1	1
m	5,6	100	80	0	1	1	1	0	0	0	0	1	0	0	0	0	0	0	0	0	1	1	1	0	1	0	0	1	0	0	1	18	55	1	0	1	1	0	0	1
m	5,3	100	60	0	1	0	0	0	0	1	0	1	0	0	0	0	1	0	0	0	1	1	1	0	0	0	0	0	0	1	0	14	58	1	0	1	0	1	0	1
m	12,6	100	70	0	1	1	1	0	0	0	1	1	1	0	0	0	0	0	0	0	1	1	0	0	0	0	0	1	1	0	1	3	51	1	1	1	0	0	1	0
m	2,6	100	80	0	1	0	0	0	0	0	0	1	0	0	0	0	0	0	0	0	0	0	0	0	0	0	0	0	0	1	0	9	51	1	0	1	1	0	0	1
m	1,2	100	90	0	1	1	0	0	0	1	1	0	0	0	0	0	0	0	1	0	1	1	0	1	1	0	0	1	0	0	1	26	91	1	0	0	1	0	0	1
w	6,3	100	85	0	1	1	1	0	0	0	1	0	0	0	0	0	0	0	1	0	1	1	0	0	0	0	0	1	0	1	0	13	67	1	1	1	0	0	1	1
m	19,1	100	81	0	1	1	1	0	1	0	0	1	0	0	0	0	0	0	1	0	1	1	1	0	0	0	0	0	0	0	0	0	42	0	1	1	0	1	0	1
m	23,9	100	71	0	1	0	0	0	1	0	0	0	0	1	0	0	0	0	0	1	1	1	0	1	0	0	0	1	0	1	0	10	60	1	1	1	0	1	0	1
m	9,1	100	67	0	1	0	0	0	1	0	0	0	0	0	0	0	0	0	1	0	0	0	0	0	0	0	0	0	0	1	0	10	53	1	1	1	1	0	0	0
m	3,6	100	59	0	1	0	0	0	0	0	0	0	0	0	0	0	0	0	1	1	0	0	0	0	0	0	0	0	0	0	0	5	43	0	0	0	0	0	0	1
m	4,8	100	76	0	1	0	0	0	0	0	1	0	0	1	1	0	0	0	1	0	1	1	0	0	0	0	0	0	0	0	0	9	88	1	0	1	0	0	1	1
w	10,9	100	68	0	1	0	0	1	0	0	0	0	0	1	0	0	0	0	1	1	1	1	1	0	0	0	0	0	0	1	0	15	56	1	0	0	0	0	0	0
w	1,2	100	74	0	1	1	0	0	0	0	0	1	0	1	0	0	0	0	1	0	0	0	0	0	0	0	0	0	0	1	0	10	62	1	0	1	1	0	0	1
w	1,2	100	81	0	1	1	0	0	0	0	0	0	0	0	1	1	1	0	0	0	0	0	0	0	0	0	0	0	0	0	0	0	54	1	0	0	0	0	0	0
m	19,8	100	74	0	1	1	0	0	0	1	0	0	0	0	0	0	0	0	1	0	1	1	0	0	0	1	1	0	0	1	0	14	36	0	1	1	0	0	1	1
m	21,8	100	81	0	1	1	1	0	0	0	0	0	0	0	1	1	0	0	1	1	1	1	0	0	0	0	0	0	0	1	1	19	52	1	1	1	0	0	1	1
m	18,3	100	74	0	1	0	0	0	1	0	0	0	0	0	0	0	0	0	1	1	1	1	0	1	0	0	1	0	0	1	0	23	53	1	1	1	0	0	1	1
m	1,9	100	77	0	1	1	1	1	1	0	0	0	0	1	0	0	0	0	0	0	1	1	0	0	0	1	1	0	0	1	0	10	44	0	0	1	0	0	1	1
m	8,4	100	79	0	1	0	0	0	0	0	1	0	0	0	0	1	0	0	1	1	1	0	1	0	0	0	0	0	0	0	1	15	63	1	1	1	0	0	1	0
m	2,4	100	72	0	1	0	0	1	1	0	0	0	0	1	0	0	0	0	1	0	1	1	0	0	0	0	0	0	0	0	0	18	52	1	0	1	1	0	0	0
w	1,5	100	80	0	1	0	0	0	0	0	1	0	0	0	1	0	0	0	0	1	1	1	1	0	0	0	0	0	0	1	0	14	52	1	0	0	1	0	0	1
m	19,7	100	71	0	1	1	0	0	0	1	0	1	0	0	0	0	0	0	1	1	1	1	0	0	0	0	0	0	0	1	0	10	58	1	0	1	0	0	1	1
m	3,3	100	83	0	1	1	1	0	0	0	1	0	0	0	0	0	0	0	1	0	1	1	0	0	0	0	0	0	0	1	0	15	61	1	0	1	1	0	0	1
m	25,0	100	54	0	1	0	0	0	0	1	0	0	0	0	0	0	1	1	1	1	1	1	0	0	0	0	0	1	0	1	0	15	57	1	0	1	0	0	1	1
m	1,9	100	86	0	1	1	0	0	0	0	0	0	0	0	0	0	0	0	1	0	1	1	0	0	0	0	0	0	0	0	1	15	55	1	0	1	0	0	1	1
m	10,2	100	70	0	1	1	0	0	0	0	0	0	0	1	1	0	0	0	1	0	1	1	0	1	0	0	0	1	0	1	0	5	72	1	1	1	0	0	1	1
m	2,6	100	76	0	1	1	1	0	0	0	1	0	1	0	1	0	0	0	0	0	1	1	0	1	0	0	0	0	0	0	0	18	59	1	0	1	1	0	0	1
m	9,7	100	74	0	1	0	1	0	0	0	0	0	0	0	0	0	0	0	1	1	1	1	1	0	0	0	0	0	0	1	0	23	42	0	1	1	1	0	0	1
m	4,2	100	58	0	1	0	0	0	0	1	0	0	0	1	0	0	0	0	1	1	1	1	0	1	0	0	0	1	0	1	0	23	36	0	0	0	0	0	0	1
m	7,1	100	80	0	1	1	1	1	0	0	1	1	0	0	0	0	0	0	1	1	1	1	0	1	1	1	1	0	0	1	1	19	75	1	0	1	1	0	0	1
w	22,8	100	55	0	1	1	0	0	0	0	0	0	1	0	0	0	0	0	0	1	1	1	1	0	1	0	1	1	0	1	1	21	42	0	0	1	0	0	1	1
w	1,2	100	76	0	1	0	0	0	1	0	0	0	0	1	1	0	0	0	0	0	1	1	0	0	0	0	0	0	0	1	0	9	60	1	0	0	0	0	0	0
m	2,4	15	49	0	0	0	0	0	0	0	0	0	0	0	0	0	0	0	0	0	0	0	0	0	0	0	0	0	0	0	1	5	17	0	0	0	1	0	0	0
m	4,0	12	61	0	0	0	0	0	0	0	0	0	0	0	0	0	0	0	0	0	0	0	0	0	0	0	0	0	0	0	1	0	27	0	0	0	1	0	0	0
m	1,0	15	84	0	0	0	1	0	1	0	0	0	1	0	0	0	0	0	0	0	0	0	0	0	0	0	0	0	0	0	0	5	37	0	0	0	1	0	0	0
w	9,1	6	67	0	0	0	0	0	0	0	0	0	0	1	0	0	0	0	0	1	1	0	1	0	0	0	0	0	0	1	0	24	40	0	1	1	0	0	1	0
w	1,1	15	65	0	0	0	0	0	1	0	0	0	0	0	0	0	0	0	1	0	0	0	0	0	0	0	0	0	0	0	0	10	38	0	0	1	1	0	0	0
m	2,0	15	63	0	0	0	0	0	0	0	0	0	0	0	0	0	0	0	0	0	0	0	0	0	0	0	0	0	0	0	1	5	26	0	0	0	1	0	0	0
w	4,8	12	56	0	0	0	0	0	0	1	0	0	0	0	1	0	0	0	0	1	1	1	1	0	0	0	0	0	0	0	0	19	31	0	1	1	0	0	1	0
m	1,0	15	71	0	0	0	0	0	0	0	0	0	0	0	0	0	0	0	0	0	0	0	0	0	0	0	0	0	0	0	1	0	25	0	0	0	1	0	0	0
m	9,1	6	42	0	0	0	0	0	0	1	0	0	0	0	0	0	1	0	0	0	1	1	0	0	0	0	0	0	0	0	0	13	53	1	1	0	0	0	1	1
m	4,0	12	86	0	0	0	1	1	0	0	0	0	0	0	0	0	0	0	1	0	1	1	0	0	1	0	1	0	0	0	0	9	31	0	0	0	1	0	0	1
m	3,1	12	69	0	0	0	0	0	1	0	0	1	0	0	0	0	0	0	0	0	0	0	0	0	0	0	0	0	0	0	1	10	37	0	0	0	1	0	0	0
m	1,9	15	62	0	0	0	0	0	0	0	0	0	0	0	0	0	0	0	0	0	0	0	0	0	0	0	0	0	0	0	1	0	33	0	0	0	1	0	0	0
m	1,0	15	24	0	0	0	0	0	0	0	0	0	0	0	0	0	0	0	0	0	0	0	0	0	0	0	0	0	0	0	1	0	10	0	0	0	1	0	0	0
m	2,6	15	69	0	0	0	0	0	0	0	0	0	0	0	0	0	0	0	0	0	1	1	0	0	0	0	0	0	0	1	0	19	37	0	0	0	0	1	0	1
m	2,0	15	68	0	0	0	0	0	0	0	0	0	0	0	0	0	0	0	0	0	0	0	0	0	1	0	0	0	0	0	0	5	33	0	0	0	1	0	0	1
w	3,5	12	32	0	0	0	0	0	1	0	0	0	1	0	0	0	0	0	1	0	0	0	0	0	0	0	0	0	0	0	0	0	20	0	0	0	1	0	0	0
m	5,7	12	56	0	0	1	0	0	0	0	0	0	0	0	0	0	0	0	0	1	1	1	0	0	1	0	0	0	0	0	1	14	41	0	0	1	0	1	0	0
w	3,8	12	80	0	0	0	0	0	0	0	0	0	0	0	0	0	0	0	1	0	1	1	0	0	0	0	0	0	0	0	0	15	59	1	0	0	1	0	0	0
m	2,2	15	77	0	0	0	1	0	0	0	0	0	0	0	0	1	0	0	0	0	0	0	0	0	0	0	0	0	0	0	0	5	29	0	1	1	0	0	1	0
m	3,5	12	39	0	0	0	0	0	0	0	1	0	0	0	1	0	0	0	0	0	0	0	0	0	0	0	0	1	1	0	1	17	24	0	0	1	1	0	0	0
m	2,0	15	92	0	0	1	1	0	1	0	0	0	1	0	0	1	0	0	0	0	0	0	0	0	0	0	0	0	0	0	0	5	52	1	0	1	0	0	1	1
m	11,9	6	57	0	0	0	0	1	0	0	0	0	0	0	0	0	0	0	1	1	1	1	0	1	0	0	0	0	0	1	1	15	45	0	0	1	0	1	0	1

w	8,5	9	40	0	0	0	0	0	0	0	0	0	0	0	0	0	1	0	0	0	0	0	0	0	0	0	0	0	0	0	0	0	30	0	0	1	0	1	0	0
w	10,3	6	62	0	0	0	0	0	0	0	0	0	0	0	1	0	0	0	1	1	1	1	1	0	0	0	0	0	0	1	1	15	43	0	1	1	0	1	0	0
m	1,4	15	56	0	0	0	0	0	1	0	0	0	0	0	0	0	0	0	0	0	0	0	0	0	0	0	0	0	0	0	1	5	24	0	0	0	1	0	0	0
m	1,9	15	78	0	0	0	0	0	0	0	0	0	0	0	0	0	0	0	0	0	0	0	0	0	0	0	0	0	0	0	0	5	29	0	0	1	1	0	0	0
m	16,7	0	67	0	0	0	0	0	0	0	0	0	0	0	0	0	0	0	1	1	1	0	1	0	0	0	0	0	0	0	1	15	48	0	1	1	0	0	1	1
w	3,7	12	64	0	0	1	0	0	0	0	0	0	0	0	0	0	0	0	0	0	0	0	0	0	0	0	0	0	0	0	1	0	29	0	0	0	1	0	0	0
w	3,1	12	40	0	0	0	0	0	0	0	0	0	0	0	1	0	0	0	0	1	0	0	0	0	0	0	0	0	0	0	0	15	27	0	0	1	1	0	0	0
w	7,1	9	25	0	0	0	0	0	0	0	0	0	0	0	0	0	0	0	1	1	0	0	0	0	0	0	0	0	0	0	0	15	33	0	1	1	0	1	0	1
m	30,7	0	80	0	0	1	1	0	0	0	0	0	0	0	0	0	1	0	1	1	1	1	0	0	0	0	1	0	0	1	1	19	55	1	1	1	0	0	1	0
w	1,8	15	87	0	0	0	0	0	0	0	0	0	0	0	0	0	0	0	0	0	0	0	0	0	0	0	0	0	0	0	1	0	37	0	0	0	1	0	0	0
m	1,2	15	78	0	0	0	1	0	0	0	0	0	0	0	0	0	0	0	0	0	0	0	0	0	0	0	0	0	0	0	0	5	24	0	0	0	1	0	0	0
w	1,3	15	61	0	0	0	0	0	0	0	0	0	0	0	0	0	0	0	0	0	0	0	0	0	0	0	0	0	0	0	1	0	25	0	0	0	0	0	0	0
m	1,0	15	76	0	0	0	0	1	0	0	0	0	0	0	0	0	0	0	0	0	0	0	0	0	0	0	0	0	0	0	0	5	18	0	0	0	0	0	0	0
m	2,3	15	57	0	0	0	1	0	0	0	1	1	0	0	0	0	0	0	1	0	0	0	0	0	0	0	0	0	0	0	1	10	40	0	0	0	1	0	0	0
w	26,2	0	20	0	0	0	0	0	0	1	0	0	1	0	1	0	0	0	0	0	0	0	0	0	0	0	0	0	0	1	0	10	19	0	1	1	0	0	1	0
m	5,8	12	60	0	0	0	0	0	0	0	0	0	0	0	1	0	1	0	0	1	0	0	0	0	0	0	0	0	0	0	0	10	28	0	1	1	0	0	1	0
m	2,8	15	60	0	0	0	0	0	0	0	1	0	0	0	0	0	0	0	0	0	0	0	0	0	0	0	0	0	0	0	1	0	21	0	0	0	1	0	0	0
w	1,4	15	79	0	0	0	0	0	0	0	0	1	0	0	0	0	0	0	0	0	0	0	0	0	0	0	0	0	0	0	0	0	24	0	0	0	1	0	0	0
w	1,2	15	82	0	0	0	0	0	0	0	0	0	1	0	0	0	0	0	0	0	0	0	0	0	0	0	0	0	0	0	0	10	42	0	0	0	0	0	0	0
m	1,1	15	79	0	0	1	1	1	1	0	1	0	0	0	1	0	0	0	0	0	0	0	0	0	0	0	0	0	0	0	0	5	24	0	0	0	0	0	0	0
m	1,3	15	31	0	0	0	0	0	0	0	0	0	0	0	0	0	1	0	0	1	1	1	0	0	0	0	0	0	0	0	0	15	28	0	0	0	0	0	0	0
m	1,9	15	67	0	0	0	0	1	0	0	1	0	0	0	0	0	0	0	0	0	1	1	0	0	0	0	0	0	0	0	0	5	28	0	0	1	1	0	0	0
m	1,0	15	62	0	0	0	0	0	0	0	0	0	1	0	0	0	0	1	0	1	0	0	0	0	0	0	0	0	0	0	0	10	37	0	0	0	0	0	0	0
m	1,2	15	68	0	0	1	0	0	0	0	1	0	0	0	0	0	0	0	0	0	0	0	0	0	0	0	1	0	0	0	0	5	36	0	0	0	0	0	0	0
w	1,8	15	81	0	0	0	0	0	0	0	0	0	0	0	0	0	0	0	0	0	1	1	0	0	0	0	0	0	0	0	0	5	34	0	0	1	1	0	0	0
m	6,9	9	66	0	0	0	0	0	0	0	0	0	1	0	0	0	0	0	1	0	0	0	0	0	0	0	0	0	0	0	0	5	23	0	0	0	1	0	0	0
w	6,8	9	73	0	0	1	0	0	0	0	1	0	0	0	0	0	0	0	1	0	0	0	0	0	0	0	0	0	0	0	0	5	36	0	0	0	0	0	0	0
m	1,1	15	81	0	0	0	0	0	0	0	0	0	0	0	0	0	0	0	0	0	0	0	0	0	0	0	0	0	0	0	1	5	36	0	0	0	1	0	0	0
m	2,9	15	76	0	0	1	0	0	1	0	0	0	0	1	0	0	0	0	1	0	1	1	0	0	0	0	0	0	0	0	0	10	48	0	1	1	0	0	1	1
m	1,9	15	67	0	0	0	1	0	1	0	1	1	0	0	0	0	0	0	0	0	1	1	0	0	0	0	0	0	0	0	0	5	19	0	0	0	0	0	0	0
w	1,4	15	84	0	0	0	0	0	0	0	0	0	0	1	0	0	0	0	0	0	0	0	0	0	0	0	0	0	0	0	1	0	39	0	0	0	0	0	0	0
w	5,0	12	75	0	0	0	0	0	0	0	0	0	0	0	1	0	0	0	0	0	0	0	0	0	0	0	0	1	0	0	0	3	41	0	1	1	0	0	1	1
w	1,6	15	58	0	0	0	0	0	0	1	0	0	0	0	0	0	0	0	0	0	0	0	0	0	0	0	0	0	0	0	0	5	43	0	0	1	0	0	1	0
m	1,5	15	62	0	0	0	1	0	0	0	0	0	1	0	0	0	0	0	0	0	0	0	0	0	0	0	0	0	0	0	1	10	31	0	0	0	0	0	0	0
m	1,0	15	60	0	0	0	0	0	0	0	0	0	0	0	0	0	0	0	0	0	0	0	0	0	0	0	0	0	0	0	1	5	20	0	0	0	0	0	0	1
m	10,4	6	39	0	0	0	0	0	0	0	0	0	0	0	0	0	1	1	1	1	1	1	0	1	0	1	1	1	0	1	0	19	60	1	0	0	0	0	0	1
m	1,0	15	81	0	0	1	1	0	0	0	0	0	1	0	0	0	0	0	0	1	1	1	0	0	0	0	0	0	0	0	0	15	37	0	0	0	0	0	0	0
m	16,8	0	72	0	0	1	0	0	0	0	0	0	1	0	0	0	0	0	1	0	1	1	0	0	0	0	0	0	0	0	0	10	49	0	0	1	1	0	0	1
m	1,2	15	56	0	0	0	0	1	0	0	1	0	1	0	0	0	0	0	0	0	0	0	0	0	0	0	0	0	0	0	0	5	27	0	0	0	0	0	0	1
w	1,3	15	71	0	0	0	0	1	0	0	0	0	0	0	0	0	0	0	0	0	0	0	0	0	0	0	0	0	0	0	0	5	39	0	0	0	0	0	0	0
m	1,2	15	84	0	0	0	0	0	0	0	0	0	0	0	0	0	0	0	0	0	0	0	0	0	0	0	0	0	0	0	1	0	44	0	0	0	0	0	0	0
W	2,6	15	36	0	0	0	0	0	0	0	0	0	0	0	0	0	0	0	0	1	1	1	0	0	0	0	0	0	0	0	0	15	32	0	0	0	0	0	0	0
m	15,0	0	53	0	0	0	0	0	0	0	0	0	0	0	0	0	0	0	1	1	1	1	0	0	1	0	0	0	0	1	1	19	53	1	0	1	0	0	1	0
w	3,1	12	69	0	0	0	0	0	0	0	0	1	1	0	1	0	0	0	1	0	0	0	0	0	0	0	0	0	0	0	0	10	48	0	0	1	1	0	0	0
m	1,0	15	84	0	0	1	1	0	0	1	1	1	1	0	0	0	0	0	0	1	1	1	0	0	0	0	0	0	0	0	0	15	45	0	0	0	0	0	0	0
m	3,9	12	78	0	0	0	0	0	0	0	0	1	1	0	0	0	0	0	0	0	0	0	0	0	0	0	0	0	0	0	0	5	27	0	1	1	1	0	0	0
m	1,4	15	82	0	0	0	0	0	0	0	0	0	0	0	0	0	0	0	0	0	0	0	0	0	0	0	0	0	0	0	0	0	39	0	0	0	0	0	0	0
m	16,2	0	52	0	0	0	0	0	0	0	0	0	1	0	0	0	0	0	1	1	1	1	0	0	0	0	0	0	0	0	1	15	53	1	0	0	0	0	0	0
m	2,1	15	76	0	0	0	1	0	0	0	1	0	0	0	0	1	0	0	0	0	0	0	0	0	0	0	0	0	0	1	0	5	78	1	0	1	1	0	0	1
w	1,1	15	84	0	0	0	1	1	0	0	0	0	0	0	0	0	0	0	0	0	0	0	0	0	0	0	0	0	0	0	0	5	37	0	0	0	0	0	0	0
w	8,3	9	39	0	0	0	0	0	1	0	0	0	0	0	0	0	0	0	0	1	1	0	1	0	0	0	0	0	0	0	1	10	32	0	0	1	0	0	1	0
w	3,4	12	56	0	0	0	0	0	0	0	0	0	1	1	1	0	0	0	0	0	0	0	0	0	0	0	0	0	0	0	1	0	25	0	0	1	1	0	0	0
w	1,9	15	57	0	0	0	0	0	0	0	0	0	0	1	0	0	0	0	1	1	1	1	1	0	0	0	0	0	0	0	0	10	43	0	0	1	1	0	0	0
w	1,1	15	81	0	0	1	1	0	0	0	1	0	0	0	0	0	0	0	0	0	0	0	0	0	0	0	0	0	0	0	0	0	25	0	0	0	0	0	0	0
m	16,2	0	58	0	0	0	0	0	0	0	0	0	0	0	0	0	0	0	1	1	1	0	1	0	0	0	1	0	0	1	1	19	48	0	1	1	0	0	1	1
m	2,5	15	65	0	0	0	0	0	0	0	0	0	0	1	0	0	0	0	0	1	0	0	0	0	0	0	0	1	0	1	1	17	66	1	0	0	0	0	0	1
w	1,7	15	58	0	0	0	0	1	0	0	0	0	0	0	0	0	0	0	0	0	0	0	0	0	0	0	0	0	0	0	0	0	27	0	0	0	0	0	0	1
m	3,7	12	68	0	0	0	1	0	1	0	0	1	1	0	0	0	0	0	0	1	1	1	1	0	1	0	0	0	0	0	0	22	54	1	0	1	0	1	0	1
w	1,2	15	83	0	0	0	1	0	1	0	1	0	0	0	0	0	0	0	0	0	0	0	0	0	0	0	0	0	0	0	0	5	34	0	0	0	0	0	0	0
m	5,1	12	51	0	0	0	0	0	1	0	0	0	0	0	0	0	0	0	0	1	1	1	0	0	1	0	0	0	0	0	0	23	49	0	1	1	0	0	1	1
m	6,4	9	80	0	0	0	0	0	0	0	1	0	0	0	1	0	0	0	0	0	0	0	0	0	0	0	0	0	0	1	0	8	29	0	1	1	0	1	0	1
w	1,0	15	82	0	0	1	0	0	0	0	0	0	1	0	0	1	0	0	0	0	0	0	0	0	0	0	0	0	0	0	0	10	42	0	0	1	1	0	0	0
w	7,6	9	84	0	0	0	0	0	1	0	0	0	0	0	0	1	0	0	0	1	1	1	1	0	0	0	0	0	0	0	0	10	54	1	0	1	0	0	1	0
m	6,9	9	33	0	0	0	0	0	0	0	0	0	0	0	1	0	0	0	0	0	0	0	0	0	1	0	0	0	0	0	1	14	15	0	0	1	1	0	0	0
m	2,6	15	38	0	0	0	0	0	0	0	0	0	0	1	0	0	0	0	0	0	0	0	0	0	0	0	0	0	0	1	0	4	6	0	0	1	0	1	0	0
w	8,8	9	89	0	0	0	0	0	0	0	1	0	0	0	0	0	0	0	1	1	1	1	0	0	0	0	0	0	0	0	0	15	44	0	0	1	0	1	0	1
w	1,0	15	84	0	0	0	1	0	1	0	1	0	1	0	1	0	0	0	0	0	0	0	0	0	0	0	0	0	0	0	0	5	35	0	0	1	1	0	0	0
w	1,2	15	82	0	0	1	1	0	0	0	0	0	0	0	0	0	0	0	0	0	1	1	0	0	0	0	0	0	0	1	0	18	45	0	0	0	0	0	0	1
m	3,0	12	83	0	0	1	0	0	0	0	0	0	0	0	0	0	0	0	0	1	1	1	0	0	0	0	0	0	0	0	1	15	52	1	1	1	1	0	0	1

w	4,2	12	29	0	0	0	0	0	0	0	0	0	0	0	0	0	0	0	1	0	0	0	0	0	0	0	0	1	1	1	0	8	25	0	0	0	0	0	0	0
m	2,0	15	83	0	0	0	0	0	0	0	0	1	1	0	0	0	0	0	0	0	0	0	0	0	0	0	0	0	0	0	1	5	33	0	1	1	0	0	1	0
m	8,7	9	70	0	0	0	0	0	0	0	1	0	0	0	0	0	0	0	0	1	1	1	0	1	0	0	0	0	0	0	1	18	63	1	1	1	1	0	0	1
m	2,8	15	78	0	0	1	0	0	0	0	0	0	0	0	0	0	0	0	0	0	0	0	0	0	0	0	0	0	0	1	1	0	33	0	0	0	0	0	0	0
m	2,1	15	50	0	0	0	0	0	0	1	0	0	0	0	0	0	1	0	0	0	0	0	0	0	0	0	0	0	0	1	0	5	27	0	1	1	0	1	0	1
w	1,4	15	68	0	0	0	0	0	0	0	0	0	0	0	0	0	0	0	0	0	0	0	0	0	0	0	0	0	0	0	0	0	18	0	0	0	0	0	0	0
m	1,1	15	15	0	0	0	0	0	0	0	0	0	0	1	0	0	0	0	0	0	0	0	0	0	0	0	0	1	0	0	0	3	29	0	0	0	0	0	0	1
m	1,3	15	81	0	0	0	0	1	1	0	1	0	0	0	0	0	0	0	0	0	0	0	0	0	0	0	0	0	0	0	1	0	26	0	0	0	0	0	0	0
m	1,3	15	98	0	0	0	0	0	0	0	0	0	0	0	0	0	0	0	0	0	0	0	0	0	0	0	0	0	0	1	0	0	52	1	0	1	0	1	0	1
w	1,1	15	90	0	0	0	1	0	0	0	0	0	0	0	0	0	0	0	0	0	0	0	0	0	0	0	0	0	0	0	0	5	28	0	1	1	1	0	0	0
m	1,1	15	83	0	0	0	1	0	0	0	0	0	1	0	1	0	0	0	0	0	0	0	0	0	0	0	0	0	0	0	0	5	30	0	0	0	0	0	0	0
m	1,8	15	90	0	0	0	1	0	0	0	0	0	0	0	0	0	0	0	0	0	0	0	0	0	0	0	0	1	1	0	1	8	51	1	1	1	0	1	0	0
w	7,3	9	71	0	0	0	0	0	0	0	0	0	0	0	0	0	0	0	1	1	1	1	1	0	0	0	0	1	0	1	0	18	70	1	0	0	0	0	0	1
m	3,7	12	77	0	0	0	0	0	0	0	0	0	0	0	0	0	0	0	0	0	1	1	0	0	0	0	0	0	0	1	0	10	37	0	0	0	0	0	0	0
w	6,9	9	89	0	0	1	0	0	0	0	1	0	0	0	0	0	0	0	1	0	1	1	1	0	0	0	0	0	0	0	0	9	40	0	1	1	0	0	1	1
m	1,6	15	88	0	0	0	1	0	1	0	0	0	0	0	0	0	0	0	0	0	0	0	0	0	0	0	0	0	0	0	1	5	30	0	0	0	1	0	0	0
m	3,5	12	34	0	0	0	0	0	0	0	0	0	0	0	0	0	0	1	0	0	1	1	0	0	0	0	0	0	0	0	0	0	21	0	1	1	1	0	0	0
m	1,2	15	87	0	0	0	1	0	0	0	0	1	0	0	0	0	0	0	0	1	1	1	0	0	0	0	0	0	0	0	1	10	49	0	0	0	0	0	0	0
w	1,3	15	39	0	0	0	0	0	0	0	0	0	0	0	1	0	0	0	0	0	0	0	0	0	0	0	0	0	0	0	0	0	12	0	0	0	0	0	0	0
m	1,7	15	73	0	0	0	1	0	0	0	0	0	0	0	0	0	0	0	0	0	0	0	0	0	0	0	0	0	0	0	0	10	28	0	0	0	0	0	0	0
w	4,1	12	29	0	0	0	0	0	0	0	0	0	0	0	0	0	0	0	0	1	1	1	0	0	0	0	0	0	0	0	0	18	36	0	0	1	1	0	0	0
w	3,1	12	93	0	0	0	1	0	0	0	0	0	0	0	0	0	0	0	0	0	1	1	0	0	0	0	0	0	0	0	1	5	29	0	1	1	0	1	0	0
m	3,1	12	67	0	0	0	1	1	0	0	0	0	0	0	0	0	0	0	0	1	1	1	0	0	0	0	0	0	0	0	1	10	44	0	0	0	0	0	0	1
m	9,0	6	62	0	0	0	0	0	0	0	0	0	0	0	0	0	0	0	0	1	1	1	0	0	0	0	0	0	0	0	0	27	42	0	1	1	0	0	1	1
w	1,3	15	78	0	0	0	0	0	0	0	0	0	0	0	0	0	0	0	1	0	0	0	0	0	0	0	0	0	0	0	1	15	45	0	0	0	0	0	0	0
w	1,2	15	62	0	0	0	0	0	0	0	0	0	0	0	0	0	0	0	0	0	0	0	0	0	0	0	0	0	0	1	0	10	34	0	0	0	0	0	0	0
m	6,0	9	49	0	0	0	0	1	0	0	0	0	0	0	0	0	0	0	1	1	1	1	0	0	0	0	0	0	0	0	0	10	40	0	1	1	0	0	1	1
m	1,2	15	27	0	0	0	0	0	0	0	0	0	0	0	0	0	0	0	0	0	0	0	0	0	0	0	0	0	0	0	0				0	0	0	0	0	1
m	14,2	3	64	0	0	0	0	0	0	0	0	0	0	0	0	0	0	0	1	1	1	1	0	0	0	0	0	0	0	0	0	15	46	0	0	1	1	0	0	0
m	1,4	15	79	0	0	0	0	0	0	0	0	0	0	0	0	0	0	0	0	0	0	0	0	0	0	0	0	0	0	0	1	5	50	1	0	0	0	0	0	1
m	3,3	12	66	0	0	0	1	0	0	0	1	0	0	0	0	0	0	0	0	1	1	1	0	0	0	0	0	1	1	0	0	17	55	1	0	0	1	0	0	0
m	1,9	15	72	0	0	1	0	0	1	0	0	0	0	0	0	0	0	0	0	0	1	1	0	0	0	0	1	0	0	0	0	9	44	0	0	0	0	0	0	1
m	1,5	15	78	0	0	0	0	0	0	0	0	0	0	0	0	0	0	0	0	0	0	0	0	0	0	0	0	0	0	0	1	5	35	0	0	0	0	0	0	0
m	2,3	15	59	0	0	0	0	0	0	0	0	0	1	0	0	0	0	0	0	0	0	0	0	0	0	0	0	0	0	0	1	0	18	0	0	0	0	0	0	0
w	1,1	15	91	0	0	0	0	0	0	0	0	0	0	0	0	0	0	0	0	0	0	0	0	0	0	0	0	0	0	0	0	5	27	0	1	1	0	1	0	0
w	1,3	15	64	0	0	0	0	0	0	0	1	0	0	0	0	0	0	0	0	0	0	0	0	0	0	0	0	0	0	0	0	0	57	1	0	0	0	0	0	1
w	2,1	15	60	0	0	0	0	0	0	0	0	0	0	0	1	0	0	0	0	1	0	0	0	0	0	0	0	0	0	0	0	10	31	0	1	0	0	0	1	0
w	1,3	15	68	0	0	0	0	1	0	0	0	0	0	0	0	0	0	0	0	0	0	0	0	0	0	0	0	0	0	0	0	5	34	0	0	0	1	0	0	0
w	1,5	15	48	0	0	0	0	0	1	1	0	0	0	0	1	0	1	0	0	1	0	0	0	0	0	0	0	0	0	1	0	10	31	0	0	0	0	0	0	0
w	6,2	9	61	0	0	0	0	0	0	0	0	0	0	0	0	0	0	0	1	1	1	1	0	0	0	0	0	1	0	1	0	27	58	1	0	0	1	0	0	1
m	1,0	15	74	0	0	0	1	1	0	0	0	0	0	0	0	0	0	0	0	0	0	0	0	0	0	0	0	0	0	0	1	5	28	0	0	0	0	0	0	0
m	2,5	15	85	0	0	0	1	0	0	0	1	0	0	0	0	0	0	0	0	0	0	0	0	0	0	0	0	0	0	0	1	5	40	0	0	1	1	0	0	1
w	4,1	12	72	0	0	0	0	0	0	0	0	1	0	0	0	0	0	0	0	0	0	0	0	0	0	0	0	0	0	0	1	5	21	0	0	0	0	0	0	0
w	2,0	15	80	0	0	1	0	0	0	0	1	0	1	0	0	0	0	0	0	0	0	0	0	0	0	0	0	1	1	1	0	0	39	0	0	0	0	0	0	0
m	2,0	15	68	0	0	0	1	0	0	0	0	0	0	0	0	0	0	0	0	0	0	0	0	0	0	0	0	0	0	1	0	23	29	0	0	0	0	0	0	0
w	1,9	15	29	0	0	0	0	0	0	0	0	0	0	0	0	0	0	0	0	0	0	0	0	0	0	0	0	0	0	0	0	0	6	0	0	0	0	0	0	0
m	1,4	15	71	0	0	0	0	0	0	0	0	0	0	0	1	0	0	0	0	0	0	0	0	0	0	0	0	0	0	0	1	0	45	0	0	1	0	1	0	0
m	1,1	15	72	0	0	1	1	0	0	0	0	0	0	0	0	0	0	0	0	0	0	0	0	0	0	0	0	0	0	0	0	5	29	0	0	0	1	0	0	1
w	1,2	15	48	0	0	0	0	1	0	0	0	0	0	0	0	0	0	0	1	0	0	0	0	0	0	0	0	0	0	0	0	15	36	0	0	1	0	1	0	1
w	1,0	15	85	0	0	0	1	0	0	0	0	0	0	0	0	0	0	0	0	0	1	1	0	0	0	0	0	0	0	0	0	10	45	0	0	0	0	0	0	0
w	1,0	15	78	0	0	1	0	1	0	0	1	1	1	0	0	0	0	0	0	0	0	0	0	0	0	0	0	0	0	0	1				1	0	0	0	1	0
w	10,9	6	56	0	0	0	0	0	0	0	0	0	0	0	0	0	0	0	1	1	1	1	0	1	1	0	0	0	0	1	1	19	41	0	1	1	0	1	0	1
m	1,2	15	74	0	0	0	0	0	0	0	0	0	0	0	0	0	0	0	0	0	0	0	0	0	0	0	0	0	0	0	1	5	28	0	0	0	0	0	0	0
m	1,8	15	49	0	0	0	0	0	0	0	1	0	0	0	0	0	0	0	0	0	0	0	0	0	0	0	0	0	0	0	0	4	44	0	0	0	0	0	0	1
w	1,5	15	53	0	0	0	0	0	0	0	0	0	0	0	0	0	0	0	0	0	0	0	0	0	0	0	0	0	0	0	0	5	27	0	0	1	1	0	0	0
m	1,7	15	71	0	0	0	0	0	0	0	0	0	1	1	0	0	0	0	0	0	0	0	0	0	0	0	0	0	0	0	0	10	38	0	0	0	0	0	0	0
m	1,1	15	80	0	0	0	0	0	0	0	1	0	0	0	0	0	0	0	0	0	0	0	0	0	0	0	0	0	0	0	0	5	28	0	0	0	0	0	0	0
m	7,8	9	60	0	0	0	0	1	0	0	0	0	0	0	0	0	0	0	0	1	1	0	1	0	1	0	0	0	0	0	1	14	39	0	1	1	0	1	0	0
w	2,8	15	85	0	0	0	0	1	0	0	0	0	0	0	0	0	0	0	0	1	1	1	0	0	0	0	0	0	0	0	0	10	50	1	1	0	1	0	0	0
w	1,7	15	79	0	0	1	0	0	0	0	0	0	0	0	0	0	0	0	1	0	0	0	0	0	0	0	0	0	0	0	1	10	57	1	0	0	0	0	0	0
m	3,1	12	69	0	0	1	0	0	0	1	0	0	0	1	0	0	0	0	0	0	0	0	0	0	0	1	0	0	0	1	0	5	37	0	0	0	1	0	0	1
w	10,1	6	62	0	0	0	0	0	0	0	0	0	1	0	0	0	0	0	0	1	1	1	1	0	0	0	0	0	0	1	1	10	34	0	1	1	0	0	1	0
m	2,9	15	82	0	0	1	0	0	0	1	0	0	0	0	0	0	0	0	0	0	0	0	0	0	0	0	0	0	0	1	0	5	44	0	0	0	0	0	0	1
w	1,8	15	81	0	0	0	0	0	0	0	1	0	0	0	0	0	0	0	0	0	0	0	0	0	0	0	0	0	0	0	1	0	50	1	0	0	0	0	0	0
w	3,4	12	60	0	0	0	0	0	0	0	0	0	0	0	0	0	0	0	0	0	0	0	0	0	0	0	0	0	0	0	0	10	41	0	1	1	0	0	1	0
w	5,5	12	70	0	0	0	0	0	0	0	0	0	0	1	0	0	0	0	0	0	0	0	0	0	0	0	0	0	0	0	0	0	67	1	0	0	0	0	0	1
m	1,1	15	85	0	0	0	1	0	0	0	0	0	0	0	0	0	0	0	0	0	0	0	0	0	0	0	0	0	0	1	0				1	0	0	0	1	0
w	1,4	15	36	0	0	1	1	0	0	0	0	0	0	0	0	0	0	0	0	1	1	1	0	0	0	0	1	0	0	0	1	19	37	0	0	0	0	0	0	0
m	6,1	9	64	0	0	0	0	0	0	1	0	0	0	0	0	0	0	0	0	1	1	1	1	0	0	0	0	0	0	1	1	14	51	1	0	1	1	0	0	0

w	1,2	15	75	0	0	0	0	0	0	0	0	0	0	0	0	0	0	0	0	0	0	0	0	0	0	0	0	0	0	0	1	5	42	0	0	0	0	0	0	0
m	7,1	9	71	0	0	0	1	0	0	0	0	0	0	1	0	0	0	0	1	0	1	1	0	0	0	0	0	0	0	1	0	15	50	1	0	1	1	0	0	0
m	8,8	9	55	0	0	0	0	1	0	0	0	0	0	0	0	0	0	0	1	1	1	1	0	0	0	0	0	0	0	1	0	15	43	0	0	1	1	0	0	0
m	5,7	12	59	0	0	0	1	0	0	0	0	0	0	0	0	0	0	0	0	0	0	0	0	0	0	0	0	0	0	0	0	5	14	0	1	1	0	0	1	1
w	1,1	15	83	0	0	0	1	0	1	0	0	0	0	0	0	0	0	0	0	0	0	0	0	0	0	0	0	0	0	0	0	10	31	0	0	0	0	0	0	0
m	6,0	9	30	0	0	0	0	0	0	0	0	0	0	0	0	0	0	0	0	1	1	1	1	0	0	0	0	0	0	0	0	19	22	0	0	0	0	0	0	0
m	1,8	15	90	0	0	0	1	0	0	0	1	0	0	0	0	0	0	0	0	1	1	1	0	0	0	0	0	0	0	1	0	10	41	0	0	1	1	0	0	0
w	3,1	12	32	0	0	0	0	0	0	0	0	0	0	0	0	0	0	0	0	0	0	0	0	0	0	0	0	0	0	0	0	0	15	0	0	0	0	0	0	0
m	1,3	15	60	0	0	0	0	0	0	0	0	1	1	0	0	0	0	0	0	0	0	0	0	0	0	0	0	0	0	0	1	5	29	0	0	0	0	0	0	0
m	1,1	15	55	0	0	0	0	0	0	0	0	0	0	0	0	0	0	0	0	0	0	0	0	0	0	0	0	0	0	0	0	0	15	0	0	0	0	0	0	0
w	5,1	12	23	0	0	0	0	0	0	0	0	0	0	0	0	0	0	0	0	0	0	0	0	0	0	0	0	0	0	0	0	0	15	0	0	0	0	0	0	0
m	9,2	6	81	0	0	1	1	0	1	0	0	0	0	0	0	0	0	0	1	1	1	1	1	0	0	0	0	0	0	1	0	10	47	0	1	1	0	0	1	1
m	4,1	12	41	0	0	0	0	0	0	0	0	0	0	0	0	0	0	0	1	0	1	1	0	0	0	0	0	0	0	0	1	19	19	0	0	0	0	0	0	0
m	3,2	12	73	0	0	0	0	0	1	0	0	0	0	1	0	0	0	0	1	1	0	1	0	0	0	0	0	0	0	0	0	10	57	1	0	1	0	0	1	0
w	1,1	15	64	0	0	0	0	0	0	0	0	0	0	0	1	0	0	0	0	0	0	0	0	0	0	0	0	0	0	0	1	5	20	0	0	0	0	0	0	0
m	1,1	15	78	0	0	1	0	0	0	0	0	0	0	0	0	0	0	0	0	0	0	0	0	0	0	0	0	0	0	0	0	5	19	0	1	1	0	1	0	0
w	2,7	15	82	0	0	0	1	0	0	0	0	0	0	0	0	0	0	0	0	0	1	0	0	0	1	0	0	0	0	0	0	22	34	0	0	0	0	0	0	0
W	13,7	3	51	0	0	0	0	0	1	0	0	0	0	0	1	0	0	0	1	1	1	1	0	1	0	0	0	0	0	1	0	19	33	0	0	1	0	1	0	0
m	1,9	15	65	0	0	0	1	0	0	1	0	1	0	0	0	0	0	0	0	0	1	1	0	0	0	0	0	0	0	1	0	10	62	1	0	1	1	0	0	0
m	2,9	15	66	0	0	0	0	0	0	0	0	0	0	0	0	0	0	0	0	0	0	0	0	0	0	0	0	0	0	0	1	5	25	0	0	1	1	0	0	1
m	1,9	15	38	0	0	0	0	0	0	0	0	0	0	0	1	0	0	0	0	1	1	1	0	0	0	0	0	0	0	0	0	10	23	0	0	1	0	0	1	0
m	1,9	15	52	0	0	0	1	0	0	0	1	0	0	0	0	0	0	0	0	0	0	0	0	0	0	0	0	0	0	0	0	0	30	0	0	0	0	0	0	1
m	1,1	15	69	0	0	0	1	0	0	0	0	0	0	0	0	0	0	0	0	0	0	0	0	0	0	0	0	0	0	0	1	0	22	0	0	0	0	0	0	0
w	1,5	15	48	0	0	0	0	0	0	0	0	0	0	0	0	0	0	0	0	0	0	0	0	0	0	0	0	0	0	0	0	10	40	0	0	0	0	0	0	0
w	1,7	15	77	0	0	0	0	0	0	0	0	0	1	0	0	0	0	0	0	0	1	1	0	0	0	0	0	0	0	0	0	5	42	0	0	0	0	0	0	0
m	1,2	15	71	0	0	0	1	0	0	0	0	0	0	0	0	0	0	0	0	0	0	0	0	0	0	0	0	0	0	0	1	5	41	0	0	0	0	0	0	1
m	1,0	15	37	0	0	0	0	0	0	0	0	0	0	0	0	0	0	0	0	0	1	1	0	0	0	0	0	0	0	1	0	10	28	0	0	0	0	0	0	0
m	2,3	15	79	0	0	0	0	0	0	0	0	0	0	0	0	0	0	0	1	0	0	0	0	0	0	0	0	0	0	0	1	10	32	0	0	0	0	0	0	0
m	1,5	15	46	0	0	0	0	0	0	1	0	0	0	1	0	0	0	0	1	0	0	1	0	0	0	0	0	0	0	1	0	9	47	0	0	0	0	0	0	0
w	1,1	15	80	0	0	0	1	1	0	0	0	0	0	0	0	0	0	0	0	0	0	0	0	0	0	0	0	0	0	0	0	5	31	0	0	0	0	0	0	0
w	1,6	15	52	0	0	0	0	0	0	0	0	0	1	0	1	0	1	0	0	1	0	0	0	0	0	0	0	0	0	0	0	5	29	0	0	1	1	0	0	0
m	1,1	15	59	0	0	0	0	0	0	0	0	0	0	1	0	0	0	0	0	0	0	0	0	0	0	0	0	0	0	0	0	0	20	0	0	0	0	0	0	0
w	1,0	15	83	0	0	1	0	0	0	0	0	0	1	0	0	0	0	0	0	0	0	0	0	0	0	0	0	0	0	0	0				0	0	1	0	0	0
m	1,9	15	79	0	0	1	1	0	0	0	0	0	0	0	0	0	0	0	0	1	1	0	1	0	0	0	0	0	0	0	1	15	45	0	0	0	0	0	0	0
m	6,6	9	71	0	0	0	0	0	1	0	0	0	0	0	0	0	0	0	1	1	0	0	0	0	0	0	0	0	0	0	0	15	51	1	0	0	1	0	0	0
m	1,1	15	27	0	0	0	0	0	0	0	0	0	0	0	0	0	1	1	0	1	0	0	0	0	0	0	0	0	0	0	0	5	16	0	0	1	1	0	0	0
m	5,4	12	66	0	0	0	1	0	0	0	1	0	1	1	0	0	0	0	0	0	1	1	1	0	0	0	0	0	0	0	0	9	62	1	1	1	0	0	1	1
m	2,1	15	27	0	0	0	0	0	0	0	0	0	1	0	0	0	0	0	1	0	0	0	0	0	0	0	0	0	0	0	0	10	36	0	0	0	0	0	0	0
w	3,7	12	69	0	0	0	0	1	0	0	0	0	0	0	0	0	0	0	0	0	0	0	0	0	0	0	0	0	0	0	0	5	20	0	1	1	0	1	0	0
w	8,1	9	68	0	0	0	1	1	0	0	0	0	0	0	0	0	0	0	0	1	1	1	0	1	1	0	0	0	0	1	1	23	63	1	0	1	1	0	0	0
m	1,1	15	87	0	0	0	0	0	0	0	1	0	0	1	0	0	0	0	0	0	0	0	0	0	0	0	0	0	0	0	0	5	59	1	0	0	0	0	0	0
w	1,1	15	93	0	0	1	0	0	0	0	0	1	0	0	0	0	0	0	0	0	0	0	0	0	0	0	0	0	0	0	0	5	39	0	0	0	0	0	0	0
w	2,1	15	84	0	0	1	0	0	0	0	0	1	1	0	0	0	0	0	0	0	0	0	0	0	0	0	0	0	0	0	1	10	31	0	1	1	0	0	1	0
m	4,8	12	80	0	0	0	0	0	1	0	0	0	0	1	0	0	0	0	1	1	1	1	0	0	0	0	0	0	0	1	0	23	65	1	0	1	1	0	0	1
m	1,5	15	59	0	0	0	0	0	0	0	0	0	0	0	0	0	0	0	0	0	0	0	0	0	0	0	0	0	0	0	1	5	13	0	0	0	0	0	0	0
w	1,6	15	31	0	0	0	0	0	0	0	0	0	0	0	1	0	0	0	0	0	0	0	0	0	0	0	0	0	0	0	0	0	11	0	0	0	0	1	0	0
w	1,2	15	19	0	0	0	0	0	0	0	0	0	0	1	0	0	0	0	0	0	0	0	0	0	0	0	0	0	0	0	0	0	15	0	0	0	0	0	0	1
m	1,3	15	79	0	0	0	0	0	0	0	0	0	0	0	0	0	0	0	0	0	0	0	0	0	0	0	0	0	0	0	0	5	24	0	0	0	0	0	0	0
m	2,4	15	33	0	0	0	0	0	0	0	0	0	0	0	0	0	0	0	0	0	0	0	0	0	0	0	0	1	0	1	0	3	13	0	0	0	0	0	0	1
w	1,8	15	72	0	0	0	0	0	0	0	0	0	0	0	0	0	0	0	0	0	0	0	0	0	0	0	0	0	0	0	1	5	28	0	0	0	0	0	0	0
m	16,2	0	43	0	0	0	0	0	0	0	0	0	0	0	0	0	1	0	0	1	1	1	0	1	0	1	0	0	0	0	0	14	25	0	1	1	0	0	1	1
m	14,0	3	68	0	0	1	1	0	1	0	0	0	0	0	0	0	0	0	1	1	1	1	1	0	1	0	0	0	0	1	0	10	28	0	1	1	0	1	0	1
m	15,2	0	64	0	0	0	0	0	0	0	0	0	0	1	0	0	0	0	0	1	1	1	1	0	0	0	0	0	0	1	0	15	51	1	0	1	0	0	1	0
w	1,1	15	89	0	0	1	0	0	0	0	0	0	0	0	0	0	0	0	0	0	0	0	0	0	0	0	0	0	0	0	0	5	28	0	0	1	0	0	1	0
w	2,0	15	80	0	0	0	0	0	0	0	0	0	0	0	0	0	0	0	0	0	0	0	0	0	0	0	0	0	0	0	1	0	26	0	0	0	0	0	0	0
w	4,1	12	41	0	0	0	0	0	0	0	0	0	0	1	0	0	0	0	0	0	0	0	0	0	0	0	0	0	0	0	0	18	36	0	0	1	1	0	0	1
m	1,6	15	57	0	0	0	0	0	0	0	0	0	0	0	0	0	0	0	0	0	0	0	0	0	0	0	0	0	0	0	0	0	18	0	0	0	0	0	0	0
w	1,3	15	44	0	0	0	0	0	0	0	0	0	0	1	0	0	0	0	0	0	0	0	0	0	0	0	0	0	0	1	0	0	51	1	0	0	0	0	0	0
w	1,7	15	72	0	0	1	0	0	0	1	0	0	0	0	0	0	0	0	0	0	1	1	0	0	0	0	0	0	0	1	0	9	52	1	0	0	0	0	0	1
m	4,7	12	69	0	0	0	1	0	0	0	0	1	1	0	0	0	0	0	0	0	0	0	0	0	0	0	0	1	0	0	0	12	44	0	1	1	0	0	1	1
m	1,2	15	54	0	0	0	0	0	0	0	0	0	1	0	0	0	0	0	0	0	0	0	0	0	0	0	0	0	0	0	1	5	15	0	0	0	0	0	0	1
m	5,7	12	76	0	0	1	0	0	0	0	1	0	0	1	0	0	0	0	0	0	1	1	0	1	0	0	0	0	0	0	0	15	57	1	1	1	0	0	1	1
w	3,1	12	33	0	0	0	0	0	1	0	0	0	1	0	1	0	1	0	0	0	0	0	0	0	0	0	0	0	0	0	0	9	39	0	0	1	0	0	1	0
m	6,8	9	63	0	0	0	0	1	0	0	0	0	0	0	0	0	0	0	0	1	1	0	1	0	0	0	0	0	0	0	1	15	57	1	1	1	0	0	1	1
w	3,6	12	79	0	0	0	0	0	0	0	0	0	0	0	0	0	0	0	0	0	0	0	0	0	0	0	0	0	0	0	0	9	34	0	0	1	0	0	1	0
m	1,2	15	63	0	0	0	0	0	0	0	0	0	0	0	0	0	0	0	0	0	0	0	0	0	0	0	0	0	0	0	1	0	41	0	0	0	1	0	0	0
m	1,6	15	19	0	0	0	0	0	0	0	0	0	0	0	0	0	0	0	0	0	0	0	0	0	0	0	0	0	0	0	0	5	13	0	0	0	0	0	0	0
m	8,2	9	52	0	0	1	0	0	0	0	0	0	0	0	1	0	0	0	1	1	1	0	1	0	0	0	0	0	0	0	1	10	38	0	0	1	0	0	1	0
w	1,8	15	91	0	0	0	0	0	0	0	0	0	0	0	0	0	0	0	0	0	0	0	0	0	0	0	0	0	0	0	1	5	29	0	1	1	0	0	1	0

w	4,1	12	47	0	0	0	0	0	1	0	0	0	0	0	0	0	1	0	1	0	0	0	0	0	0	0	0	0	0	0	0	10	32	0	1	1	0	0	1	0
m	1,1	15	80	0	0	0	0	0	0	0	1	0	0	0	0	0	0	0	0	0	0	0	0	0	0	0	0	0	0	0	0	5	39	0	0	0	0	0	0	0
w	1,5	15	64	0	0	0	0	0	0	0	0	0	0	1	0	0	0	0	1	0	1	1	1	0	0	0	0	0	0	1	0	10	63	1	0	0	1	0	0	0
m	1,3	15	60	0	0	0	0	1	1	0	0	0	0	0	0	0	0	0	0	0	0	0	0	0	0	0	0	0	0	0	0	5	18	0	0	0	0	0	0	0
m	1,1	15	71	0	0	0	0	0	0	1	0	0	0	0	0	0	1	0	1	0	1	1	0	0	0	0	0	0	0	0	0	10	52	1	0	1	0	0	1	0
m	20,4	0	69	0	0	0	0	0	0	0	0	0	1	0	0	0	0	0	1	1	1	1	0	0	0	0	0	0	0	1	0	24	37	0	0	1	0	0	1	0
w	6,7	9	70	0	0	1	0	0	1	0	1	0	0	0	1	0	0	0	0	0	0	0	0	0	0	0	0	1	1	1	0	8	45	0	0	1	1	0	0	0
m	2,9	15	28	0	0	0	0	0	0	0	0	0	1	0	0	0	0	0	0	0	0	0	0	0	0	0	0	0	0	0	0	5	15	0	0	0	0	0	0	0
w	1,8	15	29	0	0	0	0	0	0	0	0	0	0	0	0	0	0	0	0	0	0	0	0	0	0	0	0	0	0	0	1	5	19	0	0	0	0	0	0	0
w	1,3	15	78	0	0	0	0	0	0	0	0	0	1	0	0	0	0	0	0	0	0	0	0	0	0	0	0	0	0	0	1	5	52	1	0	0	0	0	0	0
w	3,1	12	75	0	0	0	0	0	0	0	0	0	0	1	0	0	0	0	0	0	0	0	0	0	0	0	0	0	0	0	0				1	1	1	0	0	1
w	1,0	15	90	0	0	1	0	0	0	0	0	0	1	0	0	0	0	0	0	0	0	0	0	0	0	0	0	0	0	0	0	5	32	0	0	0	0	0	0	0
w	2,1	15	82	0	0	1	0	0	0	0	0	0	1	0	0	0	0	0	0	0	0	0	0	0	0	0	0	0	0	0	1	0	26	0	0	1	1	0	0	0
m	4,8	12	79	0	0	0	0	0	1	0	0	0	0	0	0	1	0	0	0	1	1	1	0	0	0	0	0	0	0	0	0	15	51	1	1	1	0	0	1	0
m	10,2	6	60	0	0	0	0	0	0	1	0	0	0	1	0	0	0	0	1	0	0	0	0	0	0	0	0	1	0	1	0	14	44	0	0	0	0	0	0	1
w	8,9	9	70	0	0	0	0	0	0	0	0	0	1	0	0	0	0	0	1	1	1	1	0	1	0	0	0	0	0	1	0	14	38	0	1	1	0	0	1	1
w	4,7	12	73	0	0	0	0	0	1	0	0	0	0	0	0	0	0	0	1	0	0	0	0	0	0	0	0	0	0	0	0	10	38	0	0	0	0	0	0	1
m	1,1	15	83	0	0	1	1	0	0	0	0	0	0	0	0	0	0	0	0	0	0	0	0	0	0	0	0	0	0	0	1	5	30	0	0	0	0	0	0	0
m	1,8	15	73	0	0	1	0	0	0	0	0	0	0	1	0	0	0	0	0	0	0	0	0	0	0	0	0	0	0	0	0	0	64	1	0	0	0	0	0	1
w	1,2	15	61	0	0	0	0	1	0	0	0	0	0	0	1	0	0	0	0	0	0	0	0	0	0	0	0	0	0	0	1	0	36	0	0	0	0	0	0	0
w	3,6	12	83	0	0	0	0	0	0	0	0	0	0	0	0	1	0	0	0	0	1	1	0	0	0	0	0	0	0	0	0	5	35	0	0	1	1	0	0	0
m	1,5	15	33	0	0	0	0	0	0	0	1	0	0	0	0	0	0	0	0	0	0	0	0	0	0	0	0	1	1	0	0	0	19	0	0	0	1	0	0	0
w	2,4	15	85	0	0	0	1	0	0	0	0	0	0	0	0	0	0	0	0	0	1	1	0	0	0	0	0	0	0	1	1	15	36	0	0	0	0	0	0	0
m	2,0	15	71	0	0	0	1	0	0	0	0	1	0	0	0	0	0	0	1	0	0	0	0	0	0	0	0	0	0	0	1	5	27	0	1	1	0	1	0	0
w	1,2	15	65	0	0	0	0	0	1	0	0	0	0	0	0	0	0	0	0	0	0	0	0	0	0	0	0	0	0	0	1	5	32	0	0	0	0	0	0	0
w	1,2	15	42	0	0	0	0	0	0	0	0	0	0	0	0	0	0	0	0	0	0	0	0	0	0	0	0	0	0	0	0	0	28	0	0	0	0	0	0	0
m	7,9	9	66	0	0	0	0	0	0	0	0	0	0	0	0	0	0	0	0	1	1	0	1	0	1	0	1	0	0	1	1	23	57	1	1	1	1	0	0	1
m	1,0	15	80	0	0	0	1	0	0	0	0	0	0	0	0	0	0	0	0	0	0	0	0	0	0	0	0	0	0	0	0	5	31	0	0	0	0	0	0	0
m	8,6	9	78	0	0	0	1	0	0	0	1	0	1	0	0	0	0	0	1	1	1	1	1	0	1	1	1	0	0	0	0	19	51	1	0	1	0	0	1	1
m	12,8	3	66	0	0	1	0	0	0	1	1	0	0	0	0	0	0	0	1	1	1	1	0	1	0	1	0	1	0	1	0	21	64	1	1	1	0	0	1	1
m	1,1	15	43	0	0	0	0	0	1	0	0	0	0	0	0	0	1	1	0	0	0	0	0	0	0	0	0	0	0	0	0	5	22	0	0	1	0	0	1	0
m	2,2	15	84	0	0	1	0	1	0	0	1	0	0	0	0	0	0	0	0	0	0	0	0	0	0	0	0	0	0	0	0	0	35	0	0	0	0	0	0	0
w	1,8	15	85	0	0	1	1	0	0	0	1	0	0	0	0	0	0	0	0	0	0	0	0	0	0	0	0	0	0	0	0	5	34	0	0	0	1	0	0	0
m	1,3	15	80	0	0	0	1	0	0	0	0	0	0	0	0	0	0	0	0	0	0	0	0	0	0	0	0	0	0	0	0	0	37	0	0	0	0	0	0	1
m	1,9	15	29	0	0	1	0	0	0	0	0	0	0	0	0	0	0	0	0	1	0	0	0	0	0	0	0	0	0	0	0	10	24	0	0	0	0	0	0	0
w	7,5	9	79	0	0	1	0	0	1	0	0	1	0	0	0	0	0	0	1	0	0	0	0	0	0	0	0	0	0	0	0	10	57	1	0	0	0	0	0	0
m	3,3	12	48	0	0	0	0	0	0	0	0	0	0	0	0	0	0	0	0	1	0	0	0	0	0	0	0	0	0	0	0	19	32	0	0	1	0	0	1	0
m	17,0	0	48	0	0	0	0	0	0	1	0	0	1	0	0	0	1	0	1	1	1	1	0	1	0	0	0	0	0	0	0	14	39	0	0	1	0	0	1	1
w	2,2	15	38	0	0	0	0	0	0	0	0	0	1	0	0	0	0	0	0	0	0	0	0	0	0	0	0	0	0	0	0	0	25	0	0	0	0	0	0	1
w	2,6	15	65	0	0	0	0	0	1	0	0	0	0	0	1	0	0	0	0	0	0	0	0	0	0	0	0	0	0	0	0	0	24	0	1	1	0	0	1	0
m	18,7	0	64	0	0	0	0	0	0	0	0	0	0	0	0	0	0	0	1	1	1	1	1	0	0	0	1	0	0	1	1	28	52	1	1	1	0	0	1	1
m	1,7	15	70	0	0	1	0	1	1	0	0	1	0	0	0	0	0	0	0	1	1	1	0	0	0	0	0	0	0	1	1	14	48	0	1	1	0	1	0	1
w	1,2	15	62	0	0	0	0	0	0	0	0	0	0	0	0	0	0	0	0	0	0	0	0	0	0	0	0	0	0	0	1	9	21	0	0	0	0	0	0	0
m	1,2	15	84	0	0	0	1	0	0	0	1	0	0	1	0	0	0	0	0	0	0	0	0	0	0	0	0	0	0	0	0	0	44	0	0	1	0	0	1	1
m	1,1	15	92	0	0	1	1	0	0	0	0	0	0	0	0	0	0	0	0	0	0	0	0	0	0	0	0	0	0	1	0	5	35	0	0	1	1	0	0	0
m	7,3	9	73	0	0	0	1	0	0	0	1	0	0	1	0	0	0	0	1	0	0	0	0	0	0	0	0	0	0	0	0	14	53	1	0	0	0	0	0	1
m	2,4	15	50	0	0	0	1	0	1	0	0	0	0	0	1	0	0	0	0	0	0	0	0	0	0	0	0	0	0	0	0	13	33	0	0	1	0	0	1	1
w	1,2	15	81	0	0	0	1	0	1	0	1	0	0	0	0	0	0	0	0	0	0	0	0	0	0	0	0	0	0	0	0	10	54	1	0	0	0	0	0	0
m	2,8	15	82	0	0	0	1	0	0	0	1	0	0	0	1	1	0	0	1	0	0	0	0	0	0	0	0	0	0	0	1	14	45	0	1	1	0	0	1	0
w	1,9	15	64	0	0	0	0	0	0	0	0	0	0	0	0	0	0	0	1	0	0	0	0	0	0	0	0	1	1	1	0	13	53	1	0	0	0	0	0	0
m	8,0	9	64	0	0	0	1	0	0	0	0	1	0	0	0	0	0	1	1	1	1	1	1	0	0	0	0	0	0	0	1	10	38	0	1	1	0	0	1	0
w	1,1	15	73	0	0	1	0	0	1	0	1	0	0	0	1	0	0	1	0	0	0	0	0	0	0	0	0	0	0	1	0	0	31	0	0	1	1	0	0	0
m	1,7	15	57	0	0	0	1	0	0	1	0	0	1	0	0	0	0	0	0	0	0	0	0	0	0	0	0	0	0	1	0	5	33	0	0	1	1	0	0	0
w	8,2	9	82	0	0	0	0	0	0	0	1	0	0	0	0	0	0	0	1	0	1	1	1	0	0	0	0	0	0	0	0	14	61	1	1	1	1	0	0	1
w	3,9	12	53	0	0	0	0	1	0	0	0	0	0	0	0	0	1	1	1	0	1	1	0	0	0	0	1	0	0	0	0	10	35	0	1	1	0	0	1	1
w	2,6	15	77	0	0	0	0	0	0	0	0	0	0	0	0	0	0	0	0	0	0	0	0	0	0	0	1	0	0	0	0	0	33	0	0	0	0	0	0	0
m	3,4	12	76	0	0	0	0	0	0	0	1	0	1	0	0	0	0	0	0	0	0	0	0	0	0	0	0	0	0	0	1	5	40	0	0	1	1	0	0	1
w	5,2	12	32	0	0	0	0	0	0	0	0	0	0	0	0	0	0	0	0	0	0	0	0	0	0	0	0	1	0	1	0	7	8	0	0	0	0	0	0	1
w	1,1	15	67	0	0	0	1	0	1	0	0	0	0	0	0	0	0	0	0	0	0	0	0	0	0	0	0	0	0	0	1	5	26	0	0	0	0	0	0	0
m	6,2	9	65	0	0	0	0	0	0	0	0	0	1	0	0	0	0	0	0	0	0	0	0	0	0	0	0	0	0	0	0	5	37	0	0	1	0	1	0	0
w	5,0	12	78	0	0	0	0	1	0	0	0	0	0	0	0	0	0	0	1	0	0	0	0	0	0	0	0	0	0	0	1	15	39	0	0	0	0	0	0	0
m	4,6	12	79	0	0	0	0	0	0	0	0	0	0	1	0	0	0	0	0	0	1	1	0	0	0	0	0	1	0	1	0	5	46	0	0	1	1	0	0	1
m	1,1	15	89	0	0	0	0	0	0	0	1	0	0	0	0	0	0	0	0	0	0	0	0	0	0	0	0	0	0	0	0	0	47	0	0	0	0	0	0	0
m	1,5	15	67	0	0	0	0	0	0	0	0	0	0	0	0	0	0	0	1	1	0	0	0	0	0	0	0	0	0	0	1	15	34	0	0	0	0	0	0	1
w	1,2	15	51	0	0	0	0	0	0	0	0	0	0	1	0	0	0	0	0	0	0	0	0	0	0	0	0	0	0	0	0	10	31	0	0	1	1	0	0	0
m	1,9	15	24	0	0	0	0	0	0	0	1	0	0	0	0	0	0	0	0	0	0	0	0	0	0	0	0	0	0	0	0	0	13	0	0	0	0	0	0	0
w	5,1	12	86	0	0	0	1	0	0	0	0	0	0	0	0	0	0	0	1	0	1	1	0	0	0	0	0	0	0	0	0	9	45	0	0	1	1	0	0	0
m	1,1	15	54	0	0	0	0	0	0	0	0	0	0	0	0	0	0	0	0	0	0	0	0	0	0	0	0	0	0	0	1	5	25	0	0	0	0	0	0	0
m	1,2	15	64	0	0	1	0	1	1	0	0	0	0	0	0	0	0	0	1	0	0	0	0	0	0	0	0	0	0	0	0	14	38	0	0	0	0	0	0	1

m	14,7	3	76	0	0	0	0	0	0	0	0	0	0	0	0	0	0	0	1	1	1	1	1	0	0	0	0	0	0	0	0	28	49	0	1	1	0	0	1	1
m	11,8	6	45	0	0	0	0	1	1	0	0	0	0	0	1	0	0	0	0	1	1	1	0	0	0	0	0	0	0	0	0	10	51	1	0	0	0	0	0	1
m	1,5	15	86	0	0	0	1	0	0	0	1	0	1	0	0	0	0	0	0	0	0	0	0	0	0	0	0	0	0	0	0	0	42	0	1	1	0	1	0	0
w	3,8	12	63	0	0	0	0	0	0	0	0	0	1	1	0	0	0	0	0	1	1	1	0	0	0	0	0	0	0	1	0	19	51	1	0	0	0	0	0	0
m	1,7	15	61	0	0	1	0	1	0	0	0	0	0	0	0	0	0	0	0	0	0	0	0	0	0	0	0	0	0	0	0	0	22	0	0	0	0	0	0	1
w	4,9	12	73	0	0	0	0	0	0	0	0	0	0	0	1	0	0	0	0	0	0	0	0	0	0	0	0	0	0	0	0	0	44	0	1	1	0	0	1	1
w	6,8	9	70	0	0	1	0	1	1	0	0	1	1	0	1	0	0	0	1	0	1	1	0	0	0	0	0	0	0	0	0	10	41	0	0	1	1	0	0	1
w	1,1	15	79	0	0	0	1	0	0	0	0	0	0	0	0	0	0	0	0	0	0	0	0	0	0	0	0	0	0	0	0	5	37	0	0	0	1	0	0	0
w	7,9	9	80	0	0	1	1	0	0	0	0	0	1	0	0	0	0	0	0	1	1	1	0	0	1	0	0	0	0	1	1	10	43	0	0	0	0	0	0	0
m	1,6	15	67	0	0	0	0	0	1	0	0	0	0	0	0	0	0	0	1	0	0	0	0	0	0	0	0	0	0	0	0	5	56	1	0	1	1	0	0	1
w	2,6	15	79	0	0	0	1	0	0	0	0	0	0	0	1	1	0	0	1	0	0	0	0	0	0	0	0	0	0	0	1	5	45	0	1	1	0	0	1	1
m	1,2	15	73	0	0	0	0	0	0	0	1	0	0	0	0	0	0	0	0	0	0	0	0	0	0	0	0	0	0	0	1	0	32	0	0	0	1	0	0	1
w	1,2	15	86	0	0	1	0	0	0	0	0	0	1	0	0	0	0	0	0	1	0	0	0	0	0	0	0	0	0	0	0	5	30	0	0	0	0	0	0	0
w	5,8	12	63	0	0	0	0	0	1	0	0	0	0	0	1	0	1	0	0	1	0	0	0	0	0	0	0	1	0	1	1	15	51	1	0	1	1	0	0	1
m	8,2	9	89	0	0	1	0	0	0	0	0	0	1	0	0	0	0	0	0	1	1	1	0	0	0	0	0	0	0	0	0	10	44	0	1	1	0	0	1	0
m	2,9	15	93	0	0	0	1	0	1	0	1	0	0	1	0	1	0	0	0	0	1	1	0	0	0	0	0	0	0	0	1	5	55	1	1	1	0	0	1	1
m	1,0	15	72	0	0	1	0	1	0	0	1	0	0	0	0	0	0	0	0	0	0	0	0	0	0	0	0	0	0	0	1				0	0	0	0	0	1
w	1,8	15	86	0	0	0	0	0	1	0	0	0	0	0	1	0	0	0	1	0	0	0	0	0	0	0	0	0	0	0	0	5	44	0	0	1	0	0	1	0
w	1,3	15	86	0	0	0	0	0	0	0	1	0	0	0	0	0	0	0	0	0	0	0	0	0	0	0	0	0	0	0	0	5	38	0	0	1	1	0	0	0
m	2,2	15	77	0	0	1	1	1	1	0	1	1	0	0	0	0	0	0	0	0	0	0	0	0	0	0	0	0	0	0	1	0	26	0	0	0	0	0	0	0
w	1,2	15	30	0	0	0	0	0	0	0	0	0	0	0	0	0	0	0	0	0	0	0	0	0	0	0	0	0	0	0	0	9	17	0	0	0	0	0	0	0
m	1,6	15	70	0	0	0	0	0	0	1	0	0	0	0	0	0	0	0	0	0	0	0	0	0	0	0	0	0	0	0	0	5	39	0	0	0	0	0	0	1
w	2,5	15	60	0	0	1	0	0	0	0	0	0	0	1	0	0	0	0	0	0	0	0	0	0	0	0	0	0	0	1	0	5	46	0	0	0	1	0	0	1
m	5,4	12	79	0	0	0	1	1	0	0	1	1	0	0	0	0	0	0	0	0	1	1	0	0	1	0	0	0	0	0	0	22	57	1	0	0	0	0	0	1
m	8,1	9	61	0	0	1	0	0	0	0	0	0	0	1	0	0	0	0	0	0	1	1	0	1	0	0	0	0	0	0	0	14	57	1	0	1	1	0	0	1
m	1,3	15	79	0	0	1	0	1	1	0	0	0	0	0	0	0	0	0	0	0	1	1	0	0	0	0	0	0	0	0	0	5	28	0	0	0	0	0	0	0
m	1,7	15	82	0	0	0	0	0	0	0	0	0	0	0	0	0	0	0	0	1	1	1	0	1	0	0	0	0	0	1	0	28	60	1	0	0	0	0	0	1
m	1,7	15	42	0	0	0	0	0	0	0	0	0	0	0	0	0	0	0	0	1	1	1	1	0	0	0	0	0	0	1	0	23	56	1	0	0	0	0	0	1
w	2,5	15	45	0	0	0	0	0	0	0	0	0	0	0	1	0	0	0	0	1	0	0	0	0	0	0	0	0	0	0	0	10	38	0	0	1	1	0	0	1
m	14,9	3	79	0	0	1	0	1	1	0	1	0	0	0	0	0	0	0	1	0	1	1	0	1	0	0	0	1	0	1	0	18	57	1	1	1	0	0	1	1
m	1,1	15	70	0	0	0	1	1	0	0	0	0	0	1	0	0	0	0	0	0	0	0	0	0	0	0	0	0	0	0	0	10	38	0	0	0	0	0	0	0
m	2,8	15	86	0	0	0	1	0	0	0	0	0	0	0	0	0	0	0	0	0	0	0	0	0	0	0	0	0	0	0	1	0	43	0	0	0	0	0	0	1
w	1,3	15	31	0	0	0	0	0	0	0	0	0	0	0	0	0	0	0	0	0	0	0	0	0	0	0	0	0	0	0	0	5	19	0	0	0	1	0	0	0
m	1,7	15	74	0	0	0	1	0	0	0	0	1	0	0	0	0	1	0	0	0	0	0	0	0	0	0	0	0	0	0	1	5	48	0	0	1	1	0	0	0
m	1,5	15	75	0	0	0	0	0	1	0	0	0	0	0	0	0	0	0	0	0	0	0	0	0	0	0	0	0	0	0	1	5	24	0	0	0	0	0	0	0
m	1,9	15	68	0	0	0	1	0	0	0	0	0	0	0	0	0	0	0	0	0	0	0	0	0	0	0	0	0	0	0	0	0	30	0	0	1	0	0	1	1
w	3,4	12	56	0	0	0	0	0	0	0	0	0	0	0	0	0	0	0	1	0	1	1	0	0	0	0	0	0	0	0	0	15	39	0	0	0	1	0	0	1
m	57,7	0	73	0	0	1	0	0	0	0	1	0	0	0	0	0	0	0	0	0	1	1	1	0	0	0	0	1	1	1	0	19	54	1	1	1	1	0	0	0
m	1,2	15	82	0	0	0	1	0	1	0	0	0	0	0	0	0	0	0	0	1	0	0	0	0	0	0	0	0	0	0	0	10	38	0	0	0	0	0	0	0
m	2,7	15	75	0	0	1	1	0	0	0	0	0	0	0	0	0	0	0	0	0	0	0	0	0	0	0	0	0	0	1	0	5	39	0	0	0	0	0	0	0
w	2,2	15	83	0	0	1	0	0	0	0	0	0	0	0	0	0	0	0	0	0	0	0	0	0	1	0	0	0	0	0	0	5	37	0	0	1	0	1	0	1
m	1,1	15	59	0	0	0	0	0	0	0	0	0	0	0	0	0	0	0	0	0	0	0	0	0	0	0	0	0	0	0	1	5	20	0	0	0	0	0	0	0
w	4,5	12	92	0	0	0	0	0	0	0	1	1	1	0	0	0	0	0	0	1	1	1	0	0	0	0	0	0	0	0	0	15	47	0	0	1	1	0	0	1
w	2,8	15	78	0	0	1	0	0	0	0	0	0	0	0	0	1	0	0	0	0	0	0	0	0	0	0	0	0	0	0	0	5	49	0	1	1	0	1	0	1
m	1,3	15	68	0	0	0	1	0	0	0	1	0	0	0	0	0	0	0	0	0	0	0	0	0	0	0	0	0	0	0	1	0	20	0	0	0	0	0	0	0
m	1,4	15	55	0	0	0	0	0	0	0	0	0	1	1	0	0	0	0	0	0	0	0	0	0	0	0	0	0	0	0	0	0	28	0	0	0	0	0	0	1
m	1,1	15	62	0	0	0	0	0	1	0	0	0	0	1	0	0	0	0	0	0	0	0	0	0	0	0	0	0	0	0	0	0	27	0	0	0	0	0	0	1
m	1,2	15	48	0	0	0	0	0	0	0	0	0	0	0	0	0	0	0	0	0	0	0	0	0	0	0	0	0	0	0	1	0	25	0	0	0	0	0	0	0
m	3,3	12	88	0	0	0	0	0	0	0	0	0	0	0	0	1	0	0	0	0	0	0	0	0	0	0	0	0	0	0	1	0	31	0	1	1	0	0	1	1
w	6,6	9	49	0	0	0	0	0	1	0	0	0	0	0	0	0	0	0	0	1	1	1	0	1	0	0	0	0	0	1	1	10	33	0	1	1	1	0	0	0
w	16,0	0	36	0	0	0	0	1	0	0	0	0	0	0	0	0	0	0	1	0	0	0	0	0	0	0	0	0	0	0	0	10	17	0	0	0	0	0	0	0
m	1,0	15	43	0	0	0	0	0	0	0	0	0	0	0	0	0	0	0	0	0	0	0	0	0	0	0	0	0	0	0	1	0	18	0	0	0	0	0	0	0
m	4,8	12	70	0	0	0	1	1	1	0	0	0	0	0	0	0	0	0	1	0	0	0	0	0	0	0	0	0	0	0	0	10	39	0	0	1	0	1	0	0
w	1,7	15	76	0	0	1	1	0	0	0	0	0	0	0	0	0	0	0	0	0	0	0	0	0	0	0	0	0	0	0	0	5	37	0	0	0	0	0	0	1
m	1,2	15	80	0	0	1	1	0	0	0	0	0	0	1	0	0	0	0	0	0	1	1	0	0	0	0	0	0	0	0	0	5	31	0	1	0	0	0	1	0
m	2,0	15	61	0	0	0	0	0	0	1	0	0	0	1	0	0	0	0	0	0	0	0	0	0	0	0	0	0	0	0	1	0	36	0	0	0	0	0	0	0
w	1,1	15	90	0	0	1	0	0	0	0	0	0	0	0	0	0	0	0	0	0	0	0	0	0	0	0	0	0	0	0	1	5	43	0	0	0	0	0	0	0
m	11,6	6	58	0	0	1	0	0	0	0	0	0	0	1	0	0	1	0	1	0	0	0	0	0	0	0	0	0	0	1	0	19	62	1	1	1	0	0	1	1
m	1,1	15	82	0	0	0	1	0	0	0	0	0	0	0	0	0	0	0	0	0	0	0	0	0	0	0	0	0	0	0	1	5	28	0	0	1	1	0	0	0
m	6,1	9	78	0	0	1	1	0	0	0	1	0	0	1	0	0	0	0	0	1	1	1	0	0	1	0	1	0	0	1	1	27	61	1	1	1	0	0	1	0
m	15,5	0	79	0	0	1	1	0	0	0	0	0	0	0	0	0	0	0	0	1	1	0	0	1	0	0	1	0	0	1	1	14	47	0	0	1	1	0	0	1
w	2,4	15	79	0	0	1	0	0	1	0	0	0	0	0	0	0	0	0	0	1	1	1	0	0	0	0	0	0	0	1	0	19	55	1	0	0	0	0	0	0
m	1,8	15	78	0	0	0	1	0	1	0	0	0	1	0	0	0	0	0	0	0	0	0	0	0	0	0	0	0	0	0	0	0	30	0	1	0	1	0	0	0
m	4,3	12	52	0	0	0	0	0	0	0	0	0	0	0	0	0	0	0	0	1	1	0	1	0	0	0	0	0	0	1	1	15	40	0	1	1	0	0	1	0
m	1,7	15	57	0	0	1	0	0	1	0	0	0	0	0	1	0	0	0	1	0	1	1	0	0	0	0	0	0	0	0	0	10	41	0	0	1	1	0	0	0
m	4,8	12	29	0	0	0	0	0	0	0	0	0	0	0	0	0	0	0	0	1	1	1	0	0	0	0	0	0	0	0	1	15	31	0	0	0	0	0	0	1
m	3,1	12	80	0	0	0	0	0	1	0	0	1	0	1	0	0	0	0	1	0	0	0	0	0	0	0	0	0	0	0	0	10	47	0	0	0	0	0	0	0
w	2,3	15	87	0	0	1	0	0	0	1	1	0	1	0	0	0	0	0	0	0	1	1	0	0	0	0	0	0	0	0	0	5	39	0	0	0	0	0	0	1
w	1,5	15	50	0	0	0	0	0	0	0	0	0	0	0	0	0	0	0	0	0	0	0	0	0	0	0	0	1	0	1	0	10	15	0	0	0	0	0	0	1

m	1,2	15	56	0	0	0	0	0	0	0	0	0	0	0	0	0	0	0	0	0	0	0	0	0	0	0	0	0	0	0	0	9	26	0	0	0	0	0	0	1
m	1,8	15	35	0	0	0	0	0	0	0	0	0	0	0	1	0	0	0	0	1	0	0	0	0	0	0	0	0	0	0	0	10	26	0	0	0	0	0	0	0
w	7,7	9	79	0	0	1	0	1	0	0	0	0	0	0	0	0	0	0	0	1	1	0	0	1	1	0	0	0	0	0	1	31	44	0	1	1	0	1	0	1
m	2,0	15	90	0	0	0	1	1	1	0	0	0	0	0	0	0	0	0	0	0	0	0	0	0	0	0	0	0	0	0	1	5	31	0	0	1	1	0	0	0
m	1,2	15	59	0	0	0	0	1	0	0	0	0	0	0	0	0	0	0	0	0	0	0	0	0	0	0	0	0	0	0	1	5	31	0	0	1	1	0	0	0
w	1,0	15	52	0	0	0	0	0	1	0	0	1	0	0	0	0	0	0	0	0	0	0	0	0	0	0	0	0	0	0	1	5	26	0	0	0	0	0	0	0
w	2,2	15	81	0	0	0	0	0	0	0	0	0	0	0	0	0	0	0	1	0	1	1	0	0	0	0	1	0	0	0	1	14	45	0	0	1	1	0	0	1
w	6,3	9	74	0	0	0	1	0	1	0	0	0	0	0	0	0	0	0	0	1	1	1	0	1	0	0	0	0	0	1	1	19	65	1	1	1	0	1	0	1
w	4,9	12	69	0	0	0	0	0	0	0	0	0	0	1	0	0	0	0	1	0	1	1	0	0	0	0	0	0	0	1	0	10	63	1	0	1	0	0	1	1
m	3,7	12	79	0	0	1	0	0	0	0	0	0	1	0	0	0	0	0	0	0	1	1	0	0	0	0	0	0	0	1	0	14	48	0	1	1	0	1	0	0
w	1,9	15	65	0	0	0	0	0	0	0	0	0	0	0	0	0	0	0	0	0	0	0	0	0	0	0	0	0	0	0	1	0	36	0	0	0	0	0	0	0
m	4,5	12	80	0	0	1	0	0	0	0	0	0	0	0	0	0	0	0	0	1	1	1	0	0	0	0	0	0	0	0	1	10	63	1	0	1	0	1	0	1
m	2,6	15	61	0	0	0	0	0	0	0	0	0	1	0	0	0	0	0	0	0	0	0	0	0	0	0	0	0	0	0	0	0	34	0	0	0	0	0	0	1
m	19,1	0	50	0	0	0	0	0	0	1	0	0	0	0	0	0	1	0	0	1	1	1	0	0	0	0	0	0	0	1	0	28	47	0	0	1	1	0	0	1
m	2,2	15	25	0	0	0	0	0	0	0	0	0	1	0	1	0	0	0	0	0	0	0	0	0	0	0	0	0	0	0	0	9	8	0	0	1	0	0	1	0
m	1,5	15	20	0	0	0	0	0	0	0	0	0	0	0	1	0	0	0	0	0	0	0	0	0	0	0	0	0	0	0	0	5	15	0	0	0	1	0	0	0
w	5,8	12	75	0	0	0	0	0	1	0	0	0	0	0	0	0	0	0	0	0	1	1	1	0	0	0	0	1	0	1	0	10	48	0	0	0	0	0	0	1
w	1,0	15	72	0	0	0	0	1	0	0	0	0	0	0	0	0	0	0	0	1	0	0	0	0	0	0	0	0	0	0	0	10	48	0	0	0	0	0	0	1
m	2,7	15	39	0	0	0	0	0	0	1	0	0	0	0	0	0	1	1	1	0	0	0	0	0	0	0	0	0	0	0	0	15	20	0	0	0	0	0	0	0
m	12,9	3	22	0	0	0	0	0	0	0	0	0	0	0	0	0	1	1	0	1	1	1	0	0	0	0	0	1	0	1	0	35	61	1	0	1	0	0	1	1
w	1,1	15	91	0	0	0	1	0	0	0	1	0	0	0	0	0	0	0	0	0	0	0	0	0	0	0	0	0	0	0	1	5	35	0	0	0	0	0	0	0
m	3,1	12	89	0	0	0	0	0	1	0	1	0	0	0	0	0	0	0	1	0	1	1	0	0	0	0	0	0	0	0	1	10	50	1	0	1	1	0	0	1
m	2,3	15	38	0	0	0	0	0	0	0	0	0	1	0	1	0	1	1	0	1	0	0	0	0	0	0	0	0	0	0	0	10	47	0	0	1	1	0	0	1
m	1,2	15	74	0	0	0	1	0	0	0	0	0	0	0	0	0	0	0	0	0	0	0	0	0	0	0	0	0	0	0	1	5	35	0	0	0	0	0	0	0
m	1,2	15	78	0	0	0	0	0	0	0	0	0	0	0	0	0	0	0	0	0	0	0	0	0	0	0	0	0	0	0	1	10	44	0	0	0	0	0	0	0
w	1,9	15	92	0	0	0	0	0	0	0	1	0	0	0	0	1	0	0	0	0	0	0	0	0	0	0	0	0	0	0	0	0	35	0	0	1	1	0	0	1
w	7,5	9	67	0	0	0	0	0	0	0	0	0	0	0	0	0	0	0	0	0	1	1	0	0	0	0	0	0	0	0	0	5	53	1	1	1	1	0	0	1
m	1,6	15	70	0	0	1	0	0	0	0	0	1	1	0	1	0	0	0	1	0	0	0	0	0	0	0	0	0	0	0	0	10	33	0	1	1	1	0	0	1
m	1,2	15	60	0	0	0	0	0	0	1	0	1	0	1	0	0	0	0	0	0	0	0	0	0	0	0	0	0	0	1	0	10	67	1	0	0	0	0	0	1
m	4,6	12	61	0	0	0	1	0	0	1	0	0	0	1	1	0	1	0	1	0	0	0	0	0	0	0	0	0	0	0	0	10	71	1	0	1	0	0	1	1
m	2,5	15	78	0	0	1	1	0	0	0	0	0	0	0	0	0	0	0	0	0	0	0	0	0	0	0	0	0	0	0	1	0	39	0	1	1	0	1	0	1
m	1,1	15	74	0	0	0	0	0	0	0	0	0	0	0	0	0	0	0	0	0	0	0	0	0	0	0	0	0	0	0	1	5	23	0	0	0	0	0	0	0
w	1,0	15	79	0	0	0	1	0	0	0	0	0	0	0	0	0	0	0	0	0	0	0	0	0	0	0	0	0	0	0	1	5	24	0	0	0	0	0	0	0
w	1,5	15	40	0	0	0	0	0	0	0	0	0	0	0	0	0	0	0	0	0	0	0	0	0	0	0	0	0	0	0	0	5	37	0	0	0	0	0	0	1
w	1,2	15	79	0	0	0	0	0	0	0	0	0	0	0	0	0	0	0	0	1	1	1	0	0	0	0	0	0	0	1	0	15	45	0	0	0	0	0	0	0
w	1,1	15	67	0	0	0	1	0	1	0	1	0	0	0	0	0	0	0	0	0	1	1	0	0	0	0	0	0	0	0	0	5	25	0	0	0	0	0	0	0
m	1,3	15	71	0	0	0	0	0	0	0	0	0	1	0	0	0	0	0	0	0	0	0	0	0	0	0	0	0	0	0	1	0	28	0	0	0	0	0	0	0
m	1,0	15	85	0	0	0	1	0	0	0	0	0	1	0	0	0	1	0	0	0	0	0	0	0	0	0	0	0	0	0	0	0	37	0	0	1	1	0	0	0
m	1,1	15	70	0	0	0	1	1	0	0	0	1	0	1	0	0	0	0	0	0	0	0	0	0	0	0	0	0	0	1	0	10	35	0	0	0	0	0	0	0
m	1,0	15	83	0	0	1	0	0	1	0	0	0	0	0	0	0	0	0	0	0	1	1	0	0	0	0	0	0	0	0	0				0	0	0	0	0	0
m	1,8	15	83	0	0	0	0	0	0	0	0	0	0	1	0	0	0	0	0	0	0	0	0	0	0	0	0	0	0	0	0	5	53	1	1	1	0	1	0	0
m	2,3	15	75	0	0	1	0	0	1	0	1	0	0	0	0	0	0	0	0	0	1	1	0	0	0	0	0	0	0	0	0	5	32	0	0	0	0	0	0	0
w	5,7	12	71	0	0	0	0	0	0	1	1	0	0	0	0	0	0	0	1	0	0	0	0	0	0	0	0	1	0	1	0	13	55	1	0	1	1	0	0	1
m	1,2	15	80	0	0	0	1	0	0	0	0	0	0	0	0	0	0	0	0	0	0	0	0	0	0	0	0	0	0	0	1				0	0	0	0	0	0
w	11,7	6	69	0	0	0	0	0	0	0	0	0	0	0	0	0	0	0	0	1	1	1	1	0	0	0	0	0	0	0	1	19	52	1	0	1	1	0	0	1
m	1,0	15	76	0	0	1	1	0	0	0	0	1	1	0	0	0	0	0	0	0	1	1	0	0	0	0	0	0	0	0	0				0	0	0	0	0	0
m	3,0	12	84	0	0	0	1	0	0	0	0	0	0	0	0	0	0	0	0	0	1	1	0	0	0	0	0	0	0	0	1	5	46	0	1	1	0	0	1	1
m	1,6	15	56	0	0	0	0	0	0	0	0	0	0	0	0	0	0	0	0	0	0	0	0	0	0	0	0	0	0	0	1	0	15	0	0	0	0	1	0	0
w	1,3	15	84	0	0	1	0	1	0	0	1	0	1	0	0	0	0	0	0	0	0	0	0	0	0	0	0	0	0	1	0	0	49	0	0	1	0	1	0	1
w	3,9	12	70	0	0	0	1	0	0	0	1	0	0	0	1	0	0	0	0	0	0	0	0	0	0	0	0	1	1	0	0	12	33	0	1	1	0	1	0	0
m	1,1	15	89	0	0	1	0	1	1	0	0	0	0	0	0	0	0	0	0	0	0	0	0	0	0	0	0	0	0	1	0	5	26	0	0	0	0	0	0	0
w	2,3	15	50	0	0	0	0	0	0	0	0	0	1	0	0	0	1	1	0	0	0	0	0	0	0	0	0	0	0	0	0	17	25	0	0	1	0	0	1	0
w	1,1	15	88	0	0	0	0	0	0	0	0	0	0	0	0	0	0	0	0	0	0	0	0	0	0	0	0	0	0	0	1	5	40	0	0	0	0	0	0	0
m	1,1	15	86	0	0	1	1	0	0	0	0	0	0	0	0	0	0	0	0	0	0	0	0	0	0	0	0	0	0	0	1	0	26	0	0	1	0	0	1	0
m	1,3	15	83	0	0	0	1	0	1	0	0	0	0	1	0	0	0	0	0	0	0	0	0	0	0	0	0	0	0	0	0	5	31	0	0	0	0	1	0	1
m	1,3	15	60	0	0	0	0	0	0	0	1	0	0	0	0	0	0	0	0	0	0	0	0	0	0	0	0	0	0	0	0	0	26	0	0	0	0	0	0	1
m	1,2	15	53	0	0	0	0	0	0	0	0	0	0	0	0	0	0	0	0	0	0	0	0	0	0	0	0	0	0	0	1	0	18	0	0	0	1	0	0	0
w	1,4	15	36	0	0	0	0	0	0	0	0	0	1	0	0	0	0	0	0	0	0	0	0	0	0	0	0	0	0	0	0	0	11	0	0	1	0	0	1	0
m	11,4	6	79	0	0	1	1	0	1	0	1	0	1	0	0	0	0	0	0	0	1	1	0	1	1	0	0	1	1	0	0	24	46	0	1	1	0	0	1	0
m	9,9	6	73	0	0	1	0	0	0	0	1	0	0	0	0	0	1	0	0	1	1	1	0	1	0	1	0	1	0	1	1	14	55	1	1	1	0	0	1	1
w	1,1	15	77	0	0	0	0	0	0	0	0	0	0	0	0	0	0	0	0	0	0	0	0	0	0	0	0	0	0	0	0	5	37	0	0	0	1	0	0	0
w	4,9	12	86	0	0	0	0	0	1	0	1	0	0	0	0	0	0	0	0	0	1	0	0	0	0	0	1	0	0	0	1	10	48	0	0	0	0	0	0	0
m	2,3	15	77	0	0	1	0	0	1	0	0	0	1	0	0	0	0	0	1	0	0	0	0	0	0	0	0	0	0	0	0	10	36	0	0	0	0	0	0	0
m	2,5	15	89	0	0	1	0	0	0	0	0	0	0	0	0	0	0	0	0	0	0	0	0	0	0	0	0	0	0	0	0	5	46	0	0	1	0	0	1	1
m	1,4	15	68	0	0	0	0	0	0	0	0	0	0	0	0	0	0	0	0	0	0	0	0	0	0	0	0	0	0	0	1	5	28	0	0	0	0	0	0	0
m	4,4	12	68	0	0	0	0	0	0	0	0	0	0	0	1	0	0	0	0	0	1	1	0	0	1	0	0	0	0	0	1	19	31	0	0	0	0	0	0	0
w	1,6	15	68	0	0	1	0	0	0	0	0	0	0	0	0	0	0	0	1	0	0	0	0	0	0	0	0	0	0	0	1	15	40	0	0	0	0	0	0	0
w	1,4	15	67	0	0	0	0	1	0	0	0	0	0	0	0	0	0	0	0	0	0	0	0	0	0	0	0	0	0	0	1	0	36	0	0	0	0	0	0	0
m	1,4	15	65	0	0	0	0	1	0	0	0	0	0	0	0	0	0	0	0	0	0	0	0	0	0	0	0	0	0	0	1	0	31	0	0	0	0	0	0	0

m	5,7	12	83	0	0	0	1	1	0	0	1	0	0	0	0	0	0	0	0	0	0	0	0	0	0	0	0	0	0	0	1	0	39	0	1	1	0	0	1	0
m	4,5	12	22	0	0	0	0	0	0	0	0	0	0	0	1	0	0	0	0	1	0	0	0	0	0	0	0	0	0	0	0	15	34	0	0	0	0	0	0	0
m	1,1	15	67	0	0	0	0	0	0	0	0	0	0	0	0	0	0	0	0	0	0	0	0	0	0	0	0	0	0	0	1	0	29	0	0	0	1	0	0	0
w	1,0	15	91	0	0	1	0	1	0	0	0	0	0	0	0	0	0	0	0	0	0	0	0	0	0	0	0	0	0	0	0	5	21	0	0	1	0	1	0	0
w	2,0	15	28	0	0	0	0	0	0	0	0	0	1	0	0	0	0	0	1	0	0	0	0	0	0	0	0	0	0	0	0	5	14	0	0	0	0	0	0	0
m	4,0	12	62	0	0	0	0	0	0	0	0	0	0	1	0	0	0	0	0	1	1	1	1	0	0	0	0	0	0	1	0	15	55	1	0	0	0	0	0	0
m	2,0	15	81	0	0	0	1	0	0	0	0	0	0	0	0	0	0	0	0	0	0	0	0	0	0	0	0	0	0	0	0	0	30	0	1	1	1	0	0	0
m	1,0	15	53	0	0	0	0	0	0	0	0	0	0	0	0	0	0	0	0	0	0	0	0	0	0	0	0	0	0	0	1	0	20	0	0	0	0	0	0	0
m	3,1	12	92	0	0	1	1	0	0	0	0	1	0	0	0	0	0	0	0	1	0	0	0	0	0	0	0	0	0	1	0	5	26	0	0	0	0	1	0	0
m	2,9	15	86	0	0	0	1	0	1	0	1	0	1	0	0	0	0	0	0	1	1	1	0	0	0	0	1	0	0	0	0	14	53	1	0	0	1	0	0	1
w	8,7	9	82	0	0	0	1	1	1	0	0	0	1	0	0	0	0	0	1	0	1	1	0	0	0	0	0	0	0	0	0	19	54	1	0	1	0	0	1	1
w	3,4	12	89	0	0	1	1	0	0	0	0	0	0	0	0	0	0	0	0	0	0	0	0	0	0	0	0	0	0	0	0	14	40	0	1	1	0	1	0	0
w	3,5	12	65	0	0	0	0	0	0	0	0	0	1	0	1	1	0	0	0	0	0	0	0	0	0	1	0	0	0	0	0	0	32	0	0	1	1	0	0	0
w	1,0	15	75	0	0	0	0	1	0	1	0	0	0	0	1	0	0	0	0	0	0	0	0	0	0	0	0	0	0	0	1				0	0	0	0	0	0
m	1,1	15	78	0	0	0	1	0	0	0	0	0	0	0	0	0	0	0	0	0	0	0	0	0	0	0	0	0	0	0	0	0	29	0	0	0	0	0	0	0
m	19,4	0	80	0	0	1	1	0	1	0	1	0	0	1	0	0	0	1	1	0	0	0	0	0	0	0	0	1	1	1	1	22	59	1	1	1	0	0	1	0
w	1,4	15	92	0	0	0	0	0	0	0	1	0	0	0	0	0	0	0	1	0	0	0	0	0	0	0	0	1	1	0	1	8	52	1	0	0	0	0	0	0
m	4,2	12	76	0	0	0	1	0	0	0	1	1	1	0	0	0	0	0	0	0	0	0	0	0	0	0	0	0	0	0	1	5	39	0	0	0	1	0	0	1
m	1,4	15	86	0	0	0	0	0	0	0	1	0	0	0	0	0	0	0	0	0	0	0	0	0	0	0	0	0	0	0	0	10	33	0	0	0	0	0	0	0
m	1,8	15	70	0	0	0	0	0	0	0	0	0	0	0	0	0	0	0	0	0	0	0	0	0	0	0	0	0	0	0	1	5	36	0	0	0	0	0	0	0
m	1,3	15	64	0	0	1	1	0	0	0	1	0	0	0	0	0	0	0	0	0	1	1	1	0	0	0	0	0	0	0	0	10	42	0	0	0	0	0	0	0
m	3,6	12	63	0	0	0	1	1	1	0	0	0	0	0	0	0	0	0	1	0	1	1	0	0	0	0	0	0	0	0	0	10	35	0	0	0	0	0	0	0
m	11,2	6	94	0	0	0	0	0	0	0	0	0	1	0	0	1	0	0	1	0	1	1	0	0	0	0	0	0	0	0	1	5	55	1	1	1	0	0	1	1
m	1,3	15	32	0	0	1	0	0	0	0	0	0	0	0	0	0	0	0	0	0	0	0	0	0	0	0	0	0	0	0	1	5	13	0	0	0	0	0	0	0
w	1,2	15	66	0	0	0	0	0	0	0	0	0	0	0	0	0	0	0	0	0	0	0	0	0	0	0	0	0	0	0	1	0	23	0	0	0	1	0	0	0
m	11,9	6	75	0	0	0	0	1	0	0	1	0	1	1	0	0	0	0	0	0	1	1	1	0	0	0	0	0	0	1	0	4	66	1	1	1	0	0	1	1
m	1,2	15	56	0	0	0	0	0	0	0	0	0	0	0	0	0	1	0	0	1	0	0	0	0	0	0	0	0	0	0	0	10	26	0	0	1	0	1	0	0
m	2,8	15	69	0	0	0	0	0	0	0	0	0	0	0	0	0	0	0	0	0	0	0	0	0	0	0	0	0	0	0	0	5	29	0	0	0	1	0	0	1
w	2,5	15	67	0	0	1	0	0	0	0	0	0	1	0	0	0	0	0	0	0	1	1	0	0	0	0	0	0	0	0	0	5	40	0	0	0	0	0	0	1
m	3,1	12	89	0	0	1	0	1	0	0	0	0	0	0	0	0	0	0	1	0	0	0	0	0	0	0	0	0	0	0	0	0	36	0	1	1	1	0	0	0
m	1,7	15	33	0	0	0	0	0	0	0	0	0	0	0	0	0	1	0	0	0	1	1	0	0	0	0	0	0	0	0	0	9	32	0	0	1	1	0	0	1
m	1,2	15	79	0	0	1	1	0	1	0	0	0	0	0	0	0	0	0	0	0	0	0	0	0	0	0	0	0	0	0	0	5	33	0	0	0	1	0	0	0
m	1,1	15	59	0	0	0	1	0	1	0	0	0	0	0	0	0	0	0	1	0	1	1	0	0	0	0	0	0	0	0	0	10	43	0	0	1	0	0	1	0
w	3,7	12	78	0	0	0	0	0	0	0	0	0	1	0	0	0	0	0	0	1	1	1	0	0	0	0	0	0	0	1	0	24	68	1	0	0	1	0	0	1
m	1,3	15	81	0	0	0	0	0	0	0	0	1	1	0	0	0	0	0	0	0	0	0	0	0	0	0	0	0	0	0	0	0	29	0	0	0	1	0	0	1
m	8,8	9	66	0	0	0	1	0	0	0	0	0	0	0	0	0	0	0	0	1	1	1	0	1	1	0	0	0	0	1	1	19	42	0	0	0	0	0	0	1
w	6,2	9	66	0	0	1	1	0	0	0	1	0	0	0	0	0	0	0	0	0	1	1	1	0	0	0	1	0	0	0	0	9	20	0	1	0	1	0	0	1
m	1,1	15	58	0	0	0	0	0	0	0	0	0	0	0	0	0	1	0	1	0	0	0	0	0	0	0	0	0	0	0	0	5	23	0	0	1	0	1	0	0
m	1,1	15	83	0	0	0	1	0	0	0	0	0	0	0	0	0	0	0	0	0	1	1	0	0	0	0	1	0	0	0	0	9	40	0	0	0	0	0	0	0
m	1,5	15	85	0	0	0	1	0	0	0	0	0	0	0	0	1	0	0	0	0	0	0	0	0	0	0	0	0	0	0	1	5	31	0	0	1	0	1	0	0
w	1,1	15	77	0	0	1	0	0	0	0	0	0	1	1	0	0	0	0	1	0	0	0	0	0	0	0	0	0	0	0	0	10	44	0	0	1	1	0	0	0
w	2,4	15	75	0	0	0	0	0	0	0	0	0	0	0	0	0	0	0	0	0	0	0	0	0	0	0	0	0	0	0	0	0	34	0	0	0	0	0	0	0
w	2,4	15	21	0	0	0	0	0	0	0	0	0	0	1	0	0	0	0	0	0	1	1	0	0	0	0	0	0	0	1	0	0	39	0	0	0	0	0	0	1
m	2,2	15	78	0	0	0	1	1	0	0	1	0	0	0	0	0	0	0	0	0	0	0	0	0	0	0	0	0	0	1	0	0	35	0	1	1	1	0	0	1
w	1,0	15	80	0	0	0	0	0	0	0	0	0	0	0	0	0	0	0	0	0	0	0	0	0	0	0	0	0	0	0	0	5	31	0	0	0	0	0	0	0
w	5,9	12	36	0	0	0	0	0	0	0	0	0	0	0	0	0	0	0	1	0	0	0	0	0	0	0	0	0	0	1	0	10	28	0	0	0	0	1	0	0
m	6,4	9	58	0	0	0	0	0	0	0	0	0	0	0	0	0	0	0	1	1	1	1	0	0	0	0	0	0	0	0	0	10	48	0	1	1	0	0	1	1
m	2,2	15	81	0	0	1	1	0	1	0	0	0	1	1	0	0	0	0	0	0	0	0	0	0	0	0	0	0	0	0	0	9	56	1	0	1	1	0	0	1
w	1,2	15	83	0	0	0	0	0	0	0	0	0	0	0	0	0	0	0	0	0	0	0	0	0	0	0	0	0	0	0	0	0	54	1	0	1	1	0	0	1
m	1,2	15	55	0	0	0	0	0	0	0	0	0	0	0	0	0	0	0	0	0	0	0	0	0	0	0	0	0	0	0	1	0	28	0	0	0	0	0	0	0
m	28,0	0	69	0	0	0	0	0	0	0	0	0	0	0	0	0	0	0	1	1	1	1	0	1	0	0	1	1	0	1	0	31	64	1	1	1	0	0	1	1
w	5,0	12	76	0	0	0	1	0	0	0	1	1	1	0	0	0	0	0	1	0	1	1	0	0	0	0	0	0	0	1	0	10	57	1	0	1	0	0	1	1
m	1,1	15	83	0	0	0	0	0	0	0	0	0	0	0	0	0	0	0	0	0	0	0	0	0	0	0	0	0	0	0	0	5	35	0	0	0	0	0	0	1
m	3,8	12	79	0	0	0	0	0	0	0	0	0	0	0	0	0	0	0	1	0	0	0	0	0	0	0	0	0	0	0	1	15	41	0	1	0	1	0	0	1
m	1,3	15	75	0	0	0	1	0	1	0	1	1	0	0	0	0	0	0	1	0	0	0	0	0	0	0	0	0	0	0	0	10	48	0	0	0	0	0	0	1
w	2,7	15	88	0	0	1	0	0	0	0	0	0	1	0	0	0	0	0	0	0	1	1	0	0	0	0	0	0	0	0	0	5	30	0	1	1	0	0	1	1
w	2,9	15	61	0	0	0	0	0	0	0	1	0	0	0	0	0	0	0	0	0	0	0	0	0	0	0	0	1	1	0	0	7	46	0	0	0	0	0	0	0
m	2,1	15	81	0	0	1	0	1	0	0	0	0	0	0	0	0	0	0	0	0	1	1	0	0	0	0	0	0	0	0	0	9	39	0	0	0	1	0	0	1
w	3,9	12	71	0	0	1	0	0	0	0	0	0	0	1	0	1	0	0	0	0	0	0	0	0	0	0	0	0	0	0	1	0	36	0	1	1	0	1	0	1
m	5,1	12	57	0	0	0	0	0	1	0	0	0	0	1	0	0	0	0	1	1	1	1	0	1	0	0	1	0	0	1	0	20	50	1	0	0	1	0	0	0
m	2,7	15	74	0	0	0	0	0	0	0	0	0	1	0	0	1	0	0	0	0	0	0	0	0	0	0	0	0	0	0	0	10	28	0	1	1	0	0	1	1
m	2,9	15	58	0	0	0	0	0	0	0	0	0	0	0	1	0	1	0	0	0	0	0	0	0	0	0	0	1	0	1	0	7	27	0	0	0	0	0	0	1
m	1,9	15	21	0	0	0	0	0	0	0	0	0	0	0	0	0	0	0	0	0	0	0	0	0	0	0	0	0	0	0	0	13	32	0	0	0	0	0	0	1
w	2,2	15	92	0	0	0	0	0	0	0	0	0	0	0	1	0	0	0	1	0	1	1	0	0	0	0	0	0	0	0	0	15	46	0	0	1	0	0	1	0
m	9,6	6	45	0	0	0	0	1	1	0	0	0	0	0	0	0	0	0	1	1	1	1	0	1	0	1	1	0	0	0	0	27	51	1	0	1	0	1	0	1
w	1,4	15	57	0	0	0	0	0	0	0	0	0	1	0	0	0	0	0	0	1	0	0	0	0	0	0	0	0	0	1	0	15	41	0	0	0	0	0	0	1
m	1,1	15	67	0	0	0	0	0	0	0	0	0	0	0	0	0	0	0	0	0	0	0	0	0	0	0	0	0	0	0	1	0	20	0	0	0	0	0	0	0
m	4,0	12	72	0	0	0	1	0	1	0	1	1	0	0	0	0	0	0	0	0	1	1	0	0	0	0	0	0	0	0	0	5	53	1	0	0	0	0	0	1
m	31,1	0	54	0	0	0	0	0	1	1	0	0	0	0	0	0	1	0	1	1	1	1	1	0	0	0	0	0	0	1	0	15	42	0	1	1	0	0	1	1

w	2,0	15	59	0	0	1	1	1	1	0	0	0	0	0	0	0	0	0	0	0	1	1	0	0	0	0	0	0	0	1	0	5	27	0	0	1	1	0	0	1
m	9,2	6	76	0	0	0	0	0	0	1	0	1	1	0	0	0	0	0	0	0	1	1	0	1	0	0	0	0	0	0	0	10	47	0	1	1	0	0	1	1
m	2,1	15	80	0	0	1	1	0	0	0	1	0	0	0	0	0	0	0	0	0	0	0	0	0	0	0	0	0	0	0	0	5	26	0	0	0	0	0	0	0
m	9,7	6	79	0	0	1	1	0	1	0	1	0	0	0	0	0	0	0	1	0	0	0	0	0	0	0	0	0	0	0	0	10	56	1	1	1	0	0	1	1
m	1,5	15	47	0	0	0	0	1	1	0	0	0	0	0	0	0	0	0	0	0	0	0	0	0	0	0	0	0	0	0	1	5	26	0	0	0	0	0	0	0
m	4,8	12	85	0	0	1	0	0	0	0	0	0	0	1	0	0	0	0	0	1	1	1	0	0	0	0	0	0	0	1	0	10	47	0	1	1	1	0	0	1
m	1,7	15	66	0	0	0	0	0	0	0	0	0	0	0	0	0	0	0	1	0	0	0	0	0	0	0	0	0	0	0	1	10	43	0	0	0	0	0	0	1
m	2,1	15	71	0	0	1	1	0	0	0	0	0	0	0	0	0	0	0	0	0	0	0	0	0	0	0	0	0	0	0	1	0	28	0	0	0	0	0	0	0
m	4,7	12	79	0	0	0	0	0	0	0	0	0	0	0	0	1	1	0	1	0	1	1	0	0	0	0	0	0	0	0	1	15	53	1	1	1	0	0	1	0
w	1,1	15	82	0	0	0	0	0	0	0	0	0	0	0	1	0	0	0	0	0	0	0	0	0	0	0	0	0	0	0	0	5	30	0	0	0	0	0	0	0
w	8,0	9	73	0	0	0	0	0	1	0	1	0	0	0	0	0	1	0	1	1	1	1	0	0	0	0	0	1	0	0	0	15	60	1	0	1	0	1	0	1
m	3,2	12	79	0	0	0	0	1	1	0	0	0	0	1	0	0	0	0	0	0	1	1	0	0	0	0	0	0	0	1	0	0	61	1	0	0	0	0	0	1
w	1,2	15	79	0	0	1	0	0	0	0	0	0	0	0	0	0	0	0	0	0	0	0	0	0	0	0	0	0	0	0	0	10	39	0	0	1	0	1	0	1
m	2,0	15	44	0	0	0	0	0	0	1	0	0	0	0	0	0	1	1	0	0	0	0	0	0	0	0	0	0	0	0	0	4	19	0	0	1	1	0	0	0
m	3,5	12	88	0	0	1	1	0	0	0	0	0	1	1	0	0	0	0	0	0	1	1	0	0	0	0	0	0	0	0	0	5	44	0	1	1	0	1	0	1
m	3,7	12	48	0	0	1	0	0	0	0	0	0	0	0	0	0	0	0	0	1	1	1	0	0	0	0	0	0	0	0	1	0	24	0	0	0	0	0	0	0
m	2,0	15	75	0	0	1	1	1	1	0	1	0	0	0	0	0	0	0	0	1	0	0	0	0	0	0	0	0	0	0	0	15	48	0	0	0	0	0	0	1
m	7,2	9	64	0	0	0	1	1	1	0	0	1	1	0	0	0	0	0	1	0	1	1	0	1	0	0	0	0	0	0	0	14	51	1	1	1	0	0	1	1
m	13,1	3	73	0	0	0	0	1	0	0	1	0	0	0	0	0	0	0	1	0	1	1	0	0	0	0	0	1	0	0	0	13	49	0	1	1	1	0	0	1
m	2,0	15	56	0	0	0	0	0	0	0	0	0	0	0	1	0	0	0	0	1	0	0	0	0	0	0	0	0	0	0	0	10	31	0	1	1	0	0	1	0
w	5,4	12	54	0	0	0	0	1	0	0	0	0	0	0	0	0	0	0	0	1	1	1	1	0	0	0	1	0	0	1	1	19	41	0	1	1	0	0	1	0
w	2,7	15	23	0	0	0	0	0	0	0	0	0	0	0	1	0	0	0	0	1	0	0	0	0	0	0	0	0	0	0	0	14	16	0	0	0	0	0	0	0
m	1,2	15	50	0	0	0	0	0	0	0	0	0	0	0	0	0	0	0	0	0	0	0	0	0	0	0	0	0	0	0	0	0	16	0	0	0	1	0	0	1
w	1,2	15	55	0	0	0	0	0	0	1	0	0	0	0	0	0	0	0	0	0	0	0	0	0	0	0	0	0	0	0	0	10	28	0	0	1	1	0	0	1
m	1,8	15	40	0	0	0	0	0	0	0	0	0	0	0	0	0	0	0	1	0	0	0	0	0	0	0	0	0	0	0	1	15	24	0	0	0	0	0	0	0
w	2,0	15	53	0	0	0	0	0	0	0	0	0	0	0	0	0	0	0	0	0	1	1	1	0	0	0	0	0	0	0	0	5	34	0	0	1	0	0	1	1
w	1,4	15	66	0	0	0	0	1	1	0	0	0	0	0	0	0	0	0	0	0	0	0	0	0	0	0	0	0	0	0	1	0	24	0	0	0	1	0	0	1
m	4,5	12	75	0	0	0	0	0	0	0	0	0	0	0	0	0	0	0	0	0	1	1	0	0	0	0	0	0	0	0	0	14	60	1	1	1	0	1	0	1
m	12,8	3	82	0	0	0	1	0	0	0	0	0	1	0	0	1	0	0	0	1	1	1	0	0	0	0	0	0	0	1	1	15	57	1	0	1	0	0	1	0
w	1,0	15	93	0	0	1	0	0	0	0	0	0	1	1	0	0	0	0	0	0	1	1	0	0	0	0	0	0	0	1	0				0	0	0	0	0	0
w	1,0	15	63	0	0	0	0	0	0	0	0	0	0	0	0	0	0	0	0	0	0	0	0	0	0	0	0	0	0	0	1	0	20	0	0	0	0	0	0	0
m	1,3	15	59	0	0	1	1	0	1	0	0	0	0	0	0	0	0	0	0	0	0	0	0	0	0	0	0	0	0	0	1	5	15	0	0	0	1	0	0	0
w	3,2	12	37	0	0	0	0	0	0	0	0	0	0	0	0	0	0	0	0	0	1	1	1	0	0	0	0	0	0	0	0	9	32	0	0	0	0	0	0	1
m	1,6	15	32	0	0	0	0	0	1	0	0	0	0	1	0	0	0	0	0	0	0	0	0	0	0	0	0	0	0	0	0	5	8	0	0	0	0	0	0	1
w	1,4	15	80	0	0	0	0	0	0	0	0	0	1	1	0	0	0	0	0	0	0	0	0	0	0	0	0	0	0	0	0	9	57	1	0	0	0	0	0	1
m	1,1	15	80	0	0	1	1	1	0	0	0	1	0	0	0	0	0	0	0	0	0	0	0	0	0	0	0	0	0	0	1	0	33	0	0	0	0	0	0	0
m	1,8	15	81	0	0	1	1	0	0	0	0	0	0	0	0	0	0	0	0	0	0	0	0	0	0	0	0	0	0	0	0	5	26	0	0	0	0	0	0	1
m	4,8	12	67	0	0	0	0	0	0	0	1	0	1	1	0	1	0	0	1	0	0	0	0	0	0	0	0	0	0	0	0	5	32	0	0	0	0	0	0	0
m	5,6	12	64	0	0	0	0	0	1	0	0	0	0	0	1	0	0	0	1	1	1	1	0	0	0	0	0	0	0	0	0	15	37	0	1	1	0	0	1	0
m	2,8	15	73	0	0	0	0	0	0	0	0	0	0	0	0	0	0	0	0	0	0	0	0	0	0	0	0	0	0	0	1	5	28	0	0	1	1	0	0	0
m	1,5	15	84	0	0	0	0	0	0	0	0	0	0	0	0	0	0	0	0	0	0	0	0	0	0	0	0	0	0	0	1	0	31	0	0	0	0	1	0	0
m	1,3	15	68	0	0	0	0	0	0	0	0	0	0	0	0	0	0	0	0	0	0	0	0	0	0	0	0	0	0	0	1	0	24	0	0	0	0	0	0	0
w	1,3	15	71	0	0	0	0	1	0	0	0	0	0	0	0	0	0	0	1	0	0	0	0	0	0	0	0	0	0	0	0	10	34	0	0	1	1	0	0	0
m	2,2	15	81	0	0	0	0	1	1	0	1	1	0	0	0	0	0	0	0	1	1	1	0	0	0	0	0	0	0	0	1	15	43	0	1	1	0	1	0	0
w	1,7	15	85	0	0	0	0	1	0	0	0	0	0	0	0	0	0	0	0	0	0	0	0	0	0	0	0	0	0	0	0	5	36	0	0	1	1	0	0	0
m	2,7	15	61	0	0	0	0	0	0	0	0	0	0	0	0	0	0	0	0	0	0	0	0	0	0	0	0	0	0	0	0	5	21	0	1	1	0	0	1	0
m	1,3	15	88	0	0	1	0	0	0	0	0	0	0	0	0	0	0	0	0	0	0	0	0	0	0	0	0	0	0	0	1	5	45	0	0	1	0	1	0	0
w	3,0	12	70	0	0	0	0	0	0	0	0	0	0	0	0	0	0	0	0	0	0	0	0	0	0	0	0	0	0	0	0	9	33	0	1	1	0	0	1	0
m	2,9	15	61	0	0	0	1	0	1	0	1	1	1	0	0	0	0	0	1	1	1	1	0	0	0	0	0	0	0	0	1	10	50	1	0	0	0	0	0	1
m	1,7	15	80	0	0	1	1	0	0	0	0	1	0	0	0	0	0	0	0	0	0	0	0	0	0	0	0	0	0	0	0	5	56	1	0	0	0	0	0	1
m	2,8	15	59	0	0	0	0	0	0	0	0	0	0	1	0	0	0	0	0	0	0	0	0	0	0	0	0	0	0	0	0	5	22	0	0	1	0	1	0	0
m	7,9	9	44	0	0	0	0	1	0	0	0	0	1	0	0	0	0	0	0	1	1	1	0	1	0	0	0	0	0	0	1	15	56	1	0	1	0	0	1	1
w	1,2	15	88	0	0	0	0	0	0	0	0	1	1	0	0	0	0	0	0	0	0	0	0	0	0	0	0	0	0	0	1	5	45	0	0	0	0	0	0	0
m	1,1	15	84	0	0	0	1	0	0	0	0	0	1	0	0	0	0	0	0	0	0	0	0	0	0	0	0	0	0	0	0	5	20	0	0	0	0	0	0	0
w	14,0	3	66	0	0	1	0	1	0	0	1	0	0	0	0	0	0	0	1	1	1	1	0	0	0	0	0	1	0	1	0	15	65	1	0	1	0	0	1	1
m	1,6	15	54	0	0	0	1	0	0	0	0	0	1	0	1	0	0	1	0	0	0	0	0	0	0	0	0	0	0	0	0	5	24	0	0	0	0	0	0	1
m	1,2	15	66	0	0	0	1	0	1	0	1	0	0	0	0	0	0	0	1	0	0	0	0	0	0	0	0	0	0	0	1	10	26	0	0	0	0	0	0	0
w	1,0	15	84	0	0	1	0	0	0	0	0	0	0	0	0	0	0	0	0	0	0	0	0	0	0	0	0	0	0	0	1	0	26	0	0	0	1	0	0	0
m	2,5	15	70	0	0	0	0	0	0	0	0	0	0	0	0	0	0	0	0	0	0	0	0	0	0	0	0	0	0	1	1	10	42	0	0	0	0	0	0	0
m	1,9	15	62	0	0	0	0	0	0	1	0	0	0	0	0	0	0	0	1	1	1	1	1	0	0	0	0	0	0	0	0	10	40	0	1	1	0	1	0	0
w	1,1	15	92	0	0	1	0	0	0	0	0	0	0	0	0	0	0	0	0	0	0	0	0	0	0	0	0	0	0	0	0	5	31	0	0	0	0	0	0	0
w	3,0	12	92	0	0	0	1	0	0	0	0	0	0	0	0	0	0	0	1	1	0	0	0	0	0	0	0	0	0	0	1	5	30	0	0	0	1	0	0	0
m	7,0	9	88	0	0	1	1	0	1	0	1	0	1	0	0	0	0	0	1	1	1	0	0	0	0	0	0	0	0	0	1	10	46	0	1	1	0	1	0	0
m	1,1	15	68	0	0	0	1	0	0	0	0	0	0	0	0	0	0	0	0	0	0	0	0	0	0	0	0	0	0	0	1	5	25	0	0	0	0	0	0	0
m	1,1	15	53	0	0	0	0	0	0	0	0	0	0	0	0	0	0	0	0	0	0	0	0	0	0	0	0	0	0	0	1	0	13	0	0	0	0	0	0	0
m	1,2	15	80	0	0	0	1	0	0	0	0	0	0	0	0	0	0	0	0	0	0	0	0	0	0	0	0	0	0	1	0	5	56	1	0	1	0	1	0	0
m	1,7	15	55	0	0	0	0	0	0	0	0	0	0	0	0	0	0	0	0	0	0	0	0	0	0	0	0	0	0	0	1	0	17	0	0	0	1	0	0	0
m	1,0	15	74	0	0	0	0	0	0	0	0	0	0	0	0	0	0	0	0	0	0	0	0	0	0	0	0	0	0	0	1	0	23	0	0	0	0	0	0	0
w	1,7	15	69	0	0	0	0	0	0	0	0	0	0	0	0	0	0	0	0	0	0	0	0	0	0	0	0	0	0	0	1	10	33	0	0	0	0	0	0	0

m	1,1	15	53	0	0	0	0	0	0	1	1	0	0	0	0	0	0	1	1	0	0	0	0	0	0	0	0	0	0	0	0	5	42	0	1	1	1	0	0	0
w	6,6	9	81	0	0	1	0	0	0	0	0	0	1	0	1	0	0	0	1	0	1	1	0	0	0	0	0	0	0	0	0	10	44	0	1	1	0	0	1	1
m	2,3	15	69	0	0	0	0	0	0	0	0	0	0	1	0	0	0	0	0	0	0	0	0	0	0	0	0	0	0	0	0	10	35	0	1	1	0	0	1	0
m	32,4	0	64	0	0	1	0	0	0	0	0	0	0	0	0	0	1	0	0	1	1	1	0	1	0	1	0	0	0	1	1	27	45	0	1	1	0	0	1	0
m	1,6	15	70	0	0	0	0	0	0	0	0	0	0	1	0	1	0	0	0	0	1	1	0	0	0	0	0	0	0	1	0	5	46	0	0	0	0	0	0	0
m	1,2	15	58	0	0	0	1	0	0	0	0	0	0	0	0	0	0	0	0	0	0	0	0	0	0	0	0	0	0	0	1	0	22	0	0	0	0	0	0	0
m	5,9	12	48	0	0	0	0	0	0	0	0	0	0	1	0	0	0	0	1	1	1	1	0	0	0	0	0	0	0	1	0	15	48	0	0	0	0	0	0	1
m	1,9	15	97	0	0	0	1	0	0	0	0	0	0	0	0	0	0	0	0	0	0	0	0	0	0	0	0	0	0	0	1	5	57	1	0	1	1	0	0	0
m	1,9	15	58	0	0	0	0	1	0	0	0	0	0	0	0	0	0	0	0	0	0	0	0	0	0	0	0	0	0	0	1	5	19	0	0	0	1	0	0	0
m	16,0	0	70	0	0	1	0	0	0	0	0	0	0	0	1	0	0	0	1	1	1	1	1	0	0	0	0	0	0	1	0	15	54	1	0	1	0	0	1	1
m	1,8	15	81	0	0	1	1	0	0	0	1	0	0	0	0	0	0	0	0	0	0	0	0	0	0	0	0	0	0	1	0	10	35	0	0	0	0	0	0	0
m	5,7	12	86	0	0	1	1	0	1	0	0	1	1	0	0	1	1	0	1	1	1	1	0	0	0	0	0	0	0	0	0	10	48	0	1	1	0	1	0	1
w	1,2	15	47	0	0	0	0	0	0	0	0	0	0	1	0	0	0	0	0	0	0	0	0	0	0	0	0	0	0	0	0	5	28	0	0	0	0	0	0	0
m	1,4	15	75	0	0	0	0	0	0	0	0	0	1	1	1	0	0	0	0	0	0	0	0	0	0	0	0	0	0	1	0	10	46	0	0	1	0	1	0	1
m	1,6	15	70	0	0	0	1	0	0	0	1	0	0	0	0	0	0	0	0	0	0	0	0	0	0	0	0	0	0	0	0	5	54	1	0	1	0	1	0	1
w	3,9	12	60	0	0	0	0	0	0	0	0	0	1	0	0	0	0	0	0	0	1	1	0	0	0	0	0	0	0	0	0	9	35	0	0	1	1	0	0	0
m	1,5	15	68	0	0	0	0	0	0	0	0	1	1	0	0	0	1	0	1	0	0	0	0	0	0	0	0	0	0	0	1	15	45	0	1	0	0	0	1	1
m	1,2	15	63	0	0	1	0	0	0	0	0	0	1	0	0	0	0	0	0	0	0	0	0	0	0	0	0	0	0	0	0	5	35	0	0	0	1	0	0	0
m	12,8	3	56	0	0	1	1	0	0	0	1	0	0	0	0	0	0	0	1	1	1	1	0	0	0	0	1	0	0	1	1	19	39	0	1	1	1	0	0	1
w	6,9	9	89	0	0	1	1	0	0	0	0	0	0	0	0	0	0	0	0	1	1	1	0	0	0	0	0	0	0	1	0	10	39	0	1	1	0	0	1	0
m	13,3	3	52	0	0	0	0	1	1	0	0	0	0	0	1	0	0	0	1	1	0	0	0	0	0	0	0	0	0	0	0	10	30	0	1	1	1	0	0	1
m	6,1	9	67	0	0	0	0	0	0	0	0	0	1	0	0	1	0	0	1	0	0	0	0	0	0	0	0	0	0	0	0	5	25	0	1	1	1	0	0	0
m	1,4	15	65	0	0	0	1	0	0	0	0	0	0	0	0	0	0	0	0	0	0	0	0	0	0	0	0	0	0	0	1	10	42	0	0	0	1	0	0	0
w	1,0	15	75	0	0	0	1	0	1	0	0	0	0	0	0	0	0	0	0	0	0	0	0	0	0	0	0	0	0	0	1	5	37	0	0	0	0	0	0	0
m	2,8	15	90	0	0	0	0	0	0	0	0	1	0	0	0	0	0	0	0	0	0	0	0	0	0	0	0	0	0	0	1	5	34	0	1	1	0	0	1	0
m	1,6	15	85	0	0	1	0	0	1	0	1	0	0	0	0	0	0	0	1	0	0	0	0	0	0	0	0	0	0	0	0	15	56	1	0	0	1	0	0	0
w	1,0	15	31	0	0	0	0	0	0	0	0	0	0	0	1	0	0	0	0	0	0	0	0	0	0	0	0	0	0	0	0				0	0	1	0	0	0
m	1,3	15	52	0	0	0	0	0	1	0	0	0	0	0	0	0	0	0	1	0	0	0	0	0	0	0	0	0	0	0	0	10	44	0	0	0	0	0	0	0
m	1,3	15	80	0	0	0	1	1	0	0	0	0	0	0	0	0	0	0	0	0	0	0	0	0	0	0	0	0	0	0	0	5	48	0	0	0	1	0	0	0
w	2,7	15	52	0	0	1	0	1	0	0	1	0	0	0	0	0	0	0	0	1	1	1	0	0	0	0	0	0	0	0	0	15	31	0	0	1	0	0	1	1
m	1,1	15	84	0	0	1	0	0	0	0	0	0	1	0	0	0	0	0	0	0	1	1	0	0	0	0	0	0	0	1	0	0	39	0	0	0	0	0	0	0
m	1,7	15	78	0	0	0	0	0	0	0	0	0	0	0	0	0	0	0	0	1	0	0	0	0	0	0	0	0	0	0	0	15	36	0	1	1	0	0	1	0
m	1,9	15	73	0	0	1	1	0	1	0	0	0	0	0	0	0	0	0	1	0	0	0	0	0	0	0	0	0	0	0	0	10	49	0	0	0	0	0	0	1
m	9,2	6	55	0	0	1	0	0	0	0	0	0	1	0	0	0	0	0	0	0	0	0	0	0	0	0	0	0	0	0	0	0	15	0	0	0	1	0	0	0
m	1,5	15	67	0	0	0	0	0	0	0	0	0	1	0	0	0	0	0	0	0	0	0	0	0	0	0	0	0	0	0	1	0	25	0	0	0	0	0	0	0
w	1,1	15	57	0	0	0	0	0	0	0	0	0	0	1	0	0	0	0	0	0	0	0	0	0	0	0	0	0	0	0	0	0	29	0	0	0	0	0	0	0
m	1,6	15	54	0	0	0	1	0	1	0	0	0	0	0	0	0	0	0	0	0	0	0	0	0	0	0	0	0	0	0	1	5	37	0	0	0	0	0	0	1
m	6,4	9	58	0	0	0	0	0	0	0	0	0	0	0	0	0	0	0	0	0	1	1	0	0	0	0	0	0	0	1	0	14	34	0	0	0	0	0	0	1
w	1,6	15	68	0	0	0	1	1	0	0	1	1	0	0	0	0	0	0	0	0	0	0	0	0	0	0	0	0	0	0	0	4	40	0	0	0	1	0	0	1
m	2,8	15	81	0	0	1	1	0	0	0	0	0	0	0	0	0	0	0	0	0	0	0	0	0	0	0	0	0	0	0	0	5	30	0	1	1	0	0	1	1
m	6,0	9	64	0	0	0	1	0	0	1	0	1	1	1	0	0	0	0	0	0	1	1	0	0	0	0	0	0	0	0	0	10	42	0	1	1	0	1	0	1
m	1,1	15	30	0	0	0	0	0	0	0	0	0	0	0	0	0	0	0	0	0	0	0	0	0	0	0	0	0	0	0	1	5	13	0	0	0	0	0	0	0
w	8,0	9	57	0	0	0	0	0	0	0	0	0	0	0	0	0	0	0	0	1	1	1	0	0	0	0	0	0	0	0	1	15	29	0	0	1	1	0	0	0
m	2,0	15	84	0	0	0	1	1	0	0	0	0	0	1	0	0	0	0	0	0	1	1	0	0	0	0	0	0	0	0	1	5	29	0	0	1	1	0	0	0
m	1,9	15	83	0	0	1	1	0	0	0	1	1	0	0	0	0	0	0	0	0	0	0	0	0	0	0	0	0	0	0	1	5	45	0	0	0	0	0	0	0
m	2,6	15	69	0	0	0	1	0	1	1	0	0	0	0	1	0	1	0	0	0	1	1	0	0	0	0	0	0	0	0	0	5	51	1	0	0	0	0	0	1
m	6,8	9	75	0	0	1	0	0	0	0	0	0	1	0	0	1	0	0	0	0	0	0	0	0	0	0	0	0	0	0	1	5	34	0	0	1	0	0	1	1
m	1,2	15	69	0	0	0	0	0	1	0	0	0	0	0	0	0	0	0	0	1	1	1	0	0	0	0	0	0	0	0	0	10	60	1	0	0	0	0	0	0
m	3,8	12	77	0	0	1	1	1	0	0	1	0	1	0	0	0	0	0	0	0	0	0	0	0	0	0	0	0	0	0	1	0	30	0	0	0	0	0	0	1
m	1,8	15	77	0	0	0	0	0	0	0	0	1	0	0	0	0	0	0	0	0	0	0	0	0	0	0	0	0	0	0	1	5	40	0	0	0	0	0	0	0
m	1,9	15	66	0	0	1	0	1	0	0	0	0	0	0	0	0	0	0	0	0	0	0	0	0	0	0	0	0	0	0	1	5	33	0	0	0	1	0	0	1
w	4,4	12	75	0	0	0	0	0	0	0	0	1	0	0	0	0	0	0	0	0	0	0	0	0	0	0	1	0	0	0	1	10	34	0	0	0	0	0	0	0
m	4,0	12	46	0	0	0	0	0	0	0	0	0	1	0	0	0	0	0	0	0	0	0	0	0	0	0	0	0	0	0	0	5	30	0	0	0	0	0	0	0
w	1,8	15	64	0	0	0	0	1	0	0	0	0	0	0	1	0	1	0	0	0	0	0	0	0	0	0	0	1	0	0	0	0	42	0	0	1	1	0	0	1
m	3,4	12	70	0	0	0	0	0	0	0	0	0	0	0	0	0	1	0	1	0	1	1	0	0	0	0	0	0	0	0	1	15	55	1	0	1	1	0	0	0
w	6,2	9	86	0	0	0	1	0	1	0	0	1	1	0	0	0	0	0	1	0	0	0	0	0	0	0	0	0	0	0	0	10	43	0	0	0	0	0	0	1
m	1,5	15	89	0	0	0	0	0	0	0	0	0	0	0	0	0	0	0	0	0	0	0	0	0	0	0	0	0	0	0	0	5	53	1	0	0	0	0	0	0
m	1,9	15	73	0	0	0	1	1	0	0	0	1	0	0	0	0	1	0	1	0	0	0	0	0	0	0	0	0	0	0	0	5	24	0	0	1	0	1	0	0
m	1,9	15	73	0	0	0	1	0	0	0	0	1	1	0	0	0	0	0	0	0	0	0	0	0	0	0	0	0	0	0	1	0	23	0	0	0	0	0	0	0
m	1,3	15	79	0	0	0	1	0	0	0	0	1	0	0	0	0	0	0	0	0	0	0	0	0	0	0	0	0	0	0	1	0	35	0	0	0	0	0	0	0
m	1,0	15	82	0	0	0	1	1	0	0	1	0	0	0	0	0	0	0	0	0	0	0	0	0	0	0	0	0	0	0	0				0	0	0	0	0	1
m	1,5	15	87	0	0	0	0	0	0	0	0	0	1	0	0	0	0	0	1	1	1	1	0	0	0	0	0	0	0	0	0	15	53	1	0	0	0	0	0	0
m	10,7	6	58	0	0	0	0	0	0	0	0	0	0	0	0	0	0	0	1	1	1	1	0	1	0	0	0	0	0	1	1	15	39	0	1	1	0	1	0	0
w	1,0	15	85	0	0	0	0	0	0	0	0	0	0	0	0	0	0	0	0	0	0	0	0	0	0	0	0	0	0	0	0	5	39	0	0	0	0	0	0	0
m	5,9	12	63	0	0	1	1	1	0	0	0	1	0	0	0	0	0	0	1	1	0	0	0	0	0	0	0	0	0	0	0	15	43	0	0	1	0	1	0	1
m	3,0	12	56	0	0	0	1	0	0	0	0	0	0	0	0	0	0	0	0	1	0	0	0	0	0	0	0	0	0	0	0	10	40	0	0	1	1	0	0	0
w	1,4	15	78	0	0	0	0	0	1	0	0	0	0	0	0	0	0	0	0	0	0	0	0	0	0	0	0	0	0	0	1	5	23	0	0	0	0	0	0	0
m	9,7	6	50	0	0	1	0	0	0	1	1	0	0	0	0	0	1	1	1	0	1	1	0	0	0	0	0	0	0	1	1	5	31	0	1	1	0	0	1	1
m	4,5	12	82	0	0	1	0	0	0	0	0	0	1	0	0	0	0	0	0	0	1	1	0	0	0	0	0	0	0	0	0	10	38	0	0	1	0	0	1	1

w	2,9	15	85	0	0	1	1	0	0	0	1	0	0	0	0	0	0	0	0	0	1	1	1	0	0	0	0	1	1	1	0	8	53	1	1	1	0	0	1	0
m	1,1	15	48	0	0	0	0	1	1	0	0	0	0	0	0	0	0	0	0	0	0	0	0	0	0	0	0	0	0	0	0	0	15	0	0	0	0	0	0	0
m	8,8	9	70	0	0	0	0	0	0	0	1	0	0	0	0	0	0	0	1	0	1	1	0	1	0	0	0	0	0	1	0	14	62	1	0	0	0	0	0	1
m	1,2	15	74	0	0	0	1	0	1	0	1	0	0	0	0	0	0	0	0	0	0	0	0	0	0	0	0	1	0	0	0	8	44	0	0	0	0	0	0	1
m	12,0	3	71	0	0	0	1	0	0	0	0	0	1	0	0	0	0	0	0	1	1	1	0	0	0	0	0	0	0	1	0	10	42	0	1	1	0	0	1	0
m	1,2	15	78	0	0	0	0	0	0	1	0	0	0	0	0	0	0	0	0	1	0	0	0	0	0	0	0	0	0	1	0	15	50	1	0	0	0	0	0	0
m	1,1	15	69	0	0	1	1	0	1	0	1	1	1	0	0	0	0	0	0	0	0	0	0	0	0	0	0	0	0	0	0	0	37	0	0	0	1	0	0	1
m	1,4	15	87	0	0	0	1	1	0	0	0	0	0	0	0	0	0	0	0	0	0	0	0	0	0	0	0	0	0	0	1	5	39	0	0	0	0	0	0	0
m	2,3	15	55	0	0	0	0	0	1	1	0	0	0	0	0	0	0	0	1	0	0	0	0	0	0	0	0	0	0	0	0	10	39	0	0	0	0	0	0	1
w	1,3	15	47	0	0	0	0	0	0	0	0	0	0	0	0	0	0	0	0	0	0	0	0	0	0	0	0	0	0	0	1	5	18	0	0	0	0	0	0	0
m	1,8	15	55	0	0	0	0	0	0	0	0	0	0	0	0	0	0	0	1	0	0	0	0	0	0	0	0	0	0	0	0	10	30	0	0	0	0	0	0	0
m	3,8	12	77	0	0	0	1	0	1	0	0	0	0	0	0	0	0	0	1	0	1	1	0	0	0	0	0	0	0	0	0	10	35	0	0	1	0	1	0	0
m	3,5	12	76	0	0	1	1	0	0	0	0	0	1	0	0	0	0	0	0	0	1	1	0	0	0	0	0	0	0	0	0	10	54	1	0	1	0	1	0	1
m	1,7	15	46	0	0	1	0	0	0	1	0	0	0	0	0	0	1	0	0	1	0	0	0	0	0	0	0	0	0	1	0	10	33	0	0	1	0	0	1	0
w	1,8	15	64	0	0	0	1	0	1	0	0	0	1	0	1	0	0	0	0	0	0	0	0	0	0	0	0	0	0	0	0	5	25	0	0	0	0	0	0	0
w	2,8	15	83	0	0	0	0	0	0	0	0	0	0	0	0	0	0	0	0	0	0	0	0	0	0	0	0	0	0	0	1	10	28	0	1	1	0	1	0	1
m	1,1	15	80	0	0	0	1	0	0	0	0	0	0	0	0	0	1	0	0	0	0	0	0	0	0	0	0	0	0	1	0	9	38	0	0	0	0	0	0	0
m	5,7	12	68	0	0	0	1	0	1	0	0	0	0	0	0	0	0	0	1	1	1	1	1	0	0	0	0	0	0	0	0	14	52	1	0	1	1	0	0	1
m	9,9	6	61	0	0	0	0	0	1	0	0	0	0	0	0	0	0	0	1	1	1	1	0	1	0	0	0	0	0	0	0	19	57	1	1	1	0	0	1	1
m	1,1	15	78	0	0	0	0	1	0	0	0	0	0	0	0	0	0	0	0	0	0	0	0	0	0	0	0	0	0	0	0	5	41	0	0	0	0	0	0	0
m	1,3	15	80	0	0	1	1	1	1	0	1	0	1	0	0	0	0	0	0	0	0	0	0	0	0	0	0	0	0	0	0	0	54	1	0	1	0	0	1	1
m	1,2	15	54	0	0	0	0	0	1	0	0	0	0	0	0	0	0	0	1	1	1	1	1	0	0	0	0	0	0	0	0	10	34	0	1	0	1	0	0	0
w	1,4	15	36	0	0	0	0	0	0	0	0	0	0	0	1	0	0	1	0	1	0	0	0	0	0	0	0	0	0	0	0	10	19	0	0	0	0	0	1	0
w	1,0	15	81	0	0	1	0	0	0	0	0	0	0	0	0	0	0	0	0	0	0	0	0	0	0	0	0	0	0	0	0	10	31	0	0	0	0	0	0	0
w	4,1	12	74	0	0	0	1	0	0	1	0	0	1	0	0	0	0	1	0	0	1	1	0	0	0	0	0	0	0	1	0	13	48	0	0	1	0	1	0	1
m	1,4	15	58	0	0	0	0	0	0	0	0	0	0	0	0	0	0	0	0	0	0	0	0	0	0	0	0	0	0	0	1	0	24	0	0	0	1	0	0	0
w	2,9	15	50	0	0	0	0	0	0	0	0	0	1	1	0	0	0	0	0	0	1	1	0	1	0	0	0	0	0	0	0	14	52	1	0	1	1	0	0	1
m	2,8	15	57	0	0	0	0	0	0	0	0	0	0	0	0	0	0	0	1	0	1	1	1	0	0	0	0	0	0	0	0	14	27	0	0	0	0	0	0	1
w	1,1	15	69	0	0	0	0	1	0	0	0	0	1	1	0	0	1	0	0	0	0	0	0	0	0	0	0	0	0	1	0	10	40	0	0	0	0	0	0	0
w	1,0	15	48	0	0	0	0	0	0	0	0	0	0	0	0	0	0	0	0	0	0	0	0	0	0	0	0	0	0	0	1	10	25	0	0	1	0	0	1	0
m	1,7	15	57	0	0	0	0	0	0	0	1	0	0	0	1	0	0	0	0	0	0	0	0	0	0	0	0	0	0	1	0	9	21	0	0	0	0	0	0	1
m	4,8	12	64	0	0	1	0	0	1	0	0	0	1	0	0	0	0	0	1	0	1	1	0	0	0	0	0	0	0	0	0	15	35	0	1	1	0	0	1	0
m	2,3	15	73	0	0	0	1	0	0	0	0	0	1	0	0	0	0	0	0	0	0	0	0	0	0	0	0	0	0	0	0	5	23	0	0	0	0	0	0	0
w	1,4	15	91	0	0	1	1	1	0	0	0	1	0	0	0	0	0	0	0	0	0	0	0	0	0	0	0	0	0	0	1	4	38	0	0	0	0	0	0	0
m	2,0	15	63	0	0	0	0	0	0	0	0	0	0	0	0	0	0	0	0	0	0	0	0	0	0	0	0	0	0	0	1	5	20	0	0	0	0	0	0	0
w	1,2	15	83	0	0	0	0	0	0	0	0	0	0	1	1	1	0	0	0	0	0	0	0	0	0	0	0	0	0	0	0	5	37	0	0	1	1	0	0	0
w	2,6	15	79	0	0	0	1	0	1	0	1	0	0	0	0	0	0	0	1	0	0	0	0	0	0	0	0	0	0	0	0	10	34	0	0	0	0	0	0	0
m	3,2	12	55	0	0	0	1	0	0	0	0	0	0	0	0	0	0	0	0	0	0	0	0	0	0	0	0	0	0	0	1	5	20	0	0	0	0	0	0	0
m	1,0	15	81	0	0	0	1	0	0	0	0	0	0	1	0	0	0	0	0	0	0	0	0	0	0	0	0	0	0	0	0	5	41	0	0	0	1	0	0	0
w	1,2	15	84	0	0	1	0	0	0	0	0	0	1	0	0	0	0	0	0	0	0	0	0	0	0	0	0	0	0	0	0	5	50	1	1	1	0	1	0	0
m	1,1	15	70	0	0	0	1	1	0	0	0	0	1	0	0	0	0	0	0	0	0	0	0	0	0	0	0	0	0	0	1	0	28	0	0	0	0	0	0	0
w	1,9	15	57	0	0	0	0	0	0	0	0	0	0	1	0	0	0	0	0	0	1	1	0	0	0	0	0	0	0	0	0	10	61	1	0	0	0	0	0	1
w	1,2	15	60	0	0	0	0	0	0	1	1	0	0	0	0	0	0	0	0	0	0	0	0	0	0	0	0	0	0	0	0	0	26	0	0	0	0	0	0	1
m	1,0	15	57	0	0	0	1	0	0	0	1	0	0	0	0	0	0	0	0	0	0	0	0	0	0	0	0	0	0	0	1	0	17	0	0	0	0	0	0	0
m	2,9	15	80	0	0	1	1	0	0	0	0	0	1	0	0	0	1	0	0	0	0	0	0	0	0	0	0	0	0	0	0	5	31	0	0	0	1	0	0	0
m	5,6	12	39	0	0	0	0	0	0	0	0	0	0	0	0	0	1	1	0	1	1	1	0	0	0	0	0	0	0	0	1	10	31	0	1	1	0	0	1	0
m	2,5	15	73	0	0	0	0	0	0	0	0	0	0	0	0	0	0	0	0	0	1	1	0	0	0	0	0	0	0	0	0	13	40	0	0	0	0	0	0	1
m	3,0	12	56	0	0	1	0	0	0	0	0	0	0	0	0	0	1	0	0	1	1	1	1	0	0	0	0	0	0	0	0	10	51	1	0	1	1	0	0	0
m	2,1	15	72	0	0	0	0	0	0	0	0	1	1	0	0	0	1	0	1	0	0	0	0	0	0	0	0	0	0	0	0	10	41	0	0	1	0	0	1	0
w	4,5	12	86	0	0	0	0	0	0	0	0	0	0	0	0	0	0	0	1	1	1	1	1	0	0	0	0	0	0	0	1	15	51	1	0	1	1	0	0	1
m	1,9	15	85	0	0	1	0	0	0	0	0	0	1	0	0	0	0	0	0	0	0	0	0	0	0	0	0	0	0	0	1	5	30	0	1	1	1	0	0	0
w	1,6	15	70	0	0	0	0	0	0	0	0	0	0	0	1	0	0	0	0	0	0	0	0	0	0	0	0	0	0	0	0	9	30	0	0	1	0	1	0	0
w	1,3	15	84	0	0	0	0	0	0	0	0	0	0	0	0	0	0	0	0	0	0	0	0	0	0	0	0	0	0	0	1	0	34	0	0	0	0	0	0	1
w	3,7	12	77	0	0	0	0	0	0	0	0	0	0	0	0	0	0	0	0	0	1	1	0	0	0	0	0	0	0	0	1	5	29	0	0	0	0	0	0	0
w	2,3	15	48	0	0	0	0	0	0	0	0	0	1	0	0	0	0	0	0	0	0	0	0	0	0	0	0	0	0	0	0	0	31	0	0	1	1	0	0	1
w	5,9	12	29	0	0	0	0	1	0	0	0	0	1	0	0	0	0	0	0	0	0	0	0	0	0	0	0	0	0	0	0	0	10	0	0	1	1	0	0	1
m	1,9	15	71	0	0	0	0	1	0	0	0	0	0	0	0	0	0	0	0	0	0	0	0	0	0	0	0	0	0	0	1	0	23	0	0	0	0	0	0	0
m	2,1	15	78	0	0	1	1	0	1	0	0	0	0	0	0	0	0	0	1	0	0	0	0	0	0	0	0	0	0	0	0	10	50	1	1	1	0	1	0	1
w	1,6	15	69	0	0	0	0	0	0	0	0	0	0	0	0	0	0	0	0	0	0	0	0	0	0	0	0	0	0	1	0	5	29	0	0	0	0	0	0	0
m	1,0	15	79	0	0	1	1	1	0	0	0	0	0	0	0	0	0	0	0	0	1	1	1	0	0	0	0	0	0	1	0	10	38	0	0	0	0	0	0	0
m	2,5	15	78	0	0	0	1	1	0	0	0	1	0	0	0	0	0	0	0	0	0	0	0	0	0	0	0	0	0	0	1	5	35	0	0	0	0	0	0	1
m	1,1	15	73	0	0	0	1	0	0	0	1	0	0	0	0	0	0	0	0	0	0	0	0	0	0	0	0	1	1	0	1				0	0	0	0	0	0
m	1,1	15	81	0	0	1	1	0	0	0	0	0	0	0	0	0	0	0	0	0	1	1	0	0	0	0	0	0	0	0	0	5	31	0	0	0	0	0	0	0
m	3,8	12	57	0	0	0	0	0	1	0	0	0	0	0	1	0	0	0	0	0	0	0	0	0	0	0	0	0	0	0	0	5	30	0	0	1	0	0	1	0
m	2,2	15	62	0	0	0	0	0	0	0	0	0	0	0	0	0	0	0	0	0	0	0	0	0	0	0	0	0	0	0	1	5	30	0	0	0	1	0	0	1
m	6,0	9	79	0	0	1	0	0	0	0	0	0	1	0	0	0	0	0	0	0	0	0	0	0	0	0	0	1	1	0	0	8	51	1	0	0	0	0	0	0
m	1,1	15	78	0	0	0	1	0	0	0	0	0	0	0	0	0	0	0	0	1	1	1	0	0	0	0	0	0	0	0	1	15	63	1	0	0	0	0	0	0
m	1,8	15	78	0	0	0	0	0	1	0	1	0	0	0	0	0	0	0	0	0	0	0	0	0	0	0	0	0	0	0	1	5	33	0	0	0	1	0	0	0
w	1,1	15	75	0	0	0	1	0	0	0	0	0	0	0	0	0	0	0	0	0	0	0	0	0	0	0	0	0	0	0	0	5	33	0	0	0	0	0	0	0

w	1,4	15	55	0	0	0	0	0	0	0	0	0	0	0	1	0	1	0	0	0	0	0	0	0	0	0	0	0	0	0	0	0	25	0	0	1	0	0	1	0
w	1,0	15	85	0	0	1	1	1	0	0	1	0	1	0	1	0	0	1	0	0	0	0	0	0	0	0	0	0	0	0	1	5	35	0	1	1	0	1	0	0
w	1,8	15	83	0	0	1	0	0	1	0	0	1	1	0	0	0	0	0	0	0	0	0	0	0	0	0	0	0	0	0	0	5	45	0	0	1	1	0	0	0
m	8,8	9	74	0	0	1	0	1	0	0	0	0	0	0	0	0	0	0	1	1	1	1	0	0	0	0	0	0	0	0	1	19	39	0	1	1	0	0	1	1
w	2,3	15	79	0	0	1	0	0	0	0	0	0	0	0	1	0	0	0	0	0	0	0	0	0	0	0	0	0	0	0	0	5	34	0	0	1	1	0	0	0
w	6,6	9	43	0	0	0	0	1	0	0	0	0	0	0	1	0	0	0	0	1	0	0	0	0	0	0	0	0	0	0	0	15	37	0	0	1	0	0	1	0
m	15,5	0	68	0	0	1	1	0	0	1	0	1	1	0	0	0	0	0	1	0	1	1	0	0	0	0	0	0	0	0	0	10	55	1	1	1	0	0	1	1
m	1,2	15	91	0	0	1	1	0	0	0	1	0	1	0	0	0	0	0	0	0	0	0	0	0	0	0	0	0	0	0	1	0	35	0	0	0	0	0	0	0
m	1,5	15	72	0	0	1	1	0	1	0	1	0	0	0	1	0	0	0	0	0	0	0	0	0	0	0	0	1	0	0	0	4	49	0	0	1	1	0	0	1
w	1,1	15	30	0	0	0	0	0	0	0	0	0	0	0	0	0	0	0	0	0	0	0	0	0	0	0	0	0	0	0	0	5	30	0	0	0	0	0	0	0
w	6,7	9	55	0	0	0	0	0	0	0	0	0	1	0	1	0	0	0	1	0	1	1	0	0	0	0	0	0	0	0	0	10	41	0	1	1	0	0	1	0
m	1,7	15	36	0	0	0	0	0	0	0	0	0	0	0	0	0	0	0	1	0	0	0	0	0	0	0	0	0	0	0	0	15	32	0	0	0	0	0	0	0
m	2,6	15	36	0	0	0	0	0	0	0	1	0	0	0	0	0	0	0	0	0	0	0	0	0	0	0	0	0	0	1	0	0	17	0	0	1	0	1	0	1
w	1,9	15	59	0	0	0	0	0	0	0	0	0	1	0	1	0	0	0	0	0	0	0	0	0	0	0	0	0	0	0	0	5	33	0	0	1	1	0	0	0
w	7,2	9	67	0	0	0	0	0	0	0	0	0	1	0	0	0	0	0	0	1	1	1	0	0	1	0	0	0	0	1	0	19	65	1	0	1	1	0	0	1
m	1,7	15	32	0	0	0	0	1	1	0	0	0	0	0	0	0	0	0	0	0	0	0	0	0	0	0	0	0	0	0	0	5	23	0	0	0	0	0	0	0
m	1,1	15	73	0	0	0	0	0	0	0	1	1	0	0	0	0	0	0	0	1	0	0	0	0	0	0	0	0	0	0	0	10	40	0	0	0	0	0	0	1
w	8,4	9	70	0	0	0	0	0	0	0	0	0	0	0	0	0	1	0	0	1	1	1	0	1	0	0	0	0	0	1	0	19	48	0	1	1	0	0	1	0
w	2,1	15	87	0	0	1	0	0	0	0	0	1	1	0	0	0	0	0	0	0	0	0	0	0	0	0	0	0	0	0	0	5	44	0	0	0	0	0	0	0
m	1,9	15	72	0	0	0	1	0	0	0	0	0	0	0	0	0	0	0	0	0	0	0	0	0	0	0	0	0	0	0	1	5	23	0	0	0	0	0	0	0
m	1,1	15	72	0	0	0	1	1	1	0	1	0	0	0	0	0	0	0	0	0	0	0	0	0	0	0	0	0	0	0	0	5	28	0	0	1	1	0	0	0
w	1,8	15	83	0	0	1	1	0	0	0	0	0	0	0	1	1	0	0	0	0	0	0	0	0	0	0	0	0	0	0	0	5	34	0	0	1	1	0	0	0
w	2,7	15	89	0	0	0	0	0	1	0	1	0	0	0	0	0	0	0	0	0	0	0	0	0	0	0	0	0	0	0	1	5	47	0	0	0	1	0	0	1
w	1,1	15	88	0	0	0	1	0	0	0	0	0	0	0	0	0	0	0	0	0	0	0	0	0	0	0	0	0	0	0	1	0	31	0	0	0	0	0	0	0
m	2,2	15	63	0	0	0	0	0	0	1	0	0	0	0	0	0	0	0	0	1	1	0	1	0	0	0	1	0	0	1	0	19	55	1	0	1	1	0	0	1
m	1,6	15	69	0	0	1	1	0	1	0	0	0	0	0	0	0	0	0	0	0	0	0	0	0	0	0	0	0	0	0	0	5	28	0	0	0	0	0	0	0
m	1,0	15	57	0	0	0	0	0	0	0	0	0	0	0	0	0	0	0	1	0	0	0	0	0	0	0	0	0	0	0	1	0	24	0	0	0	0	0	0	0
m	3,0	12	78	0	0	1	1	1	0	0	1	1	1	0	0	0	0	0	1	0	0	0	0	0	0	0	0	0	0	0	0	15	37	0	1	1	0	1	0	1
m	1,1	15	88	0	0	0	1	0	0	0	0	0	1	0	0	0	0	0	0	0	0	0	0	0	0	0	0	0	0	0	1	0	33	0	0	0	0	0	0	0
m	13,7	3	76	0	0	0	1	0	0	0	0	1	1	0	0	0	0	0	1	0	1	1	0	1	1	1	0	0	0	1	1	19	55	1	1	1	0	0	1	1
w	3,0	12	88	0	0	0	0	0	0	0	0	0	0	0	0	0	0	0	0	0	0	0	0	0	0	0	0	0	0	0	0	0	35	0	0	0	0	0	0	0
w	8,8	9	60	0	0	0	0	0	0	0	0	0	0	0	1	0	0	1	0	0	1	1	1	0	0	0	0	0	0	0	0	9	23	0	0	1	1	0	0	1
w	2,9	15	66	0	0	0	0	1	1	0	0	0	0	0	0	0	0	0	0	1	1	1	0	0	1	0	0	0	0	0	1	23	50	1	0	0	0	0	0	0
m	2,0	15	70	0	0	0	1	0	1	0	0	0	1	0	0	0	0	0	0	0	0	0	0	0	0	0	0	0	0	0	1	5	36	0	0	0	0	0	0	1
m	1,2	15	58	0	0	0	0	0	0	1	0	0	0	0	0	0	0	0	0	0	0	0	0	0	0	0	0	0	0	1	0	0	54	1	1	0	1	0	0	0
w	3,9	12	68	0	0	0	0	0	0	0	0	0	0	0	0	0	0	0	0	0	0	0	0	0	0	0	0	0	0	0	0	10	28	0	0	0	0	0	0	0
w	10,2	6	51	0	0	0	0	0	0	0	0	0	0	0	0	0	1	0	1	1	1	1	1	0	0	0	0	0	0	1	1	24	52	1	0	1	0	0	1	1
m	1,4	15	64	0	0	0	0	0	1	1	0	0	0	0	1	0	0	0	1	1	0	0	0	0	0	0	0	0	0	0	0	10	51	1	0	0	0	0	0	0
W	11,5	6	74	0	0	0	0	1	0	1	1	0	1	0	1	0	0	0	0	1	1	1	1	0	0	0	0	1	1	1	0	15	68	1	1	1	1	0	0	0
w	1,6	15	82	0	0	1	1	0	0	0	0	0	0	0	0	0	0	0	0	0	0	0	0	0	0	0	0	0	0	0	0	5	26	0	0	0	1	0	0	0
w	1,1	15	84	0	0	0	1	0	0	0	1	1	0	0	0	0	0	0	0	0	0	0	0	0	0	0	0	0	0	1	0	5	35	0	0	0	0	0	0	1
m	3,3	12	74	0	0	0	0	1	1	0	0	1	1	0	0	0	0	0	1	0	0	0	0	0	0	0	0	0	0	0	0	10	35	0	1	1	1	0	0	1
m	2,9	15	88	0	0	1	0	0	0	0	0	0	1	0	0	0	0	0	0	0	0	0	0	0	0	0	0	0	0	0	0	0	35	0	0	0	0	0	0	0
m	6,9	9	80	0	0	0	1	1	0	0	1	1	1	0	0	0	0	0	0	0	0	0	0	0	0	0	0	0	0	1	0	5	40	0	1	1	0	0	1	1
m	6,0	9	59	0	0	0	1	0	0	0	0	0	0	0	0	0	0	0	0	0	0	0	0	0	0	0	0	0	0	0	1	0	19	0	0	0	0	0	0	0
w	19,7	0	75	0	0	0	1	0	0	0	0	0	0	0	0	0	0	0	0	1	1	1	1	0	0	0	0	0	0	1	0	15	44	0	0	1	1	0	0	1
w	1,1	15	87	0	0	1	0	0	0	0	0	0	1	0	0	1	0	0	0	0	0	0	0	0	0	0	0	0	0	0	0	5	32	0	1	1	0	1	0	1
m	5,6	12	83	0	0	1	1	0	0	0	0	0	0	0	0	0	0	0	1	0	1	1	1	0	0	0	0	0	0	0	0	15	54	1	0	1	1	0	0	1
m	1,2	15	67	0	0	1	1	0	0	0	0	0	0	1	1	0	0	0	0	0	0	0	0	0	0	0	0	0	0	0	0	0	27	0	1	1	1	0	0	0
m	3,6	12	69	0	0	1	1	0	0	0	0	0	0	1	0	0	0	0	0	0	1	1	0	0	0	0	0	0	0	1	0	5	56	1	0	1	0	0	1	1
m	1,0	15	86	0	0	1	0	0	1	0	0	0	0	0	0	0	0	0	0	0	0	0	0	0	0	0	0	0	0	0	1				0	0	0	0	0	0
